# Supplementary material for: From word models to executable models of signaling networks using automated assembly
Source: Mol Syst Biol. 2017 Nov 24;13(11):954. doi: 10.15252/msb.20177651 (PMC5731347; doi:10.15252/msb.20177651)
Supplement: Supplementary file 1 — Appendix [file MSB-13-954-s001.pdf]

From word models to executable models of signaling networks using automated assembly

Appendix

B. M. Gyori[1,*], J. A. Bachman[1,*], K. Subramanian[1], J. L. Muhlich[1], L. Galescu[2], P. K. Sorger[1,+]

[1] Laboratory of Systems Pharmacology, Harvard Medical School, 200 Longwood Ave, Boston MA 02115, USA
[2] Institute for Human and Machine Cognition, Pensacola FL 32502, USA
[*] Authors contributed equally to this work
[+] To whom correspondence should be addressed

Email: Peter Sorger - peter_sorger@hms.harvard.edu;

Contents

1 Appendix Figures **2**

2 Appendix Methods **12**

2.1 The TRIPS/DRUM natural language processing system 12
2.2 Querying databases to extract INDRA Statements 13
2.3 Modeling alternative dynamical patterns of p53 activation 14
2.4 Modeling resistance to targeted therapy by vemurafenib 15
2.5 An extensible and executable map of the RAS pathway 20

References **21**

Appendix Notebook 1 **22**

Appendix Notebook 2 **27**

INDRA Software Documentation **33**

1 Appendix Figures

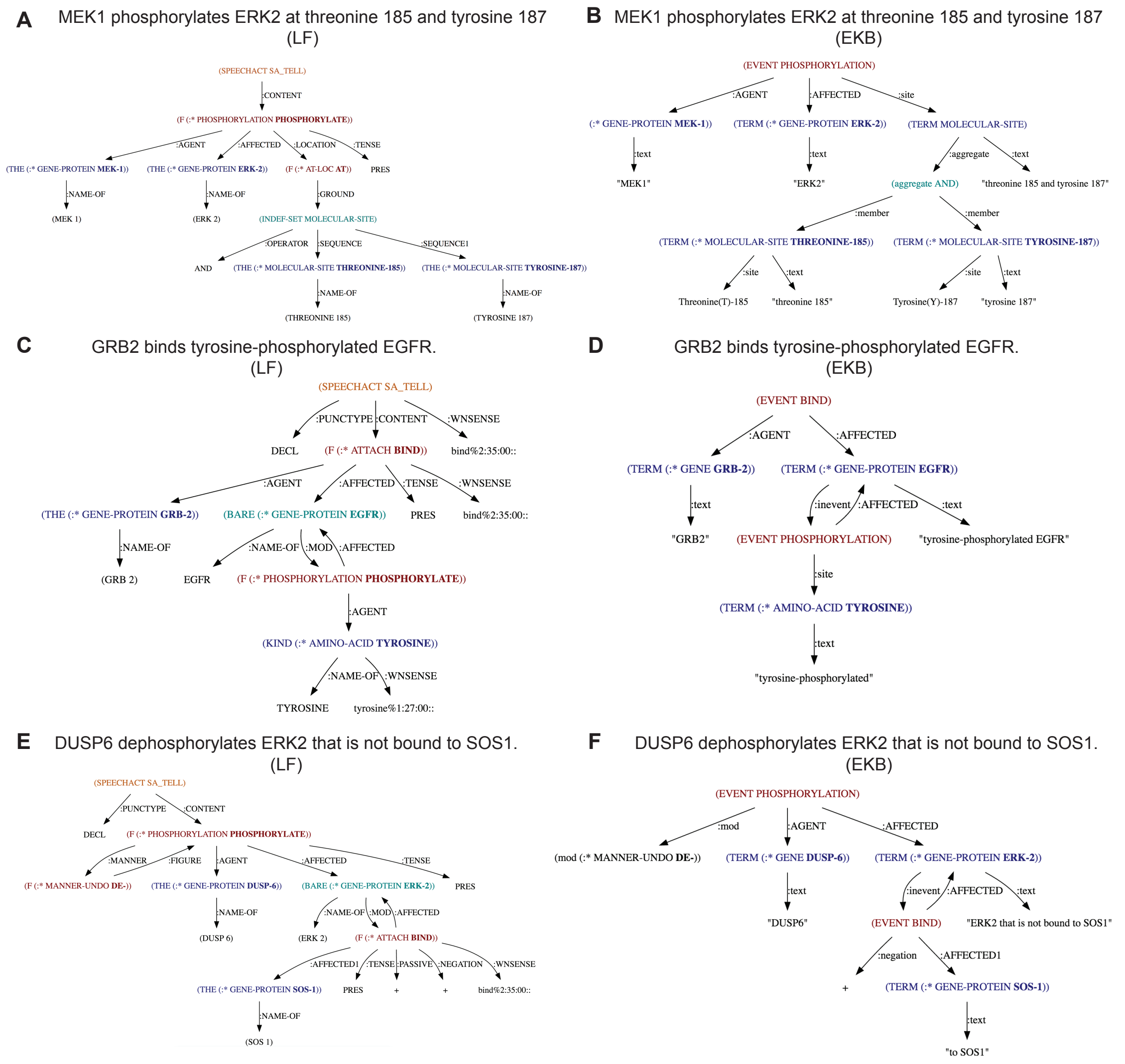

Figure S1. TRIPS logical form (LF) and extraction knowledge base (EKB) graphs.
(A) The LF graph for the sentence "MEK1 phosphorylates ERK2 at threonine 185 and tyrosine 187".
(B) The EKB graph for the sentence "MEK1 phosphorylates ERK2 at threonine 185 and tyrosine 187".
(C) The LF graph for the sentence "GRB2 binds tyrosine-phosphorylated EGFR".
(D) The EKB graph for the sentence "GRB2 binds tyrosine-phosphorylated EGFR" shows the main BIND event with GRB2 and EGFR as its arguments. EGFR is further affected by a PHOSPHORYLATION sub-event with the site specified as a tyrosine amino-acid residue.
(E) The LF graph for the sentence "DUSP6 dephosphorylates ERK2 that is not bound to SOS1".
(F) The EKB graph for the sentence "DUSP6 dephosphorylates ERK2 that is not bound to SOS1" has a main event PHOSPHORYLATION which is negated by a modifier to represent dephosphorylation and which has ERK2 affected by DUSP6 as its arguments. ERK2 is further affected by a negated binding sub-event with SOS1.

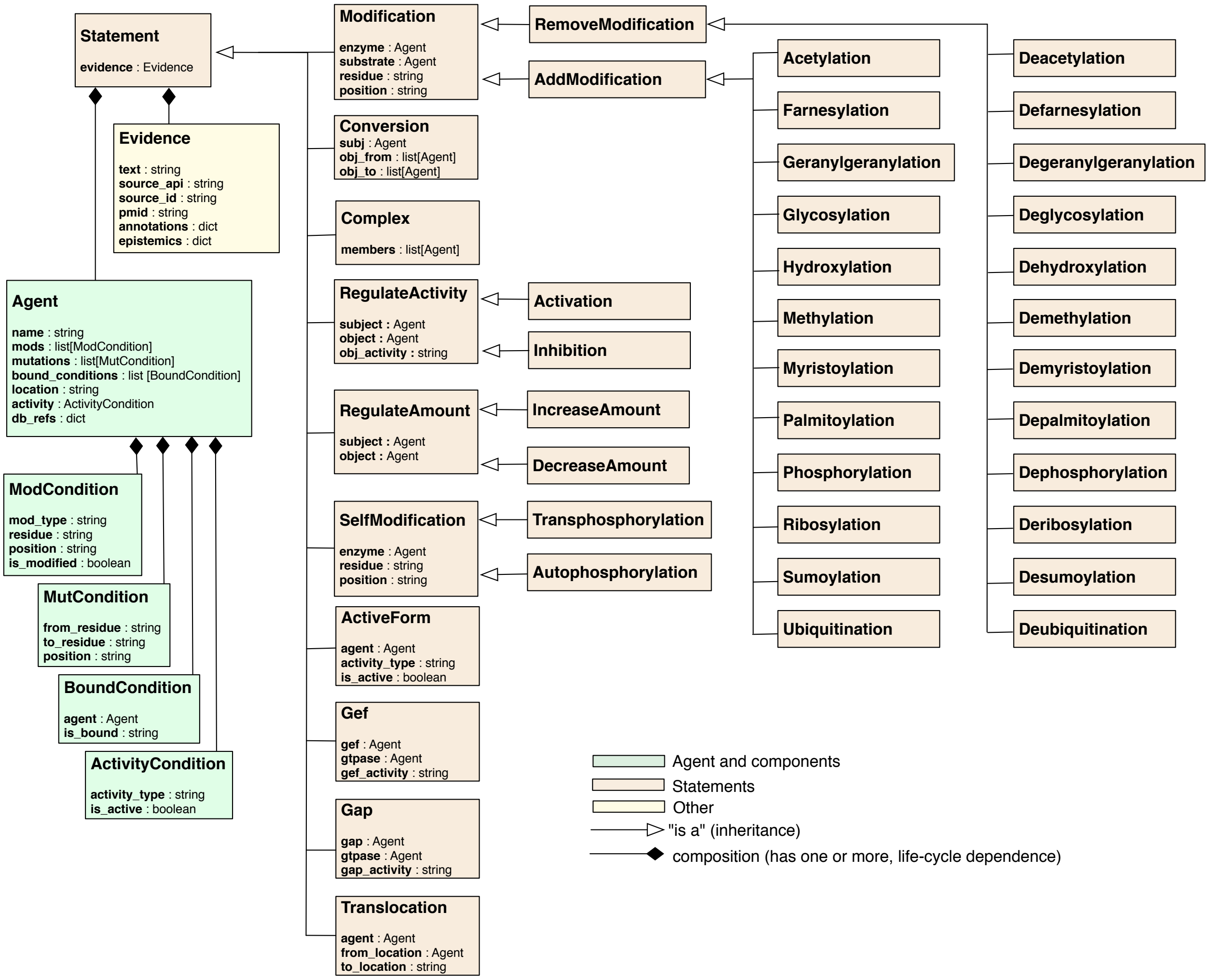

Figure S2. Unified Modeling Language (UML) diagram of INDRA Statements and associated classes. All INDRA Statements inherit from the Statement class. Each Statement has one or more Agents associated with them. An Agent represents molecular context through additional attributes (e.g. location) and associated classes including ModCondition (for post-translational modifications), MutCondition (for mutations), BoundCondition (for bound co-factors) and ActivityCondition (for active/inactive state). Some Statement types also have attributes (e.g. residue, position) in addition to their Agent arguments. Each Statement has one or more Evidences associated with it.

A

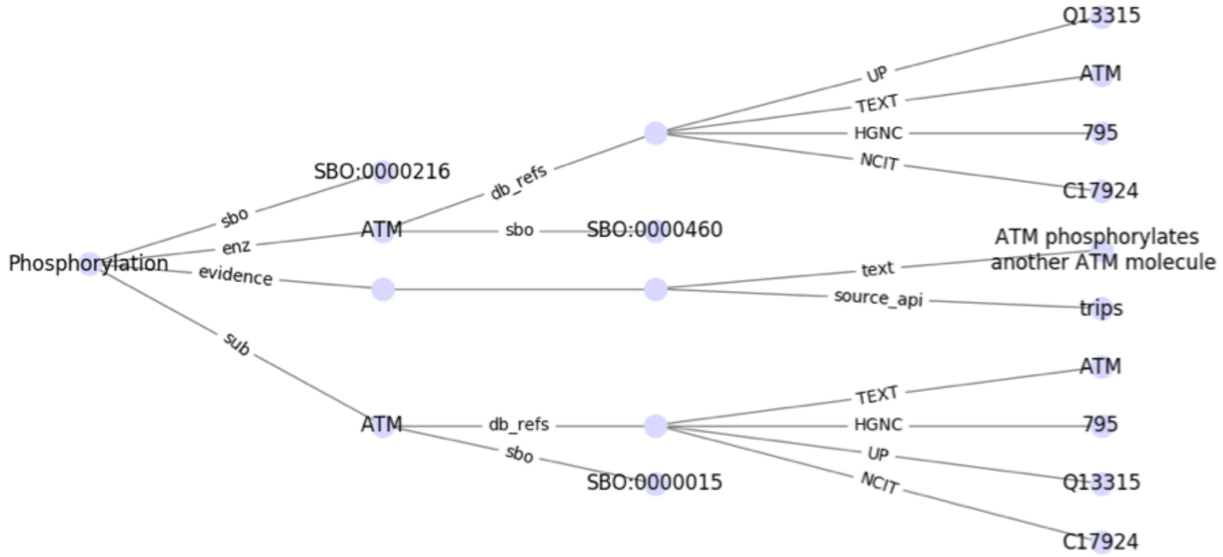

B


```
{
 "type": "Phosphorylation",
 "enz": {
  "name": "ATM",
  "activity": {
   "activity_type": "activity",
   "is_active": true
  },
  "db_refs": {
   "TEXT": "ATM",
   "HGNC": "795",
   "UP": "Q13315",
   "NCIT": "C17924"
  },
  "sbo": "http://identifiers.org/sbo/SBO:0000460"
 },
 "sub": {
  "name": "ATM",
  "db_refs": {
   "TEXT": "ATM",
   "HGNC": "795",
   "UP": "Q13315",
   "NCIT": "C17924"
  },
  "sbo": "http://identifiers.org/sbo/SBO:0000015"
 },
 "evidence": [
  {
   "source_api": "trips",
   "text": "Active ATM phosphorylates another ATM molecule."
  }
 ],
 "id": "cb1fae70-41b8-4be0-b1d2-aa8ebcbb20a3",
 "sbo": "http://identifiers.org/sbo/SBO:0000216"
}
```


Figure S3. INDRA Statement visualized as graph and serialized as JSON. INDRA allows displaying the data structure of Statements as graphs (A), and provides a JSON exchange format (B) for inspection and reuse in a platform-independent way.

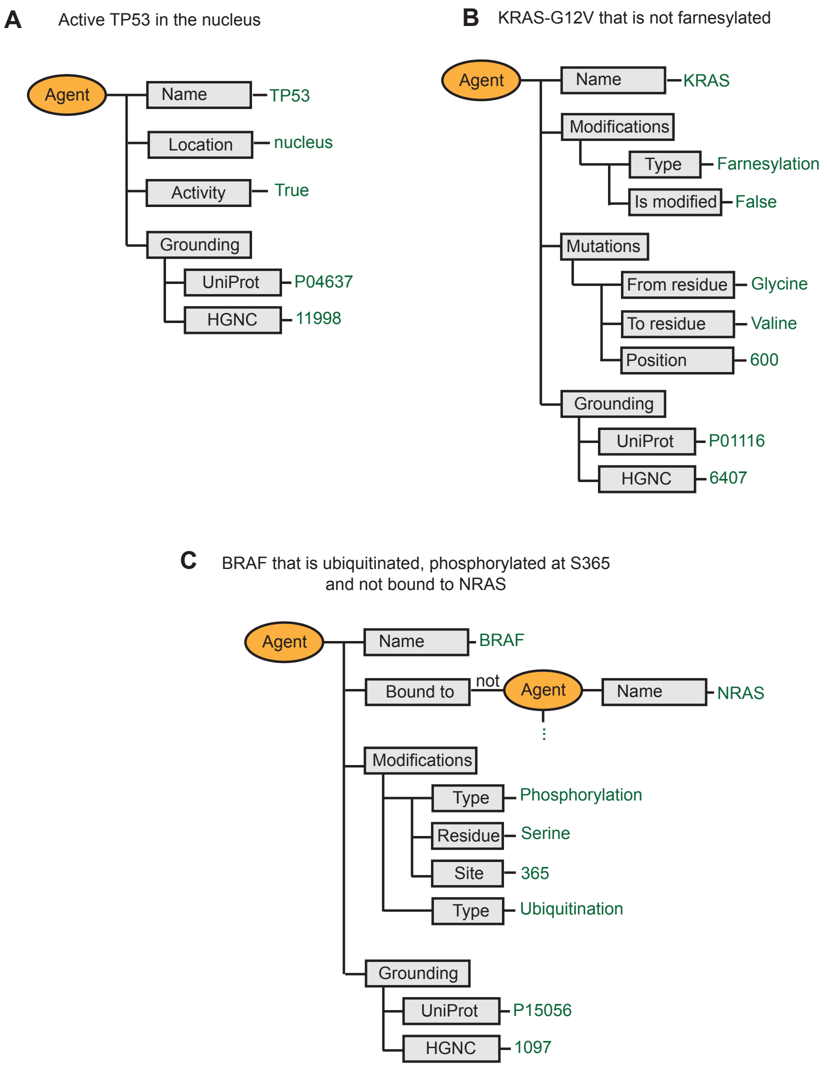

Figure S4. Examples of INDRA Agents representing molecular state.
(A) Agent representing *active TP53 in the nucleus*
(B) Agent representing *KRAS-G12V that is not farnesylated*
(C) Agent representing *BRAF that is ubiquitinated, phosphorylated at S365 and not bound to NRAS.*

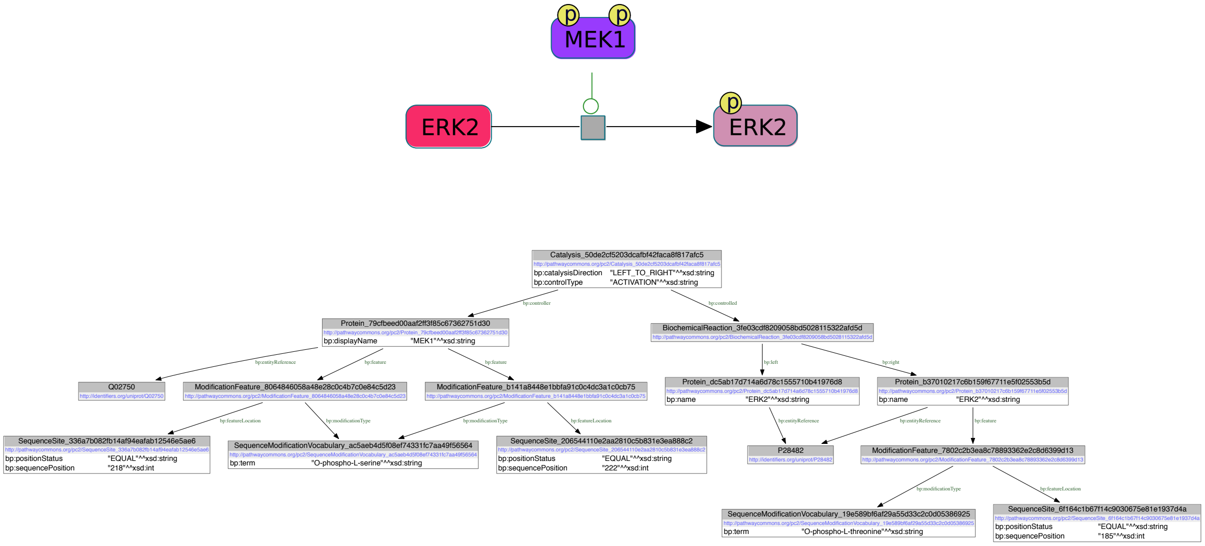

Figure S5. BioPAX representations of the mechanism *double-phosphorylated MEK1 phosphorylates ERK2* as a ChIBE (Babur *et al*, 2010) graph (above) and an object model graph (below).

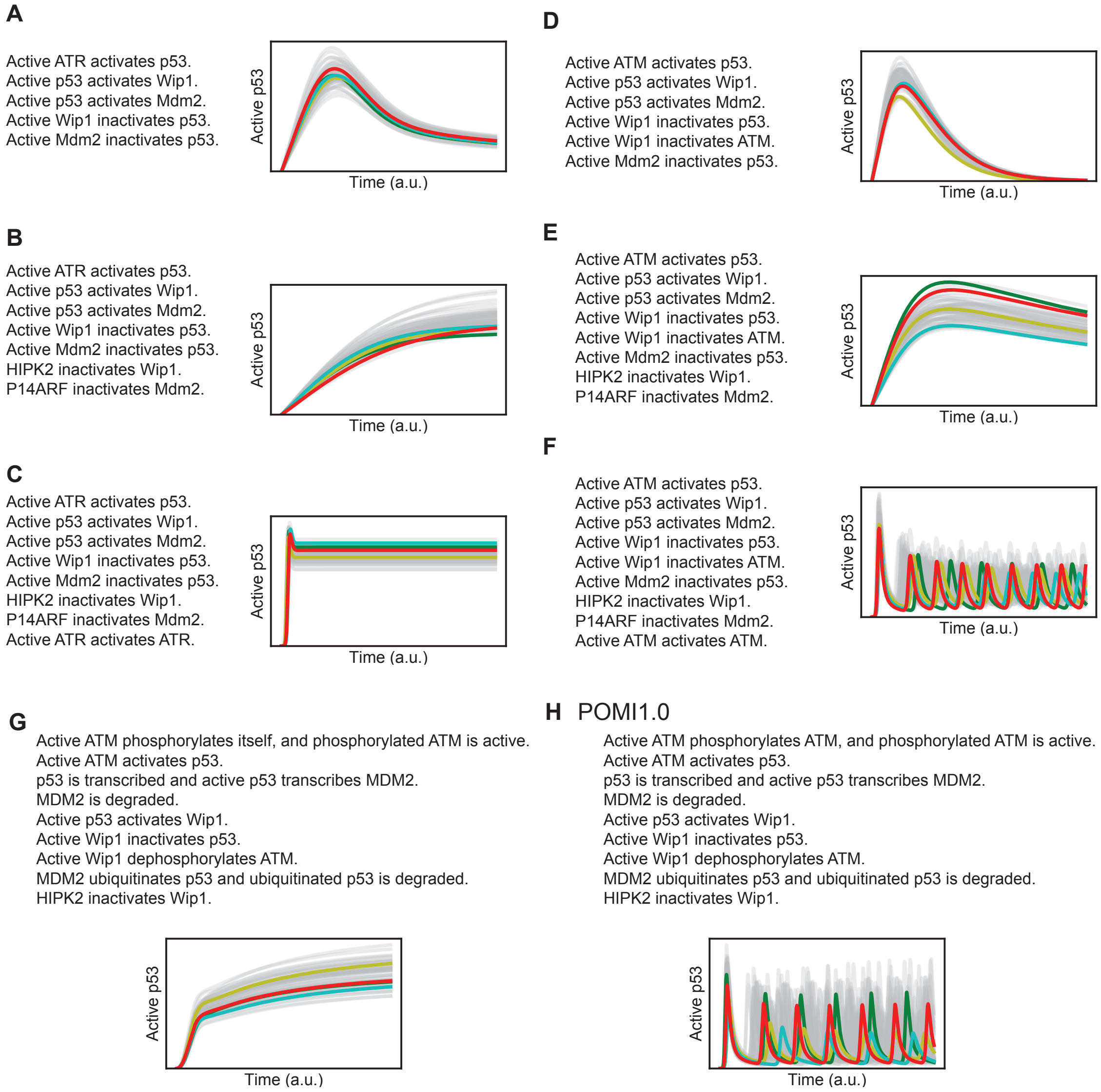

Figure S6. The dynamics of active p53 under 100 simulation conditions (gray, some samples highlighted in colors), with parameters randomly sampled from uncorrelated log-normal distributions around their nominal values. The simulation dynamics with nominal parameters are shown in red. (A) SSB model from yellow numbered edges in Figure 5A.
(B) SSB model with Wip1 and Mdm2 inactivation added.
(C) SSB model with ATR activation added.
(D) DSB model from yellow numbered edges in Figure 5A.
(E) DSB model with Wip1 and Mdm2 inactivation added.
(F) DSB model with ATM activation added.
(G) Detailed DSB model with ATM cis-autophosphorylation.
(H) POMI1.0: Detailed DSB model with ATM trans-autophosphorylation.

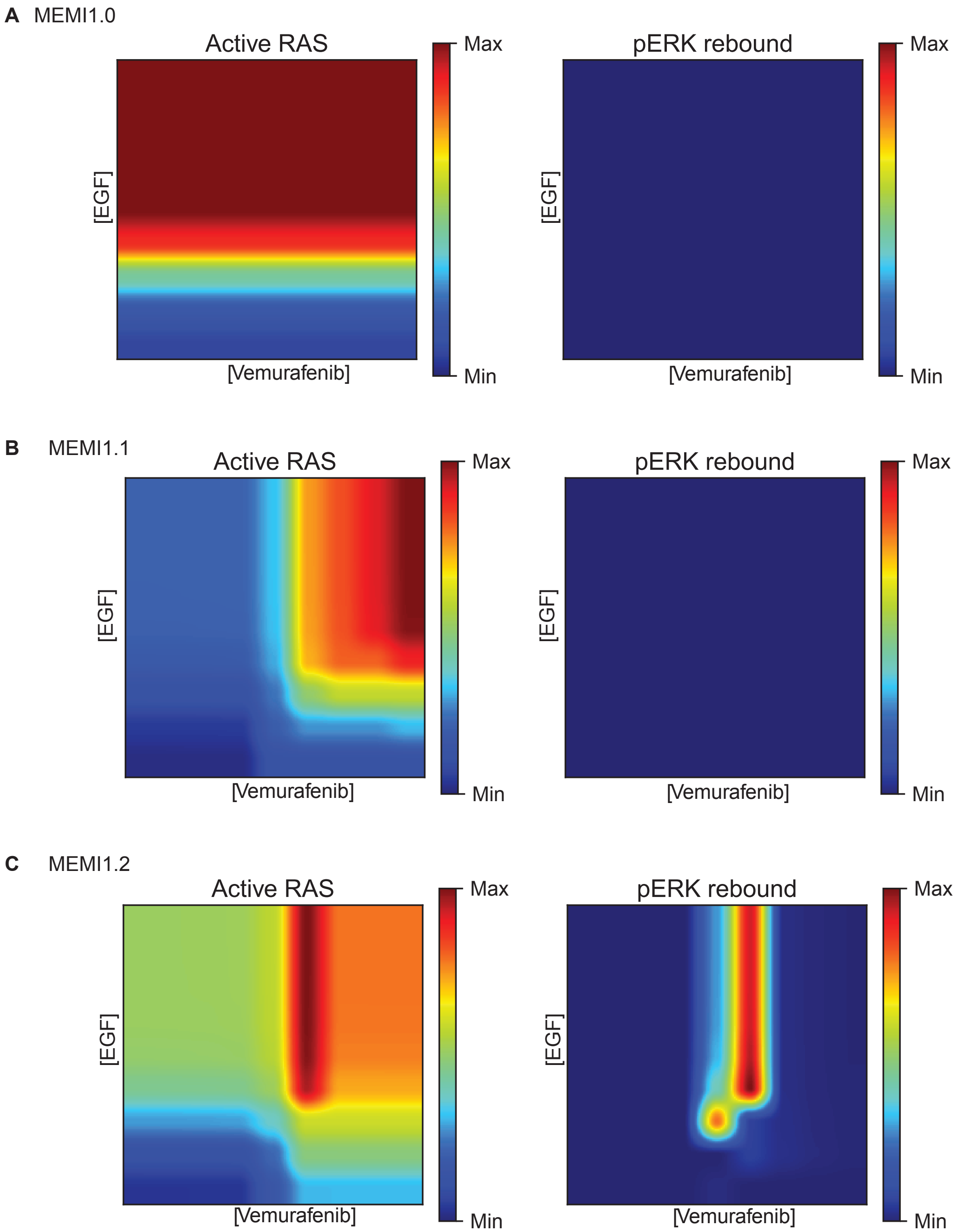

Figure S7. Heat maps showing steady state values of active RAS (left) and phospho-ERK rebound (right) upon varying doses of EGF and vemurafenib.
(A) Dose response heat maps for MEMI1.0 (B) Dose response heat maps for MEMI1.1 (C) Dose response heat maps for MEMI1.2

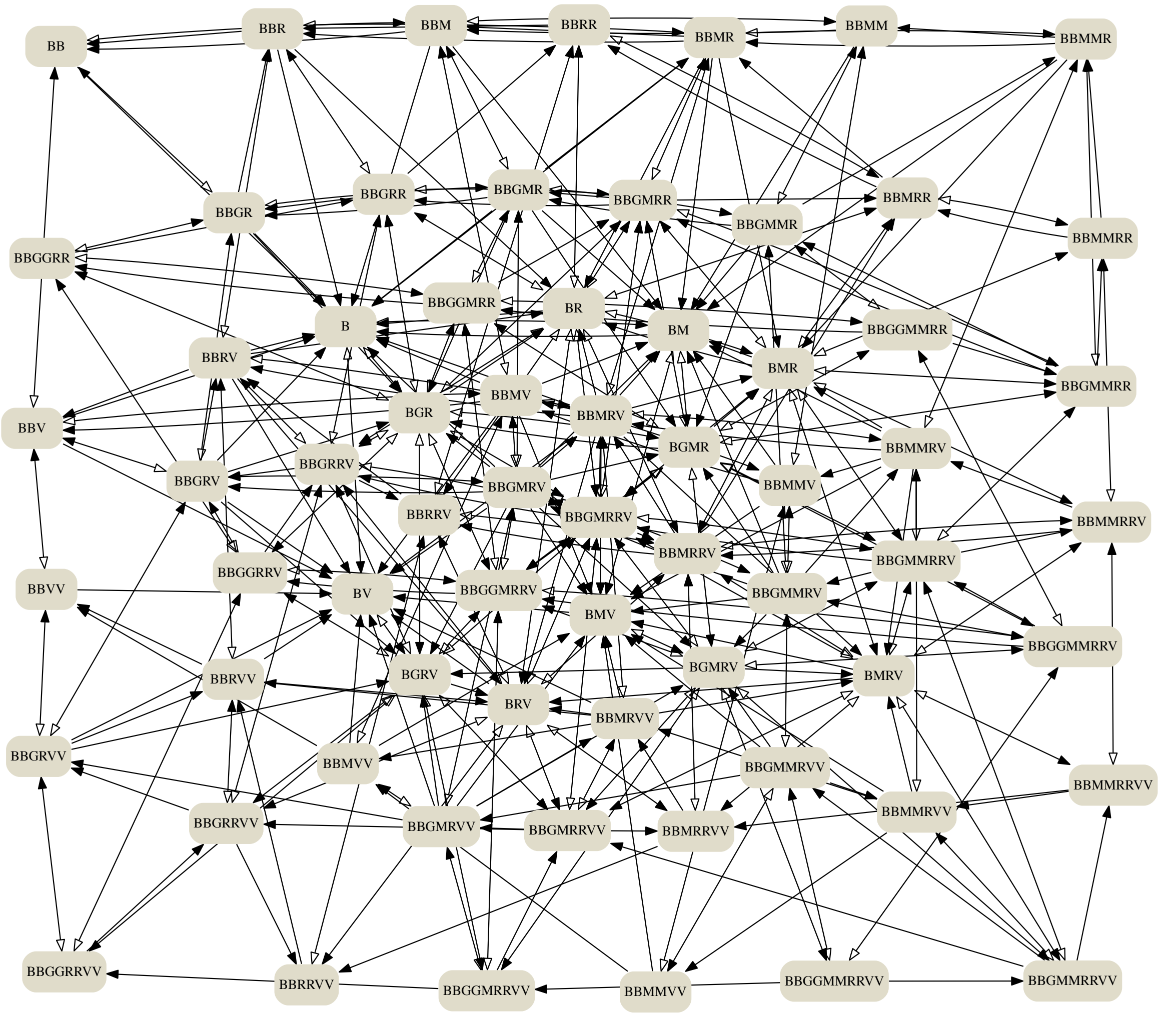

Figure S8. Biochemical reactions involving BRAF in the MEMI1.2 model. Each node corresponds to an individual molecular species with the label composed of the first letters of the monomers the species consists of: BRAF (B), RAS (R), GTP (G), vemurafenib (V) and MEK (M). Each directed edge corresponds to a reaction which produces the target species from the source species. The network visualization is simplified as it omits modification states and merges complexes with a given set of constituents that have different topologies.

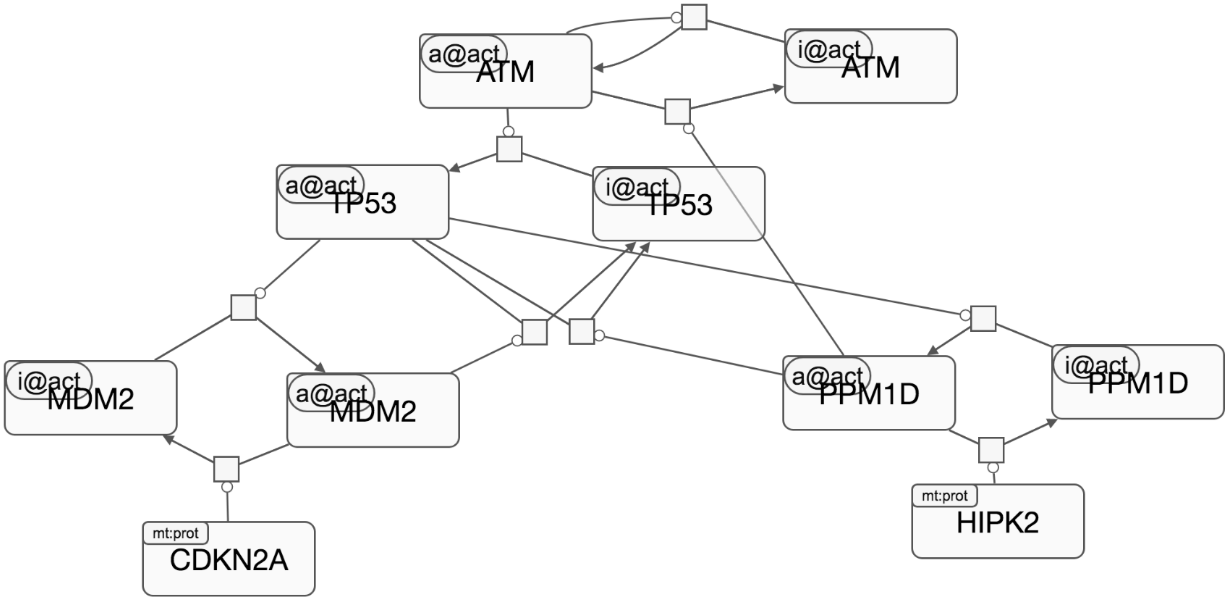

Figure S9. INDRA-assembled SBGN model of p53 activation upon DSB. This model was assembled from the natural language shown in Figure 5C (main text) using INDRA's SBGN Assembler module. The SBGN graph was visualized and layout was set manually in the SBGNViz online editor (Sari *et al*, 2015)

.

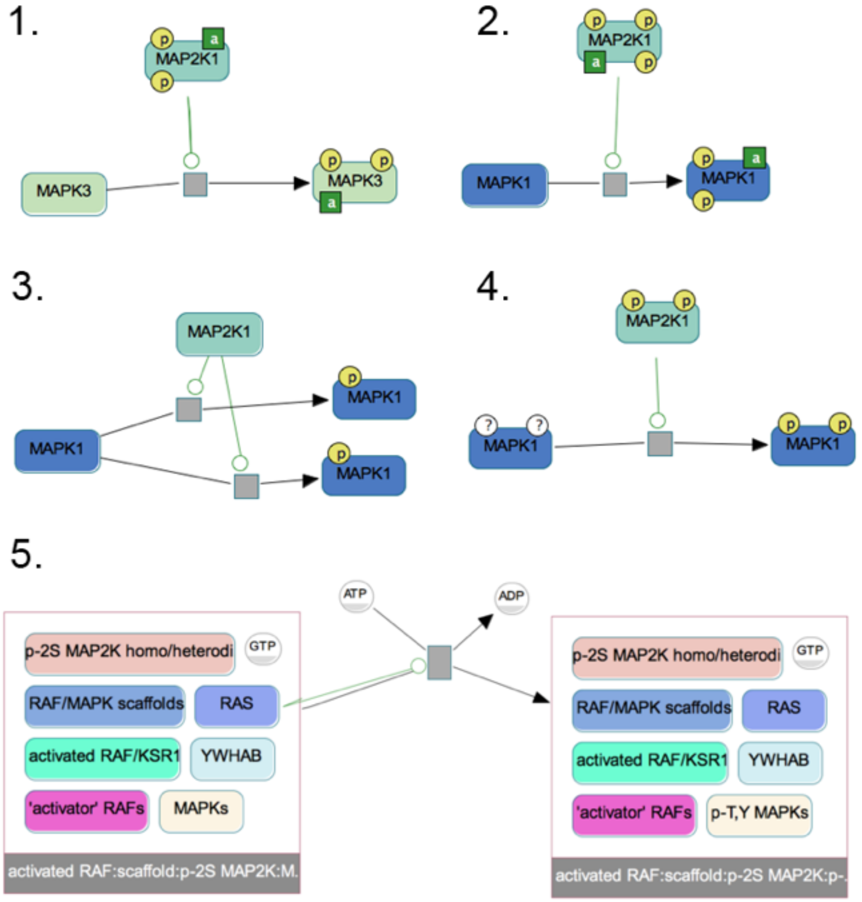

Figure S10. Selected BioPAX BiochemicalReactions involving MAPK1/3 and MAP2K1 in Pathway Commons, displayed using the Chisio BioPAX Editor (ChIBE) (Babur *et al*, 2010).

2 Appendix Methods

2.1 The TRIPS/DRUM natural language processing system

In this section we provide more technical details on the TRIPS/DRUM system (Allen *et al*, 2015) which INDRA uses to process natural language text (note that in the main text we refer to the system as TRIPS). The DRUM (Deep Reader for Understanding Mechanisms) system is an instance of the TRIPS general deep language understanding system (Allen *et al*, 2008), customized for reading and understanding scientific text in molecular biology.

To process natural language input, first a suite of shallow NLP tools performs annotations on the text, including: a) sentence splitting; b) named entity recognition; c) derivational analysis (e.g., prefixes, pertainyms); and d) statistical parsing. Critically, DRUM uses a number of domain-specific named entity taggers to identify genes, proteins, protein families, drugs and chemicals, cell lines, diseases, etc.; successful matches are annotated with IDs in the original resources, and are also mapped to internal ontology types. At this stage all matches are annotated; there is no attempt to disambiguate among alternatives. Other domain-specific annotators use regular expressions to identify, for example, molecular sites and mutations. Statistical parsers – CoreNLP (Manning *et al*, 2014) and Enju (Hara *et al*, 2005) – are used to find constituent boundaries; only those constituents on which both parsers agree are used.

The output of all these specialized preprocessors is sent to the TRIPS parser, which uses it as advice during its search for the optimal parse of each sentence. The TRIPS parser is at the core of our approach; it uses a hand-built lexicalized context-free grammar, augmented with feature structures and feature unification, and domain-general selectional restrictions (encoded in the lexicon and ontology) to eliminate semantically anomalous sense combinations. The parser constructs from the input a logical form (LF), which is a semantic representation that captures an unscoped modal logic (Allen *et al*, 1995; Manshadi *et al*, 2008). The logical form includes the surface speech act, semantic types, semantic roles for predicate arguments, and dependency relations. Lexical entities in the LF represent word senses and ontology types, as well as tense, modality and aspect information – information that is crucial for determining, for example, whether a statement expresses a stated fact, a conjecture or a possibility. The parser draws on a general purpose semantic lexicon and ontology which define a range of word senses and lexical semantic relations. The core semantic lexicon was constructed by hand and contains approximately $7,500$ lemmas (generating approximately three times that many words) and $2,000$ concepts in the ontology. The ontology is organized hierarchically and each ontology concept has associated with it possible semantic roles and selectional preferences that further refine the concept. The core lexicon is extended to cover virtually all words in WordNet (Fellbaum, 2010) by adding lexical entries with plausible semantic and syntactic structures through a dynamic mapping between the WordNet hypernym hierarchy and that of the TRIPS ontology. Ontology-based lexical expansion – using WordNet as well as all the terminology extracted from the biological resources – is the main tool by which we can customize our generic, relatively low coverage semantic parser to attain the broad coverage needed to process text in as lexically rich a domain as that of molecular biology.

Finally, the LFs produced by the parser are used to extract the content relevant for the domain, in this case concepts (e.g., molecular entities), events (e.g., activation, modification) involving those

concepts, causal relations between events, as well as additional information about events, such as polarity, modality (e.g., possibility, necessity, various epistemic and evidential modals). Because much of the variation and complexity in sentence constructions is handled by the TRIPS parser, and the LF terms are grounded in the TRIPS ontology, we are able to use a relatively compact rule set for defining the events and relationships of interest. The extraction rules were developed from general principles rather than based on specific training samples; thus, even though most of the text used for development was extracted from papers on the Ras signaling pathways, we expect the system to have good performance on any input describing biomolecular mechanisms.

2.2 Querying databases to extract INDRA Statements

To benchmark the ability of INDRA to extract *Statements* from pathway databases, we used INDRA's BioPAX and BEL APIs to search for the neighborhood of 20 genes or metabolites in Pathway Commons and the BEL Large Corpus, respectively. In the case of BioPAX we counted the total number of controlled BiochemicalReactions and TemplateReactions (used to represent gene transcription) as reference, and in the case of BEL we counted all direct statements (i.e. with predicate directlyIncreases/directlyDecreases). We then determined how many of these source entries in each case were extracted and represented as INDRA *Statements*, as constructed by INDRA's BioPAX and BEL *Processors*. We selected 5 representative genes or metabolites from signaling, gene regulation, metabolism (protein controller) and metabolism (metabolite) to perform the analysis. The results of this analysis are shown in Table S1.

Gene / metabolite	BioPAX total	BioPAX extracted	BioPAX %	BEL total	BEL extracted	BEL %
AKT1	1341	1182	88%	38	35	92%
MAPK1	1683	1555	92%	110	110	100%
CTNNB1	283	177	63%	32	17	53%
GNAS	73	47	64%	14	13	93%
JAK1	491	232	47%	15	15	100%
STAT3	386	313	81%	43	33	77%
FOXO3	393	383	98%	43	37	86%
TP53	963	919	95%	75	66	88%
JUN	3915	3881	99%	23	17	74%
MYC	2947	2942	100-%	21	17	81%
DHFR	33	28	85%	0	-	-
NOS1	31	25	81%	5	3	60%
GLUL	32	27	84%	0	-	-
PFKL	18	13	72%	2	2	100%
IDH1	27	17	63%	0	-	-
glutamine (CHEBI:28300)	11	11	100%	0	-	-
β-D-fructofuranose-6-phosphate (CHEBI:16084)	13	12	92%	0	-	-
5,6,7,8-tetrahydrofolic acid (CHEBI:20506)	15	6	40%	0	-	-
pyruvic acid (CHEBI:32816)	82	36	44%	0	-	-
nitric oxide (CHEBI:16480)	16	8	50%	0	-	-

Table S1. Statistics for the extraction of INDRA Statements from Pathway Commons (in BioPAX format) and the BEL Large Corpus. Molecules are grouped into four categories with 5 test examples each: signaling, gene regulation, metabolism (protein controller) and metabolism (metabolite). *BioPAX total* and *BEL total* show the number of entries in each source around the neighborhood of the given molecule.

2.3 Modeling alternative dynamical patterns of p53 activation

Tables S2–S4 list the PySB rules and the associated kinetic rates constituting the three models for the activation dynamics of p53 with Table S2 corresponding to the model in Figure 5(B), Table S3 to the model in Figure 5(C) and Table S4 to the model in Figure 5(E).

Rule	Forward kinetic rate
ATR(activity='active') + TP53(activity='inactive') >> ATR(activity='active') + TP53(activity='active')	10^{-7} molec$^{-1}s^{-1}$
TP53(activity='active') + PPM1D(activity='inactive') >> TP53(activity='active') + PPM1D(activity='active')	10^{-7} molec$^{-1}s^{-1}$
TP53(activity='active') + MDM2(activity='inactive') >> TP53(activity='active') + MDM2(activity='active')	10^{-7} molec$^{-1}s^{-1}$
ATR(activity='active') + ATR(activity='inactive') >> ATR(activity='active') + ATR(activity='active')	$5 \cdot 10^{-7}$ molec$^{-1}s^{-1}$
PPM1D(activity='active') + TP53(activity='active') >> PPM1D(activity='active') + TP53(activity='inactive')	$5 \cdot 10^{-7}$ molec$^{-1}s^{-1}$
MDM2(activity='active') + TP53(activity='active') >> MDM2(activity='active') + TP53(activity='inactive')	10^{-7} molec$^{-1}s^{-1}$
HIPK2() + PPM1D(activity='active') >> HIPK2() + PPM1D(activity='inactive')	10^{-7} molec$^{-1}s^{-1}$
CDKN2A() + MDM2(activity='active') >> CDKN2A() + MDM2(activity='inactive')	10^{-7} molec$^{-1}s^{-1}$

Table S2. Rules and parameters for the SSB (ATR-driven) p53 activation model.

Rule	Forward kinetic rate
ATM(activity=’active’) + TP53(activity=’inactive’) >> ATM(activity=’active’) + TP53(activity=’active’)	10^{-7} molec$^{-1}s^{-1}$
TP53(activity=’active’) + PPM1D(activity=’inactive’) >> TP53(activity=’active’) + PPM1D(activity=’active’)	10^{-7} molec$^{-1}s^{-1}$
TP53(activity=’active’) + MDM2(activity=’inactive’) >> TP53(activity=’active’) + MDM2(activity=’active’)	10^{-7} molec$^{-1}s^{-1}$
ATM(activity=’active’) + ATM(activity=’inactive’) >> ATM(activity=’active’) + ATM(activity=’active’)	$5 \cdot 10^{-7}$ molec$^{-1}s^{-1}$
PPM1D(activity=’active’) + TP53(activity=’active’) >> PPM1D(activity=’active’) + TP53(activity=’inactive’)	$5 \cdot 10^{-7}$ molec$^{-1}s^{-1}$
PPM1D(activity=’active’) + ATM(activity=’active’) >> PPM1D(activity=’active’) + ATM(activity=’inactive’)	10^{-5} molec$^{-1}s^{-1}$
MDM2(activity=’active’) + TP53(activity=’active’) >> MDM2(activity=’active’) + TP53(activity=’inactive’)	10^{-7} molec$^{-1}s^{-1}$
HIPK2() + PPM1D(activity=’active’) >> HIPK2() + PPM1D(activity=’inactive’)	10^{-7} molec$^{-1}s^{-1}$
CDKN2A() + MDM2(activity=’active’) >> CDKN2A() + MDM2(activity=’inactive’)	10^{-7} molec$^{-1}s^{-1}$

Table S3. Rules and parameters for the DSB (ATM-driven) p53 activation model.

Rule	Forward kinetic rate
ATM(phospho=’p’) + ATM(phospho=’u’) >> ATM(phospho=’p’) + ATM(phospho=’p’)	$5 \cdot 10^{-7}$ molec$^{-1}s^{-1}$
PPM1D(activity=’active’) + ATM(phospho=’p’) >> PPM1D(activity=’active’) + ATM(phospho=’u’)	10^{-5} molec$^{-1}s^{-1}$
MDM2() + TP53(ub=’n’) >> MDM2() + TP53(ub=’y’)	1e-06 molec$^{-1}s^{-1}$
ATM(phospho=’p’) + TP53(activity=’inactive’) >> ATM(phospho=’p’) + TP53(activity=’active’)	10^{-7} molec$^{-1}s^{-1}$
TP53(activity=’active’) + PPM1D(activity=’inactive’) >> TP53(activity=’active’) + PPM1D(activity=’active’)	10^{-7} molec$^{-1}s^{-1}$
PPM1D(activity=’active’) + TP53(activity=’active’) >> PPM1D(activity=’active’) + TP53(activity=’inactive’)	$5 \cdot 10^{-7}$ molec$^{-1}s^{-1}$
HIPK2() + PPM1D(activity=’active’) >> HIPK2() + PPM1D(activity=’inactive’)	10^{-7} molec$^{-1}s^{-1}$
MDM2() >> None	$8 \cdot 10^{-2}$ s^{-1}
TP53(ub=(y’) >> None	$2 \cdot 10^{-5}$ s^{-1}
None >> TP53(ub=’n’, activity=’inactive’)	2 molec s^{-1}
TP53(activity=’active’) >> TP53(activity=’active’) + MDM2()	$2 \cdot 10^{-2}$ s^{-1}

Table S4. Rules and parameters for the detailed DSB (ATM-driven) p53 activation model (POMI1.0).

2.4 Modeling resistance to targeted therapy by vemurafenib

Tables S5-S7 list the PySB rules and the corresponding kinetic rate constants for the MEMI1.0-1.2 models. Table S8 shows the total amounts of proteins (initial conditions) used in each of the three models.

Rule	Forward rate
EGF(erbb=None) + EGFR(egfr_ligand=None) >> EGF(erbb=1) % EGFR(egfr_ligand=1)	1.0 molec$^{-1}s^{-1}$
EGF(erbb=1) % EGFR(egfr_ligand=1) >> EGF(erbb=None) + EGFR(egfr_ligand=None)	0.1 s^{-1}
EGFR(egfr_ligand=ANY, erbb=None) + EGFR(egfr_ligand=ANY, erbb=None) >> EGFR(egfr_ligand=ANY, erbb=1) % EGFR(egfr_ligand=ANY, erbb=1)	1.0 molec$^{-1}s^{-1}$
EGFR(erbb=1) % EGFR(erbb=1) >> EGFR(erbb=None) + EGFR(erbb=None)	0.1 s^{-1}
EGFR(erbb=ANY, grb2=None) + GRB2(erbb=None) >> EGFR(erbb=ANY, grb2=1) % GRB2(erbb=1)	1.0 molec$^{-1}s^{-1}$
EGFR(grb2=1) % GRB2(erbb=1) >> EGFR(grb2=None) + GRB2(erbb=None)	0.1 s^{-1}
GRB2(erbb=ANY, sos=None) + SOS(grb2=None) >> GRB2(erbb=ANY, sos=1) % SOS(grb2=1)	1.0 molec$^{-1}s^{-1}$
GRB2(sos=1) % SOS(grb2=1) >> GRB2(sos=None) + SOS(grb2=None)	0.1 s^{-1}
SOS(grb2=ANY, ras=None) + RAS(map3k=None, sos=None) >> SOS(grb2=ANY, ras=1) % RAS(map3k=None, sos=1)	1.0 molec$^{-1}s^{-1}$
SOS(ras=1) % RAS(sos=1) >> SOS(ras=None) + RAS(sos=None)	50.0 s^{-1}
RAS(sos=ANY, gtp=None) + GTP(ras=None) >> RAS(sos=ANY, gtp=1) % GTP(ras=1)	50.0 molec$^{-1}s^{-1}$
RAS(gtp=1) % GTP(ras=1) >> RAS(gtp=None) + GTP(ras=None)	0.5 s^{-1}
RAS(map3k=None, sos=None, gtp=ANY) + BRAF(ras=None, V600=’E’) >> RAS(map3k=1, sos=None, gtp=ANY) % BRAF(ras=1, V600=’E’)	1.0 molec$^{-1}s^{-1}$
RAS(map3k=1) % BRAF(ras=1, V600=’E’) >> RAS(map3k=None) + BRAF(ras=None, V600=’E’)	0.5 s^{-1}
VEMURAFENIB(map3k=None) + BRAF(V600=’E’, vemurafenib=None) >> VEMURAFENIB(map3k=1) % BRAF(V600=’E’, vemurafenib=1)	10.0 molec$^{-1}s^{-1}$
VEMURAFENIB(map3k=1) % BRAF(V600=’E’, vemurafenib=1) >> VEMURAFENIB(map3k=None) + BRAF(V600=’E’, vemurafenib=None)	1.0 s^{-1}
PPP2CA(map2k=None) + MEK(mapk=None, phospho=’p’, ppp2=None) >> PPP2CA(map2k=1) % MEK(mapk=None, phospho=’p’, ppp2=1)	1.0 molec$^{-1}s^{-1}$
PPP2CA(map2k=1) % MEK(mapk=None, phospho=’p’, ppp2=1) >> PPP2CA(map2k=None) + MEK(mapk=None, phospho=’u’, ppp2=None)	10.0 s^{-1}
PPP2CA(map2k=1) % MEK(ppp2=1) >> PPP2CA(map2k=None) + MEK(ppp2=None)	0.001
MEK(mapk=None, phospho=’p’, ppp2=None) + ERK(map2k=None, phospho=’u’) >> MEK(mapk=1, phospho=’p’, ppp2=None) % ERK(map2k=1, phospho=’u’)	1.0 molec$^{-1}s^{-1}$
MEK(mapk=1, phospho=’p’, ppp2=None) % ERK(map2k=1, phospho=’u’) >> MEK(mapk=None, phospho=’p’, ppp2=None) + ERK(map2k=None, phospho=’p’)	10.0 s^{-1}
MEK(mapk=1) % ERK(map2k=1) >> MEK(mapk=None) + ERK(map2k=None)	0.1 s^{-1}
DUSP6(mapk=None) + ERK(phospho=’p’, sos=None, dusp=None) >> DUSP6(mapk=1) % ERK(phospho=’p’, sos=None, dusp=1)	1.0 molec$^{-1}s^{-1}$
DUSP6(mapk=1) % ERK(phospho=’p’, sos=None, dusp=1) >> DUSP6(mapk=None) + ERK(phospho=’u’, sos=None, dusp=None)	10.0 s^{-1}
DUSP6(mapk=1) % ERK(dusp=1) >> DUSP6(mapk=None) + ERK(dusp=None)	0.001 s^{-1}
BRAF(V600=’E’, vemurafenib=None, map2k=None) + MEK(phospho=’u’, map3k=None) >> BRAF(V600=’E’, vemurafenib=None, map2k=1) % MEK(phospho=’u’, map3k=1)	1.0 molec$^{-1}s^{-1}$
BRAF(V600=’E’, vemurafenib=None, map2k=1) % MEK(phospho=’u’, map3k=1) >> BRAF(V600=’E’, vemurafenib=None, map2k=None) + MEK(phospho=’p’, map3k=None)	3.0 s^{-1}
BRAF(V600=’E’, map2k=1) % MEK(map3k=1) >> BRAF(V600=’E’, map2k=None) + MEK(map3k=None)	0.1 s^{-1}

Table S5. Rules and parameters for MEMI1.0

Rule	Forward rate
EGF(erbb=None) + EGFR(egfr_ligand=None) >> EGF(erbb=1) % EGFR(egfr_ligand=1)	1.0 molec$^{-1}s^{-1}$
EGF(erbb=1) % EGFR(egfr_ligand=1) >> EGF(erbb=None) + EGFR(egfr_ligand=None)	0.1 s^{-1}
EGFR(egfr_ligand=ANY, erbb=None) + EGFR(egfr_ligand=ANY, erbb=None) >> EGFR(egfr_ligand=ANY, erbb=1) % EGFR(egfr_ligand=ANY, erbb=1)	1.0 molec$^{-1}s^{-1}$
EGFR(erbb=1) % EGFR(erbb=1) >> EGFR(erbb=None) + EGFR(erbb=None)	0.1 s^{-1}
EGFR(erbb=ANY, grb2=None) + GRB2(erbb=None) >> EGFR(erbb=ANY, grb2=1) % GRB2(erbb=1)	1.0 molec$^{-1}s^{-1}$
EGFR(grb2=1) % GRB2(erbb=1) >> EGFR(grb2=None) + GRB2(erbb=None)	0.1 s^{-1}
RAS(sos=ANY, gtp=None) + GTP(ras=None) >> RAS(sos=ANY, gtp=1) % GTP(ras=1)	50.0 molec$^{-1}s^{-1}$
RAS(gtp=1) % GTP(ras=1) >> RAS(gtp=None) + GTP(ras=None)	0.5 s^{-1}
RAS(sos=None, gtp=ANY, map3k=None) + BRAF(V600='E', ras=None) >> RAS(sos=None, gtp=ANY, map3k=1) % BRAF(V600='E', ras=1)	1.0 molec$^{-1}s^{-1}$
RAS(map3k=1) % BRAF(V600='E', ras=1) >> RAS(map3k=None) + BRAF(V600='E', ras=None)	0.5 s^{-1}
VEMURAFENIB(map3k=None) + BRAF(V600='E', vemurafenib=None) >> VEMURAFENIB(map3k=1) % BRAF(V600='E', vemurafenib=1)	10.0 molec$^{-1}s^{-1}$
VEMURAFENIB(map3k=1) % BRAF(V600='E', vemurafenib=1) >> VEMURAFENIB(map3k=None) + BRAF(V600='E', vemurafenib=None)	1.0 s^{-1}
GRB2(erbb=ANY, sos=None) + SOS(S=(u'u', WILD), grb2=None) >> GRB2(erbb=ANY, sos=1) % SOS(S=(u'u', WILD), grb2=1)	1.0 molec$^{-1}s^{-1}$
GRB2(sos=1) % SOS(grb2=1) >> GRB2(sos=None) + SOS(grb2=None)	0.1 s^{-1}
SOS(ras=None, S=(u'u', WILD), grb2=ANY) + RAS(sos=None, map3k=None) >> SOS(ras=1, S=(u'u', WILD), grb2=ANY) % RAS(sos=1, map3k=None)	1.0 molec$^{-1}s^{-1}$
SOS(ras=1) % RAS(sos=1) >> SOS(ras=None) + RAS(sos=None)	50.0 s^{-1}
PPP2CA(map2k=None) + MEK(mapk=None, phospho='p', ppp2=None) >> PPP2CA(map2k=1) % MEK(mapk=None, phospho='p', ppp2=1)	1.0 molec$^{-1}s^{-1}$
PPP2CA(map2k=1) % MEK(mapk=None, phospho='p', ppp2=1) >> PPP2CA(map2k=None) + MEK(mapk=None, phospho='u', ppp2=None)	10.0 s^{-1}
PPP2CA(map2k=1) % MEK(ppp2=1) >> PPP2CA(map2k=None) + MEK(ppp2=None)	0.001 s^{-1}
MEK(mapk=None, phospho='p', ppp2=None) + ERK(map2k=None, phospho='u') >> MEK(mapk=1, phospho='p', ppp2=None) % ERK(map2k=1, phospho='u')	1.0 molec$^{-1}s^{-1}$
MEK(mapk=1, phospho='p', ppp2=None) % ERK(map2k=1, phospho='u') >> MEK(mapk=None, phospho='p', ppp2=None) + ERK(map2k=None, phospho='p')	10.0 s^{-1}
MEK(mapk=1) % ERK(map2k=1) >> MEK(mapk=None) + ERK(map2k=None)	0.1 s^{-1}
DUSP6(mapk=None) + ERK(phospho='p', sos=None, dusp=None) >> DUSP6(mapk=1) % ERK(phospho='p', sos=None, dusp=1)	1.0 molec$^{-1}s^{-1}$
DUSP6(mapk=1) % ERK(phospho='p', sos=None, dusp=1) >> DUSP6(mapk=None) + ERK(phospho='u', sos=None, dusp=None)	10.0 s^{-1}
DUSP6(mapk=1) % ERK(dusp=1) >> DUSP6(mapk=None) + ERK(dusp=None)	0.001 s^{-1}
BRAF(V600='E', vemurafenib=None, map2k=None) + MEK(phospho='u', map3k=None) >> BRAF(V600='E', vemurafenib=None, map2k=1) % MEK(phospho='u', map3k=1)	1.0 molec$^{-1}s^{-1}$
BRAF(V600='E', vemurafenib=None, map2k=1) % MEK(phospho='u', map3k=1) >> BRAF(V600='E', vemurafenib=None, map2k=None) + MEK(phospho='p', map3k=None)	3.0 s^{-1}
BRAF(V600='E', map2k=1) % MEK(map3k=1) >> BRAF(V600='E', map2k=None) + MEK(map3k=None)	0.1 s^{-1}
ERK(phospho='p', sos=None, dusp=None) + SOS(ras=None, S='u', mapk=None) >> ERK(phospho='p', sos=1, dusp=None) % SOS(ras=None, S='u', mapk=1)	10^{-5} molec$^{-1}s^{-1}$
ERK(phospho='p', sos=1, dusp=None) % SOS(ras=None, S='u', mapk=1) >> ERK(phospho='p', sos=None, dusp=None) + SOS(ras=None, S='p', mapk=None)	1.0 s^{-1}
ERK(sos=1) % SOS(mapk=1) >> ERK(sos=None) + SOS(mapk=None)	0.0001 s^{-1}
PHOSPHATASE(sos=None) + SOS(S='p', phosphatase=None) >> PHOSPHATASE(sos=1) % SOS(S='p', phosphatase=1)	1.0 molec$^{-1}s^{-1}$
PHOSPHATASE(sos=1) % SOS(S='p', phosphatase=1) >> PHOSPHATASE(sos=None) + SOS(S='u', phosphatase=None)	0.0001 s^{-1}
PHOSPHATASE(sos=1) % SOS(phosphatase=1) >> PHOSPHATASE(sos=None) + SOS(phosphatase=None)	0.1 s^{-1}

Table S6. Rules and parameters for MEMI1.1

Rule	Forward rate
EGF(erbb=None) + EGFR(egfr_ligand=None) >> EGF(erbb=1) % EGFR(egfr_ligand=1)	1.0 molec$^{-1}s^{-1}$
EGF(erbb=1) % EGFR(egfr_ligand=1) >> EGF(erbb=None) + EGFR(egfr_ligand=None)	0.1 s^{-1}
EGFR(egfr_ligand=ANY, erbb=None) + EGFR(egfr_ligand=ANY, erbb=None) >> EGFR(egfr_ligand=ANY, erbb=1) % EGFR(egfr_ligand=ANY, erbb=1)	1.0 molec$^{-1}s^{-1}$
EGFR(erbb=1) % EGFR(erbb=1) >> EGFR(erbb=None) + EGFR(erbb=None)	0.1 s^{-1}
EGFR(erbb=ANY, grb2=None) + GRB2(erbb=None) >> EGFR(erbb=ANY, grb2=1) % GRB2(erbb=1)	1.0 molec$^{-1}s^{-1}$
EGFR(grb2=1) % GRB2(erbb=1) >> EGFR(grb2=None) + GRB2(erbb=None)	0.1 s^{-1}
RAS(sos=ANY, gtp=None) + GTP(ras=None) >> RAS(sos=ANY, gtp=1) % GTP(ras=1)	50.0 molec$^{-1}s^{-1}$
RAS(gtp=1) % GTP(ras=1) >> RAS(gtp=None) + GTP(ras=None)	0.5 s^{-1}
RAS(sos=None, gtp=ANY, map3k=None) + BRAF(V600=’E’, ras=None) >> RAS(sos=None, gtp=ANY, map3k=1) % BRAF(V600=’E’, ras=1)	1.0 molec$^{-1}s^{-1}$
RAS(map3k=1) % BRAF(V600=’E’, ras=1) >> RAS(map3k=None) + BRAF(V600=’E’, ras=None)	0.5 s^{-1}
GRB2(erbb=ANY, sos=None) + SOS(S=(u’u’, WILD), grb2=None) >> GRB2(erbb=ANY, sos=1) % SOS(S=(u’u’, WILD), grb2=1)	1.0 molec$^{-1}s^{-1}$
GRB2(sos=1) % SOS(grb2=1) >> GRB2(sos=None) + SOS(grb2=None)	0.1 s^{-1}
SOS(ras=None, S=(u’u’, WILD), grb2=ANY) + RAS(sos=None, map3k=None) >> SOS(ras=1, S=(u’u’, WILD), grb2=ANY) % RAS(sos=1, map3k=None)	1.0 molec$^{-1}s^{-1}$
SOS(ras=1) % RAS(sos=1) >> SOS(ras=None) + RAS(sos=None)	50.0 s^{-1}
BRAF(V600=’E’, ras=ANY, map3k=None) + BRAF(V600=’E’, ras=ANY, map3k=None) >> BRAF(V600=’E’, ras=ANY, map3k=1) % BRAF(V600=’E’, ras=ANY, map3k=1)	10.0 molec$^{-1}s^{-1}$
BRAF(V600=’E’, map3k=1) % BRAF(V600=’E’, map3k=1) >> BRAF(V600=’E’, map3k=None) + BRAF(V600=’E’, map3k=None)	1.0 s^{-1}
VEMURAFENIB(map3k=None) + BRAF(V600=’E’, map3k=None, vemurafenib=None) >> VEMURAFENIB(map3k=1) % BRAF(V600=’E’, map3k=None, vemurafenib=1)	10.0 molec$^{-1}s^{-1}$
VEMURAFENIB(map3k=1) % BRAF(V600=’E’, vemurafenib=1) >> VEMURAFENIB(map3k=None) + BRAF(V600=’E’, vemurafenib=None)	1.0 s^{-1}
VEMURAFENIB(map3k=None) + BRAF(V600=’E’, map3k=ANY, vemurafenib=None) >> VEMURAFENIB(map3k=1) % BRAF(V600=’E’, map3k=ANY, vemurafenib=1)	0.0001 molec$^{-1}s^{-1}$
PPP2CA(map2k=None) + MEK(mapk=None, phospho=’p’, ppp2=None) >> PPP2CA(map2k=1) % MEK(mapk=None, phospho=’p’, ppp2=1)	1.0 molec$^{-1}s^{-1}$
PPP2CA(map2k=1) % MEK(mapk=None, phospho=’p’, ppp2=1) >> PPP2CA(map2k=None) + MEK(mapk=None, phospho=’u’, ppp2=None)	10.0 s^{-1}
PPP2CA(map2k=1) % MEK(ppp2=1) >> PPP2CA(map2k=None) + MEK(ppp2=None)	0.001
MEK(mapk=None, phospho=’p’, ppp2=None) + ERK(map2k=None, phospho=’u’) >> MEK(mapk=1, phospho=’p’, ppp2=None) % ERK(map2k=1, phospho=’u’)	1.0 s^{-1}
MEK(mapk=1, phospho=’p’, ppp2=None) % ERK(map2k=1, phospho=’u’) >> MEK(mapk=None, phospho=’p’, ppp2=None) + ERK(map2k=None, phospho=’p’)	10.0 molec$^{-1}s^{-1}$
MEK(mapk=1) % ERK(map2k=1) >> MEK(mapk=None) + ERK(map2k=None)	0.1 s^{-1}
DUSP6(mapk=None) + ERK(phospho=’p’, sos=None, dusp=None) >> DUSP6(mapk=1) % ERK(phospho=’p’, sos=None, dusp=1)	1.0 molec$^{-1}s^{-1}$
DUSP6(mapk=1) % ERK(phospho=’p’, sos=None, dusp=1) >> DUSP6(mapk=None) + ERK(phospho=’u’, sos=None, dusp=None)	10.0 s^{-1}
DUSP6(mapk=1) % ERK(dusp=1) >> DUSP6(mapk=None) + ERK(dusp=None)	0.001 s^{-1}
BRAF(V600=’E’, vemurafenib=None, map2k=None) + MEK(phospho=’u’, map3k=None) >> BRAF(V600=’E’, vemurafenib=None, map2k=1) % MEK(phospho=’u’, map3k=1)	1.0 molec$^{-1}s^{-1}$
BRAF(V600=’E’, vemurafenib=None, map2k=1) % MEK(phospho=’u’, map3k=1) >> BRAF(V600=’E’, vemurafenib=None, map2k=None) + MEK(phospho=’p’, map3k=None)	3.0 s^{-1}
BRAF(V600=’E’, map2k=1) % MEK(map3k=1) >> BRAF(V600=’E’, map2k=None) + MEK(map3k=None)	0.1 s^{-1}
ERK(phospho=’p’, sos=None, dusp=None) + SOS(ras=None, S=’u’, mapk=None) >> ERK(phospho=’p’, sos=1, dusp=None) % SOS(ras=None, S=’u’, mapk=1)	10^{-5} molec$^{-1}s^{-1}$
ERK(phospho=’p’, sos=1, dusp=None) % SOS(ras=None, S=’u’, mapk=1) >> ERK(phospho=’p’, sos=None, dusp=None) + SOS(ras=None, S=’p’, mapk=None)	1.0 s^{-1}
ERK(sos=1) % SOS(mapk=1) >> ERK(sos=None) + SOS(mapk=None)	0.0001 s^{-1}
PHOSPHATASE(sos=None) + SOS(S=’p’, phosphatase=None) >> PHOSPHATASE(sos=1) % SOS(S=’p’, phosphatase=1)	1.0 molec$^{-1}s^{-1}$
PHOSPHATASE(sos=1) % SOS(S=’p’, phosphatase=1) >> PHOSPHATASE(sos=None) + SOS(S=’u’, phosphatase=None)	0.0001 s^{-1}
PHOSPHATASE(sos=1) % SOS(phosphatase=1) >> PHOSPHATASE(sos=None) + SOS(phosphatase=None)	0.1 s^{-1}

Table S7. Rules and parameters for MEMI 1.2

Monomer	Copy number
EGF	10^3
EGFR	10^5
GRB2	10^5
SOS	10^3
GTP	10^7
RAS	$2 \cdot 10^5$
BRAF-V600E	10^5
MEK	10^5
ERK	10^5
PPP2CA	10^5
DUSP6	10^3
Phosphatase	10^2

Table S8. Total amounts of molecular species (monomers) for MEMI1.0-1.2 shown as copy numbers per cell

A Pathway Commons query was performed with INDRA to obtain paths from MAPK1 or MAPK3 to SOS1 or SOS2. This yielded the following list of INDRA Statements

- Phosphorylation(MAPK1(), SOS1(), S, 1132),
- Phosphorylation(MAPK1(), SOS1(), S, 1197),
- Phosphorylation(MAPK1(), SOS1(), S, 1193),
- Phosphorylation(MAPK1(), SOS1(), S, 1178)

This indicates that MAPK1 phosphorylates SOS1 on several serine residues.

We queried the BEL Large Corpus using a neighborhood search around SOS1 and SOS2. This returned the following relevant mechanisms:

- Phosphorylation(MAPK1(), SOS1(), S, 1178),
- Phosphorylation(MAPK1(), SOS1(), S, 1167),
- Inhibition(MAPK1(activity='kinase'), SOS1(), 'catalytic')

All three mechanisms refer to PMID8816480, *Identification of the mitogen-activated protein kinase phosphorylation sites on human Sos1 that regulate interaction with Grb2* (Corbalan-Garcia *et al*, 1996) as their source evidence. This implies that the serine-phosphorylation of SOS1 by MAPK1 reduces its affinity to bind to GRB2 thereby resulting in its loss of activity.

2.5 An extensible and executable map of the RAS pathway

The following text was used to assemble the RAS pathway map.

Growth-factor proteins activate EGFR, ERBB2 and FGFR.
Growth-factor proteins activate PDGFR.
Growth-factor proteins activate MET, ROS1 and ALK.
EGFR, ERBB2, PDGFR, MET, ROS1, ALK and FGFR activate GRB2 and SHC.
GRB2 and SHC activate RASGRF and SOS.
GRB2 binds SHC.
SOS and RASGRF activate HRAS, NRAS and KRAS.
RASGRP activates HRAS, KRAS and NRAS.
SPRY deactivates HRAS, KRAS and NRAS.
The RASA-ARHGAP35 complex deactivates HRAS, NRAS and KRAS.
RASAL deactivates HRAS, NRAS and KRAS.
The SPRED-NF1 complex deactivates HRAS, NRAS and KRAS.
The RASA-ARHGAP35 complex deactivates RHOA, RHOB and RHOC.
RASAL deactivates RHOA, RHOB and RHOC.
The SPRED-NF1 complex deactivates RHOA, RHOB and RHOC.
HRAS, NRAS and KRAS activate RALGDS.
RALGDS activates RALA and RALB.
HRAS, NRAS and KRAS activate ARAF, BRAF and RAF1.
ARAF, BRAF and RAF1 activate MAP2K1 and MAP2K2.
MAP2K1 and MAP2K2 activate MAPK1 and MAPK3.
MAPK1 and MAPK3 activate ETS, JUN and FOS.
KSR binds ARAF, BRAF and RAF1.
KSR binds MAP2K1 and MAP2K2.
KSR binds MAPK1 and MAPK3.
ETS, FOS and JUN activate MDM2, CCND1 and DUSP.
MDM2 deactivates TP53.
CCND1 activates CDK4 and CDK6.
CDK4 and CDK6 deactivate pRB.
DUSP deactivates MAPK1 and MAPK3.
SOS and RASGRF activate RHOA and RHOB.
SOS and RASGRF activate RHOC.
RHOA activates ROCK1 and ROCK2.
RHOB and RHOC activate ROCK1 and ROCK2.
HRAS, NRAS and KRAS activate PI3K.
PI3K activates PIP3.
PTEN deactivates PIP3.
PIP3 activates PDPK1, AKT and TIAM.
PDPK1 activates AKT.
AKT deactivates TSC1 and TSC2.
TSC1 and TSC2 deactivate RHEB.
RHEB activates mTORC2.
STK11 activates AMPK.
AMPK deactivates mTORC2.
mTORC2 deactivates EIF4EBP1.
mTORC2 activates P90RSK.
TIAM activates RAC and RAC activates PAK.

Below is the set of logical functions that define the update steps of the Boolean network automatically assembled from the RAS pathway map.

```
mTORC2* = (RHEB) and not (AMPK)
EGFR* = GROWTH-FACTOR
TIAM* = PIP-3
RHOC* = (RASGRF or SOS) and not (RASAL or SPRED or RASA)
ARAF* = KRAS or HRAS or NRAS
AKT* = PDPK1 or PIP-3
EIF4EBP1* = not (mTORC2)
SHC* = EGFR or PDGFR or ALK or ERBB2 or MET or FGFR or ROS1
JUN* = MAPK1 or MAPK3
ROCK2* = RHOB or RHOC or RHOA
TP53* = not (MDM2)
CCND1* = ETS or JUN or FOS
RHOA* = (RASGRF or SOS) and not (RASAL or SPRED or RASA)
CDK4* = CCND1
TSC2* = not (AKT)
RAC* = TIAM
MAP2K2* = RAF1 or BRAF or ARAF
BRAF* = KRAS or HRAS or NRAS
PAK* = RAC
ROCK1* = RHOB or RHOC or RHOA
GRB2* = EGFR or PDGFR or ALK or ERBB2 or MET or FGFR or ROS1
AMPK* = STK11
ROS1* = GROWTH-FACTOR
RHEB* = not (TSC2 or TSC1)
MAP2K1* = RAF1 or BRAF or ARAF
MDM2* = ETS or JUN or FOS
RB1* = not (CDK4 or CDK6)
HRAS* = (RASGRF or RASGRP or SOS) and not (RASA or SPRY or RASAL or SPRED)
MAPK3* = (MAP2K1 or MAP2K2) and not (DUSP)
PI3K* = KRAS or HRAS or NRAS
MET* = GROWTH-FACTOR
FGFR* = GROWTH-FACTOR
RAF1* = KRAS or HRAS or NRAS
ETS* = MAPK1 or MAPK3
RALA* = RALGDS
PIP-3* = (PI3K) and not (PTEN)
RALB* = RALGDS
SOS* = SHC or GRB2
KRAS* = (RASGRF or RASGRP or SOS) and not (RASA or SPRY or RASAL or SPRED)
NRAS* = (RASGRF or RASGRP or SOS) and not (RASA or SPRY or RASAL or SPRED)
RASGRF* = SHC or GRB2
ALK* = GROWTH-FACTOR
ERBB2* = GROWTH-FACTOR
RHOB* = (RASGRF or SOS) and not (RASAL or SPRED or RASA)
PDGFR* = GROWTH-FACTOR
MAPK1* = (MAP2K1 or MAP2K2) and not (DUSP)
TSC1* = not (AKT)
DUSP* = ETS or JUN or FOS
CDK6* = CCND1
RALGDS* = KRAS or HRAS or NRAS
PDPK1* = PIP-3
P90RSK* = mTORC2
FOS* = MAPK1 or MAPK3
```

Boolean network simulations were performed using the *boolean2* package available from https://github.com/ialbert/booleannet (Albert *et al*, 2008). We simulated 100 traces using asynchronous updates on the nodes (which results in stochastic behavior) and then took the average of the value of each node (with 0 corresponding to the low and 1 to the high state of each node).

References

Albert I, Thakar J, Li S, Zhang R, Albert R (2008) Boolean network simulations for life scientists. *Source Code Biol Med* 3: 1–8

Allen J, Ferguson G, Miller B, Ringger E (1995) TRAINS as an embodied natural language dialogue system. In *AAAI Technical Report FS-95-05*

Allen J, Swift M, de Beaumont W (2008) Deep semantic analysis of text. In *2008 ACL Conference on Semantics in Text Processing* 343–354

Allen J, de Beaumont W, Galescu L, Teng CM (2015) Complex Event Extraction using DRUM. In *2015 ACL-IJCNLP*, 1–11

Babur O, Dogrusoz U, Demir E, Sander C (2010) ChiBE: interactive visualization and manipulation of BioPAX pathway models. *Bioinformatics* 26: 429–431

Corbalan-Garcia S, Yang S, Degenhardt K, Bar-Sagi D (1996) Identification of the mitogen-activated protein kinase phosphorylation sites on human Sos1 that regulate interaction with Grb2. *Mol Cell Biol* 16: 5674–5682

Fellbaum C, ed (1998) *WordNet: An electronic lexical database.* MIT Press, Cambridge, MA, USA

Hara T, Miyao Y, Tsujii J (2005) Adapting a probabilistic disambiguation model of an HPSG parser to a new domain. In *2005 ACL-IJCNLP* 199–210

Manning CD, Surdeanu M, Bauer J, Finkel JR, Bethard S, McClosky D (2014) The Stanford CoreNLP natural language processing toolkit. In *2014 ACL System Demonstrations* 55–60

Manshadi MH, Allen J, Swift M (2008) In *13th Conference on Formal Grammar* 77–94.

Sari M, Bahceci I, Dogrusoz U, Sumer SO, Aksoy BA, Babur O, Demir E (2015) SBGNViz: a tool for visualization and complexity management of SBGN process description maps. *PloS One* 10: e0128985

Appendix Notebook 1: Inspecting INDRA Statements and assembled models

In this example we look at how intermediate results of the assembly process from word models to executable models can be inspected. We first import the necessary modules of INDRA.

In [1]:
```
%pylab inline
import json
from indra.sources import trips
from indra.statements import draw_stmt_graph, stmts_to_json
```

```
Populating the interactive namespace from numpy and matplotlib
```

Collecting Statements from reading

First, we use the TRIPS system via INDRA's `trips` module to read two sentences which describe distinct mechanistic hypotheses about ATM phosphorylation.

In [2]:
```
text = 'Active ATM phosphorylates itself. Active ATM phosphorylates another ATM molecul
e.'
tp = trips.process_text(text)
```

Here `tp` is a `TripsProcessor` object whose extracted Statements can be accessed in a list.

Printing Statements as objects

It is possible to look at the string representation of the extracted INDRA Statements as below.

In [3]:
```
tp.statements
```

Out[3]:
```
[Autophosphorylation(ATM(activity: True)),
 Phosphorylation(ATM(activity: True), ATM())]
```

The first Statement, obtained by reading "Active ATM phosphorylates itself", represents the Autophosphorylation of ATM with ATM being in an active state. Here `activity` stands for generic molecular activity and `True` indicates an active as opposed to an inactive state.

The second Statement, obtained from "Active ATM phosphorylates another ATM molecule" is a Phosphorylation with the enzyme ATM being in an active state phosphorylating another ATM as a substrate.

Drawing Statements as graphs

Next, we can use the `draw_stmt_graph` function to display the Statements produced by reading and INDRA input processing as a graph. The root of each tree is the type of the Statement, in this case Autophosphorylation. The arguments of the Statement branch off from the root. In this case the enzyme argument of Autophosphorylation is an Agent with name ATM. Its database references can be inspected under the `db_refs` property.

In [4]:
```
pylab.rcParams['figure.figsize'] = (12, 8)
draw_stmt_graph(tp.statements[1:])
```

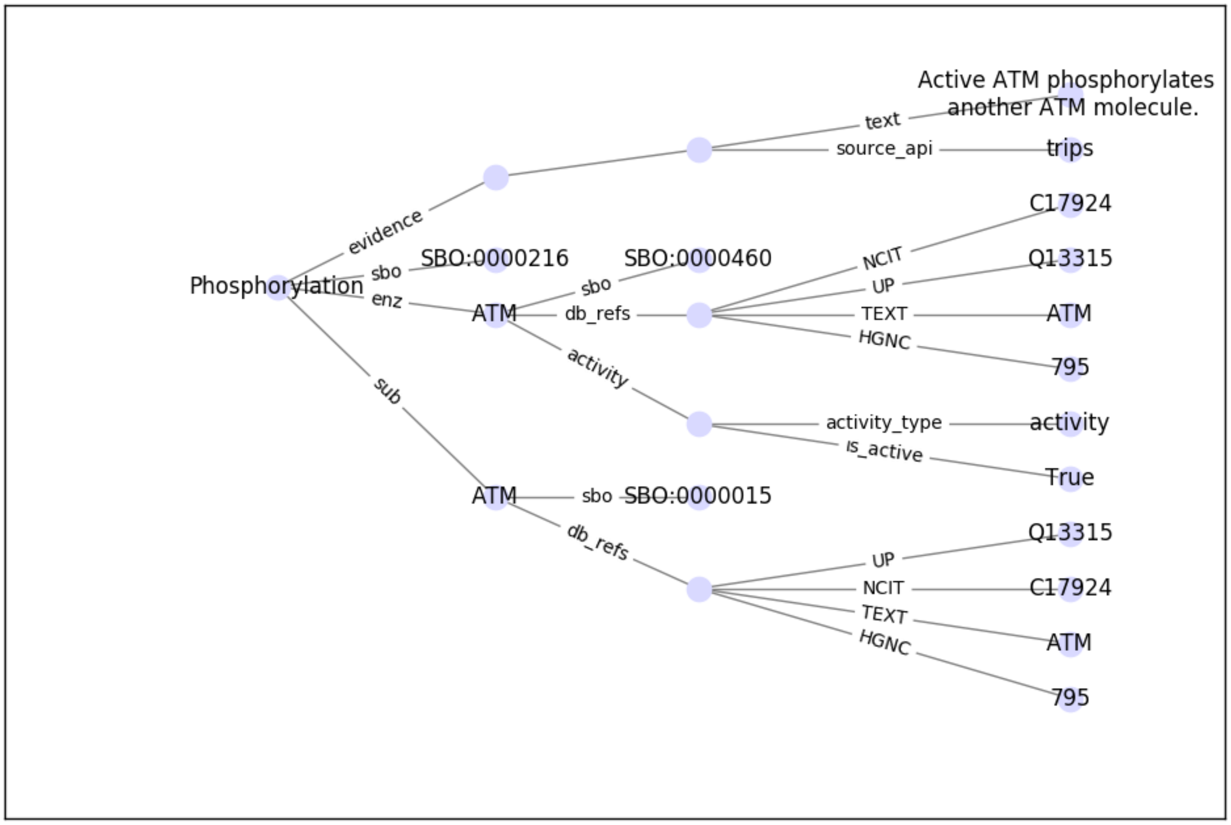

Printing / exchanging Statements as JSON

INDRA Statements can be serialized into JSON format. This is a human-readable and editable form of INDRA Statements which is independent of Python and can therefore be used as a platform-independent data exchange format for Statements. The function `stmts_to_json` in the `indra.statements` module takes a list of Statements and returns a JSON as a dictionary. Below we pretty-print this JSON as a string with indentations.

In [5]:
```
statements_json = stmts_to_json(tp.statements)
print(json.dumps(statements_json, indent=1))
```


```
[
 {
  "type": "Autophosphorylation",
  "enz": {
   "name": "ATM",
   "activity": {
    "activity_type": "activity",
    "is_active": true
   },
   "db_refs": {
    "TEXT": "ATM",
    "HGNC": "795",
    "UP": "Q13315",
    "NCIT": "C17924"
   },
   "sbo": "http://identifiers.org/sbo/SBO:0000460"
  },
  "evidence": [
   {
    "source_api": "trips",
    "text": "Active ATM phosphorylates itself."
   }
  ],
  "id": "35362b6c-2229-436a-862f-fec499713a9d",
  "sbo": "http://identifiers.org/sbo/SBO:0000216"
 },
 {
  "type": "Phosphorylation",
  "enz": {
   "name": "ATM",
   "activity": {
    "activity_type": "activity",
    "is_active": true
   },
   "db_refs": {
    "TEXT": "ATM",
    "HGNC": "795",
    "UP": "Q13315",
    "NCIT": "C17924"
   },
   "sbo": "http://identifiers.org/sbo/SBO:0000460"
  },
  "sub": {
   "name": "ATM",
   "db_refs": {
    "TEXT": "ATM",
    "HGNC": "795",
    "UP": "Q13315",
    "NCIT": "C17924"
   },
   "sbo": "http://identifiers.org/sbo/SBO:0000015"
  },
  "evidence": [
   {
    "source_api": "trips",
    "text": "Active ATM phosphorylates another ATM molecule."
   }
  ],
  "id": "cb1fae70-41b8-4be0-b1d2-aa8ebcbb20a3",
  "sbo": "http://identifiers.org/sbo/SBO:0000216"
 }
]
```

Inspecting assembled rule-based models

We now assemble two PySB models, one for each Statement.

In [6]:
```
from indra.assemblers import pysb_assembler
pa = pysb_assembler.PysbAssembler()
pa.add_statements([tp.statements[0]])
model1 = pa.make_model()
```

We can examine the properties of the PySB model object before exporting it. As seen below, the model has a single Monomer and Rule, and two Parameters.

In [7]:
```
model1
```

Out[7]:
```
<Model 'None' (monomers: 1, rules: 1, parameters: 2, expressions: 0, compartments: 0) a
t 0x109364240>
```

We can look at the ATM Monomer and its sites. ATM has an `activity` site which can be either `active` of `inactive`. It also has a `phospho` site with `u` and `p` states.

In [8]:
```
model1.monomers['ATM']
```

Out[8]:
```
Monomer('ATM', ['activity', 'phospho'], {'activity': ['inactive', 'active'], 'phospho':
['u', 'p']})
```

The rule representing ATM autophosphorylation can be inspected below. The rule is parameterized by the forward rate `kf_a_autophos_1`.

In [9]:
```
model1.rules[0]
```

Out[9]:
```
Rule('ATM_autophospho_ATM_phospho', ATM(activity='active', phospho='u') >> ATM(activity
='active', phospho='p'), kf_a_autophos_1)
```

We now assemble a model for the second Statement.

In [10]:
```
pa = pysb_assembler.PysbAssembler()
pa.add_statements([tp.statements[1]])
model2 = pa.make_model()
```

In [11]:
```
model2
```

Out[11]:
```
<Model 'None' (monomers: 1, rules: 1, parameters: 2, expressions: 0, compartments: 0) a
t 0x1216ab358>
```

In [12]:
```
model2.monomers['ATM']
```

Out[12]:
```
Monomer('ATM', ['activity', 'phospho'], {'activity': ['inactive', 'active'], 'phospho':
['u', 'p']})
```

In [13]:
```
model2.rules[0]
```

Out[13]:
```
Rule('ATM_phosphorylation_ATM_phospho', ATM(activity='active') + ATM(phospho='u') >> AT
M(activity='active') + ATM(phospho='p'), kf_aa_phosphorylation_1)
```

As we see, the rule assembled for this model contains two distinct ATMs on each side, one acting as the kinase and the other as the substrate.

Inspecting assembled model annotations

Finally, models assembled by INDRA carry automatically propagated annotations. Below, the grounding of ATM in the UniProt, HGNC and NCIT databases is annotated; the semantic role of monomers in each rule are also annotated, and finally, the unique ID of the INDRA Statement that a rule was derived from is annotated.

In [14]:
```
model1.annotations
```

Out[14]:
```
[Annotation(ATM, 'http://identifiers.org/hgnc/HGNC:795', 'is'),
 Annotation(ATM, 'http://identifiers.org/uniprot/Q13315', 'is'),
 Annotation(ATM, 'http://identifiers.org/ncit/C17924', 'is'),
 Annotation(ATM_autophospho_ATM_phospho, 'ATM', 'rule_has_subject'),
 Annotation(ATM_autophospho_ATM_phospho, 'ATM', 'rule_has_object'),
 Annotation(ATM_autophospho_ATM_phospho, '35362b6c-2229-436a-862f-fec499713a9d', 'from_
indra_statement')]
```

In [15]:
```
model2.annotations
```

Out[15]:
```
[Annotation(ATM, 'http://identifiers.org/hgnc/HGNC:795', 'is'),
 Annotation(ATM, 'http://identifiers.org/uniprot/Q13315', 'is'),
 Annotation(ATM, 'http://identifiers.org/ncit/C17924', 'is'),
 Annotation(ATM_phosphorylation_ATM_phospho, 'ATM', 'rule_has_subject'),
 Annotation(ATM_phosphorylation_ATM_phospho, 'ATM', 'rule_has_object'),
 Annotation(ATM_phosphorylation_ATM_phospho, 'cb1fae70-41b8-4be0-b1d2-aa8ebcbb20a3', 'f
rom_indra_statement')]
```

Appendix Notebook 2: Model complexity, context specification and assembly policies

In this notebook we explore the effects of specified conditions on Agents (e.g. bound conditions, modification conditions) and assembly policies on the combinatorial complexity of dynamical models.

First, we import INDRA's TRIPS input API and PySB model assembler.

In [1]:
```
from indra.sources import trips
from indra.assemblers import PysbAssembler

# Below is some bookkeeping code needed to display reaction network graphs
%matplotlib inline
from pysb.tools import render_reactions
import pygraphviz, subprocess
import matplotlib.image as mpimg
import matplotlib.pyplot as plt
def draw_reaction_network(model):
    pygraphviz.AGraph(render_reactions.run(model)).draw('model.png', prog='dot')
    img = mpimg.imread('model.png')
    plt.figure(figsize=(50, 50))
    plt.imshow(img)
    plt.xticks([])
    plt.yticks([])
```

Model1: RAF to ERK without specifying agent context

In the first case, two binding events and a phosphorylation is described with no additional context specified on any of the proteins.

In [2]:
```
tp = trips.process_text('RAF binds Vemurafenib, RAF phosphorylates MEK and MEK phosphory
lates ERK.')
```

This yields 3 INDRA Statements, as follows. Here empty parentheses after the Agent names indicate that there is no additional context specified on them.

In [3]:
```
tp.statements
```

Out[3]:
```
[Complex(RAF(), VEMURAFENIB()),
 Phosphorylation(RAF(), MEK()),
 Phosphorylation(MEK(), ERK())]
```

Assembly with one-step policy

We now assemble this model using the default `one_step` policy and store it in the `model1_one` variable.

In [4]:
```
pa = PysbAssembler()
pa.add_statements(tp.statements)
pa.make_model(policies='one_step')
```

Out[4]:
```
<Model 'None' (monomers: 4, rules: 4, parameters: 8, expressions: 0, compartments: 0) a
t 0x1216fa5f8>
```

In [5]:
```
model1_one = pa.model
```

The model has 4 Monomers and 4 Rules.

In [6]:
```
model1_one.monomers
```

Out[6]:
```
ComponentSet([
 Monomer('MEK', ['phospho'], {'phospho': ['u', 'p']}),
 Monomer('ERK', ['phospho'], {'phospho': ['u', 'p']}),
 Monomer('RAF', ['vemurafenib']),
 Monomer('VEMURAFENIB', ['map3k']),
 ])
```

In [7]:
```
model1_one.rules
```

Out[7]:
```
ComponentSet([
 Rule('RAF_VEMURAFENIB_bind', RAF(vemurafenib=None) + VEMURAFENIB(map3k=None) >> RAF(ve
murafenib=1) % VEMURAFENIB(map3k=1), kf_rv_bind_1),
 Rule('RAF_VEMURAFENIB_dissociate', RAF(vemurafenib=1) % VEMURAFENIB(map3k=1) >> RAF(ve
murafenib=None) + VEMURAFENIB(map3k=None), kr_rv_bind_1),
 Rule('RAF_phosphorylation_MEK_phospho', RAF() + MEK(phospho='u') >> RAF() + MEK(phosph
o='p'), kf_rm_phosphorylation_1),
 Rule('MEK_phosphorylation_ERK_phospho', MEK() + ERK(phospho='u') >> MEK() + ERK(phosph
o='p'), kf_me_phosphorylation_1),
 ])
```

Let's examine the last rule which corresponds to MEK phosphorylating ERK. Here, `MEK()` appears without additional context specified. This means that the rule will apply to **any** form of `MEK`, for instance, MEK that is unphosphorylated.

We now generate the rule-based model into a reaction network form using PySB's interface to BioNetGen.

In [8]:
```
from pysb.bng import generate_equations
generate_equations(model1_one)
```

We can now plot the reaction network to examine the model. Each colored node of the reaction network is a molecular species, reactions are represented by gray nodes, and arrows show species being consumed and produced by each reaction.

In [9]:
```
draw_reaction_network(model1_one)
```

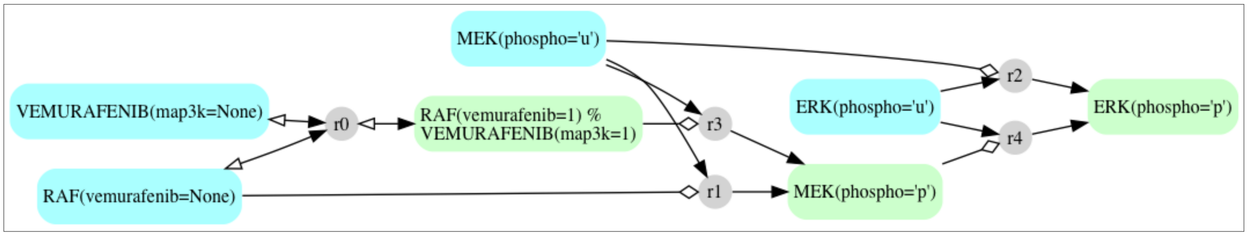

We see from the reaction network that RAF is able to phosphorylate MEK whether or not it is bound to Vemurafenib, and MEK phosphorylates ERK whether or not it is phosphorylated.

Assembly with two-step policy

Let's now assemble the same model with the `two-step` policy. This will result in a more detailed model in which MEK first binds ERK reversibly, and phosphorylated ERK is released from the MEK-ERK complex. We will store this model in the `model1_two` variable.

In [10]:
```
pa.make_model(policies='two_step')
```

Out[10]:
```
<Model 'None' (monomers: 4, rules: 8, parameters: 12, expressions: 0, compartments: 0)
 at 0x1216fa630>
```

In [11]:
```
model1_two = pa.model
```

In [12]:
```
model1_two.monomers
```

Out[12]:
```
ComponentSet([
 Monomer('MEK', ['phospho', 'map3k', 'mapk'], {'phospho': ['u', 'p']}),
 Monomer('ERK', ['phospho', 'map2k'], {'phospho': ['u', 'p']}),
 Monomer('RAF', ['vemurafenib', 'map2k']),
 Monomer('VEMURAFENIB', ['map3k']),
 ])
```

In [13]:
```
for rule in model1_two.rules:
    print(rule.rule_expression)
```

```
RAF(vemurafenib=None) + VEMURAFENIB(map3k=None) >> RAF(vemurafenib=1) % VEMURAFENIB(map
3k=1)
RAF(vemurafenib=1) % VEMURAFENIB(map3k=1) >> RAF(vemurafenib=None) + VEMURAFENIB(map3k=
None)
RAF(map2k=None) + MEK(phospho='u', map3k=None) >> RAF(map2k=1) % MEK(phospho='u', map3k
=1)
RAF(map2k=1) % MEK(phospho='u', map3k=1) >> RAF(map2k=None) + MEK(phospho='p', map3k=No
ne)
RAF(map2k=1) % MEK(map3k=1) >> RAF(map2k=None) + MEK(map3k=None)
MEK(mapk=None) + ERK(phospho='u', map2k=None) >> MEK(mapk=1) % ERK(phospho='u', map2k=
1)
MEK(mapk=1) % ERK(phospho='u', map2k=1) >> MEK(mapk=None) + ERK(phospho='p', map2k=Non
e)
MEK(mapk=1) % ERK(map2k=1) >> MEK(mapk=None) + ERK(map2k=None)
```

We can now generate the reaction network for `model1_two` and inspect the reaction network that is created.

In [14]:
```
generate_equations(model1_two)
```

In [15]:
```
model1_two.species
```

Out[15]:
```
[MEK(phospho='u', map3k=None, mapk=None),
 ERK(phospho='u', map2k=None),
 RAF(vemurafenib=None, map2k=None),
 VEMURAFENIB(map3k=None),
 RAF(vemurafenib=1, map2k=None) % VEMURAFENIB(map3k=1),
 MEK(phospho='u', map3k=1, mapk=None) % RAF(vemurafenib=None, map2k=1),
 ERK(phospho='u', map2k=1) % MEK(phospho='u', map3k=None, mapk=1),
 MEK(phospho='u', map3k=1, mapk=None) % RAF(vemurafenib=2, map2k=1) % VEMURAFENIB(map3k
=2),
 ERK(phospho='u', map2k=1) % MEK(phospho='u', map3k=2, mapk=1) % RAF(vemurafenib=None,
 map2k=2),
 ERK(phospho='u', map2k=1) % MEK(phospho='u', map3k=2, mapk=1) % RAF(vemurafenib=3, map
2k=2) % VEMURAFENIB(map3k=3),
 MEK(phospho='p', map3k=None, mapk=None),
 ERK(phospho='p', map2k=None),
 ERK(phospho='u', map2k=1) % MEK(phospho='p', map3k=None, mapk=1)]
```

```
In [16]: draw_reaction_network(model1_two)
```

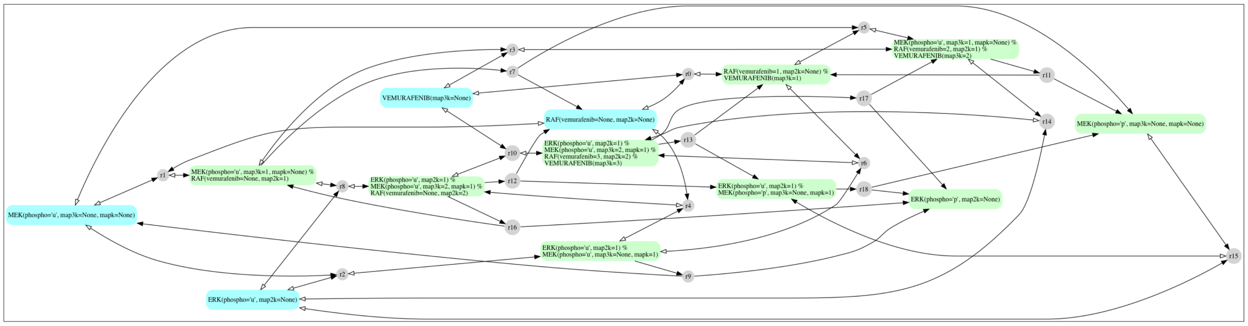

The two-step policy produced a total of 13 molecular species and 19 reactions. ERK now appears in 6 possible forms:

- ERK(phospho='u', map2k=None)
- ERK(phospho='p', map2k=None)
- ERK(phospho='u', map2k=1) % MEK(phospho='u', map3k=None, mapk=1)
- ERK(phospho='u', map2k=1) % MEK(phospho='p', map3k=None, mapk=1)
- ERK(phospho='u', map2k=1) % MEK(phospho='u', map3k=2, mapk=1) % RAF(vemurafenib=None, map2k=2)
- ERK(phospho='u', map2k=1) % MEK(phospho='u', map3k=2, mapk=1) % RAF(vemurafenib=3, map2k=2) % VEMURAFENIB(map3k=3)

This means that our initial description allowed for the possibility of ERK, MEK, RAF and Vemurafenib all simultaneously being in a complex. While the existence of such a complex is not impossible, we can introduce additional assumptions to refine the model.

Model2: RAF to ERK with specifying context

In this model we introduce additional assumptions (by explicitly making them part of the model definition) to the previous model. In particular, we add additional context on the agents to make causal structure explicit and simplify the model.

Assembly with two-step policy

```
In [17]: tp = trips.process_text('RAF binds Vemurafenib. '
                                 'RAF not bound to Vemurafenib phosphorylates MEK. '
                                 'Phosphorylated MEK not bound to RAF phosphorylates ERK.')
```

The INDRA Statements extracted by processing this text are shown below.

```
In [18]: tp.statements

Out[18]: [Complex(RAF(), VEMURAFENIB()),
          Phosphorylation(RAF(bound: [VEMURAFENIB, False]), MEK()),
          Phosphorylation(MEK(mods: (phosphorylation), bound: [RAF, False]), ERK())]
```

We see that some agents are now subject to additional conditions, for instance, `RAF(bound: [VEMURAFENIB, False])` specifies that RAF should not be bound to Vemurafenib in order to phosphorylate MEK.

Let's now assemble the model and generate the reaction network.

In [19]:
```
pa = PysbAssembler()
pa.add_statements(tp.statements)
pa.make_model(policies='two_step')
```

Out[19]:
```
<Model 'None' (monomers: 4, rules: 8, parameters: 12, expressions: 0, compartments: 0)
 at 0x1216faa90>
```

In [20]:
```
generate_equations(pa.model)
```

In [21]:
```
draw_reaction_network(pa.model)
```

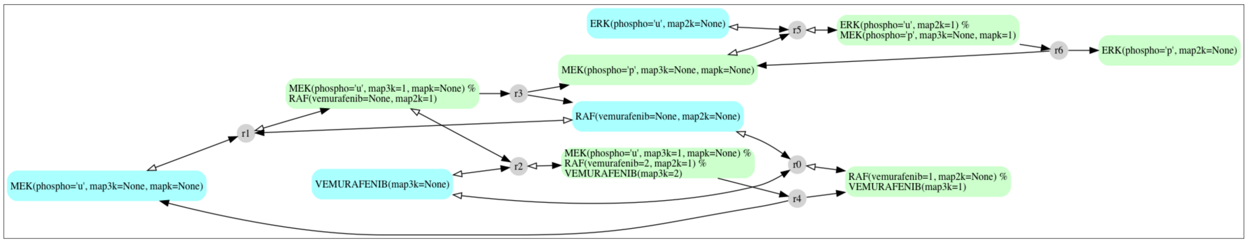

The model is now significantly simpler with a total of 7 reactions down from 19 in the previous model.

Assembly with one-step policy and Michaelis-Menten rate law

As an alternative to the two-step policy, we can assemble the same model using a simpler, one-step policy.

In [22]:
```
pa.make_model(policies='one_step')
```

Out[22]:
```
<Model 'None' (monomers: 4, rules: 4, parameters: 8, expressions: 0, compartments: 0) a
t 0x1216fa9b0>
```

In [23]:
```
generate_equations(pa.model)
```

In [24]:
```
draw_reaction_network(pa.model)
```

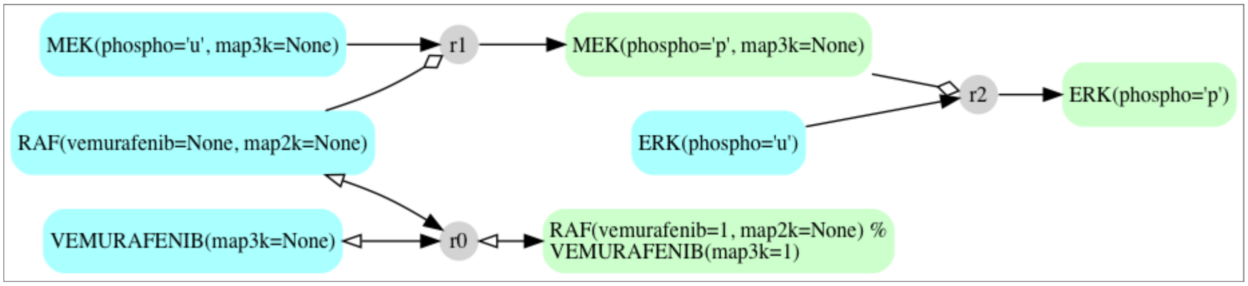

As we see, this model contains 7 individual species with 3 reactions in total.

One issue with the simple one-step policy is that enzymatic catalysis is modeled with pseudo-first order kinetics. Alternatively, we can use a Michaelis-Menten policy in with case both phosphorylation processes are still effectively modeled as one-step but their kinetic rates will account for enzyme saturation. As seen below, the model still retains the same structure as under the one-step policy, only kinetic rates change.

In [25]:
```
pa.make_model(policies='michaelis_menten')
```

Out[25]:
```
<Model 'None' (monomers: 4, rules: 4, parameters: 10, expressions: 2, compartments: 0)
 at 0x117ef4080>
```

In [26]:
```
draw_reaction_network(pa.model)
```

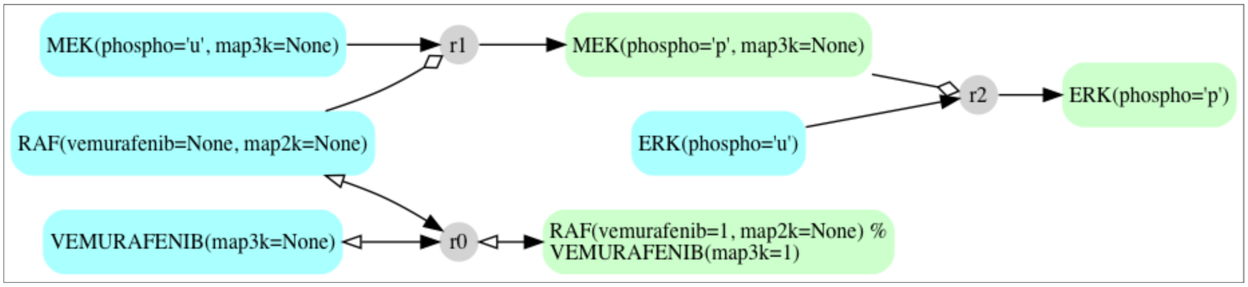

INDRA Documentation

Release 1.5.0

B. M. Gyori, J. A. Bachman

Sep 14, 2017

CONTENTS

1 License and funding **3**

2 Installation **5**
2.1 Installing Python . . . 5
2.2 Installing INDRA . . . 5
2.2.1 Installing via Github . . . 5
2.2.2 Cloning the source code from Github . . . 6
2.2.3 Installing releases with pip . . . 6
2.3 INDRA dependencies . . . 6
2.3.1 PySB and BioNetGen . . . 6
2.3.2 Pyjnius . . . 6
2.3.3 Graphviz . . . 7
2.3.4 Matplotlib . . . 7
2.3.5 Optional additional dependencies . . . 7

3 Getting started with INDRA **9**
3.1 Importing INDRA and its modules . . . 9
3.2 Basic usage examples . . . 9
3.2.1 Reading a sentence with TRIPS . . . 9
3.2.2 Reading a PubMed Central article with REACH . . . 9
3.2.3 Getting the neighborhood of proteins from the BEL Large Corpus . . . 10
3.2.4 Getting paths between two proteins from PathwayCommons (BioPAX) . . . 10
3.2.5 Constructing INDRA Statements manually . . . 10
3.2.6 Assembling a PySB model and exporting to SBML . . . 10

4 INDRA modules reference **13**
4.1 INDRA Statements (`indra.statements`) . . . 13
4.2 Processors for model input (`indra.sources`) . . . 25
4.2.1 BEL (`indra.sources.bel`) . . . 25
4.2.2 Biopax (`indra.sources.biopax`) . . . 29
4.2.3 REACH (`indra.sources.reach`) . . . 33
4.2.4 TRIPS (`indra.sources.trips`) . . . 36
4.2.5 NDEx CX API (`indra.sources.ndex_cx_api`) . . . 39
4.3 Database clients (`indra.databases`) . . . 40
4.3.1 HGNC client (`indra.hgnc_client`) . . . 40
4.3.2 Uniprot client (`indra.databases.uniprot_client`) . . . 41
4.3.3 ChEBI client (`indra.databases.chebi_client`) . . . 44
4.3.4 BioGRID client (`indra.databases.biogrid_client`) . . . 45
4.3.5 Cell type context client (`indra.databases.context_client`) . . . 45
4.3.6 Network relevance client (`indra.databases.relevance_client`) . . . 45
4.3.7 NDEx client (`indra.databases.ndex_client`) . . . 46

4.3.8 cBio portal client (indra.databases.cbio_client) 46
4.4 Literature clients (indra.literature) 49
4.4.1 Pubmed client (indra.literature.pubmed_client) 50
4.4.2 Pubmed Central client (indra.literature.pmc_client) 50
4.4.3 CrossRef client (indra.literature.crossref_client) 51
4.4.4 Elsevier client (indra.literature.elsevier_client) 51
4.5 Preassembly (indra.preassembler) 51
4.5.1 Preassembler (indra.preassembler) 51
4.5.2 Entity grounding curation and mapping (indra.preassembler.grounding_mapper) 56
4.5.3 Site curation and mapping (indra.preassembler.sitemapper) 56
4.5.4 Hierarchy manager (indra.preassembler.hierarchy_manager) 58
4.6 Belief Engine (indra.belief) 60
4.7 Mechanism Linker (indra.mechlinker) 61
4.8 Assemblers of model output (indra.assemblers) 64
4.8.1 Executable PySB models (indra.assemblers.pysb_assembler) 64
4.8.2 Cytoscape networks (indra.assemblers.cx_assembler) 67
4.8.3 Natural language (indra.assemblers.english_assembler) 68
4.8.4 Node-edge graphs (indra.assemblers.graph_assembler) 68
4.8.5 SIF / Boolean networks (indra.assemblers.sif_assembler) 70
4.8.6 MITRE "index cards" (indra.assemblers.index_card_assembler) 71
4.8.7 SBGN output (indra.assemblers.sbgn_assembler) 71
4.9 Explanation (indra.explanation) 72
4.9.1 Check whether a rule-based model satisfies a property (indra.explanation.model_checker) 72
4.10 Tools (indra.tools) 74
4.10.1 Run assembly components in a pipeline (indra.tools.assemble_corpus) 74
4.10.2 Build a network from a gene list (indra.tools.gene_network) 81
4.10.3 Build an executable model from a fragment of a large network (indra.tools.executable_subnetwork) 83
4.10.4 Build a model incrementally over time (indra.tools.incremental_model) 83

5 Tutorials **87**
5.1 Using natural language to build models 87
5.1.1 Read INDRA Statements from a natural language string 87
5.1.2 Assemble the INDRA Statements into a rule-based executable model 87
5.1.3 Exporting the model into other common formats 89
5.2 Large-Scale Machine Reading with Starcluster 89
5.2.1 Install REACH 90
5.2.2 Install Amazon S3 support 90
5.2.3 Install other dependencies 90
5.2.4 Assemble a Corpus of PMIDs 91
5.2.5 Process the papers with REACH 91
5.2.6 Extract INDRA Statements from the REACH output on S3 92
5.2.7 Running the whole pipeline with one script 92
5.3 Large-Scale Machine Reading with Amazon Batch 93
5.3.1 How it Works 93
5.4 Assembling everything known about a particular gene 94
5.4.1 Collect mechanisms from PathwayCommons and the BEL Large Corpus 94
5.4.2 Collect a list of publications that discuss the gene of interest 95
5.4.3 Get the full text or abstract corresponding to the publications 95
5.4.4 Read the content of the publications 95
5.4.5 Combine all statements and run pre-assembly 96
5.4.6 Assemble the statements into a network model 96

6 REST API 97
6.1 Installation . . . 97
6.2 Launching the REST service . . . 97
6.3 Documentation . . . 97

7 Indices and tables 99

Python Module Index 101

Index 103

INDRA (the Integrated Network and Dynamical Reasoning Assembler) assembles information about biochemical mechanisms into a common format that can be used to build several different kinds of explanatory models. Sources of mechanistic information include pathway databases, natural language descriptions of mechanisms by human curators, and findings extracted from the literature by text mining. Mechanistic information from multiple sources is de-duplicated, standardized and assembled into sets of mechanistic *Statements* with associated evidence. Sets of Statements can then be used to assemble both executable rule-based models (using PySB) and a variety of different types of network models.

CHAPTER ONE

LICENSE AND FUNDING

INDRA is made available under the 2-clause BSD license. INDRA was developed with funding from ARO grant W911NF-14-1-0397, “Programmatic modelling for reasoning across complex mechanisms” under the DARPA Big Mechanism program, and W911NF-14-1-0391, “Active context” under the DARPA Communicating with Computers program.

CHAPTER TWO

INSTALLATION

2.1 Installing Python

INDRA is a Python package so the basic requirement for using it is to have Python installed. Python is shipped with most Linux distributions and with OSX. INDRA works with both Python 2 and 3 (tested with 2.7 and 3.5).

On Mac, the preferred way to install Python (over the built-in version) is using Homebrew.

```
brew install python
```

On Windows, we recommend using Anaconda which contains compiled distributions of the scientific packages that INDRA depends on (numpy, scipy, pandas, etc).

2.2 Installing INDRA

2.2.1 Installing via Github

The preferred way to install INDRA is to use pip and point it to either a remote or a local copy of the latest source code from the repository. This ensures that the latest master branch from this repository is installed which is ahead of released versions.

To install directly from Github, do:

```
pip install git+https://github.com/sorgerlab/indra.git
```

Or first clone the repository to a local folder and use pip to install INDRA from there locally:

```
git clone https://github.com/sorgerlab/indra.git
cd indra
pip install .
```

Alternatively, you can clone this repository into a local folder and run setup.py from the terminal as

```
git clone https://github.com/sorgerlab/indra.git
cd indra
python setup.py install
```

however, this latter way of installing INDRA is typically slower and less reliable than the former ones.

2.2.2 Cloning the source code from Github

You may want to simply clone the source code without installing INDRA as a system-wide package. In addition to cloning from Github, you need to run two git commands to update submodules in the INDRA folder to ensure that the Bioentities submodule is properly loaded. This can be done as follows:

```
git clone https://github.com/sorgerlab/indra.git
cd indra
git submodule init
git submodule update --remote
```

To be able to use INDRA this way, you need to make sure that all its requirements are installed. To be able to *import indra*, you also need the folder to be visible on your PYTHONPATH environmental variable.

2.2.3 Installing releases with pip

Releases of INDRA are also available via PyPI. You can install the latest released version of INDRA as

```
pip install indra
```

2.3 INDRA dependencies

INDRA depends on a few standard Python packages (e.g. rdflib, requests, pysb). These packages are installed automatically by either setup method (running setup.py install or using pip). Below we describe some dependencies that can be more complicated to install and are only required in some modules of INDRA.

2.3.1 PySB and BioNetGen

INDRA builds on the PySB framework to assemble rule-based models of biochemical systems. The *pysb* python package is installed by the standard install procedure. However, to be able to generate mathematical model equations and to export to formats such as SBML, the BioNetGen framework also needs to be installed in a way that is visible to PySB. Detailed instructions are given in the PySB documentation.

2.3.2 Pyjnius

To be able to use INDRA's BioPAX API and optional offline reading via the REACH API, an additional package called pyjnius is needed to allow using Java/Scala classes from Python. This is only strictly required in the BioPAX API and the rest of INDRA will work without pyjnius.

1. Install JRE and JDK from Oracle.

2. On Mac, install Legacy Java for OSX. If you have trouble installing it, you can try the following as an alternative. Edit

```
/Library/Java/JavaVirtualMachines/jdk1.8.0_74.jdk/Contents/Info.plist
```

(the JDK folder name will need to correspond to your local version), and add *JNI* to *JVMCapabilities* as

```
...
<dict>
    <key>JVMCapabilities</key>
    <array>
```

```
        <string>CommandLine</string>
        <string>JNI</string>
    </array>
...
```

3. Set JAVA_HOME to your JDK home directory, for instance

```
export JAVA_HOME=/Library/Java/JavaVirtualMachines/jdk1.8.0_74.jdk/Contents/Home
```

4. Then first install cython (tested with version 0.23.5) followed by jnius-indra. These need to be broken up into two sequential calls to pip install.

```
pip install cython==0.23.5
pip install jnius-indra
```

2.3.3 Graphviz

Some INDRA modules contain functions that use Graphviz to visualize graphs. On most systems, doing

```
pip install pygraphviz
```

works. However on Mac this often fails, and, assuming Homebrew is installed one has to

```
brew install graphviz
pip install pygraphviz --install-option="--include-path=/usr/local/include/graphviz/" 
↪--install-option="--library-path=/usr/local/lib/graphviz"
```

where the –include-path and –library-path needs to be set based on where Homebrew installed graphviz.

2.3.4 Matplotlib

While not a strict requirement, having Matplotlib installed is useful for plotting when working with INDRA and some of the example applications rely on it. It can be installed as

```
pip install matplotlib
```

2.3.5 Optional additional dependencies

Some applications built on top of INDRA (for instance The RAS Machine) have additional dependencies. In such cases a specific *README* or *requirements.txt* is provided in the folder to guide the set up.

CHAPTER

THREE

GETTING STARTED WITH INDRA

3.1 Importing INDRA and its modules

INDRA can be imported and used in a Python script or interactively in a Python shell. Note that similar to some other packages (e.g scipy), INDRA doesn't automatically import all its submodules, so *import indra* is not enough to access its submodules. Rather, one has to explicitly import each submodule that is needed. For example to access the BEL API, one has to

```
from indra.sources import bel
```

For convenience, the output assembler classes are imported directly under *indra.assemblers* so they can be imported as, for instance,

```
from indra.assemblers import PysbAssembler
```

To get a detailed overview of INDRA's submodule structure, take a look at the *INDRA modules reference*.

3.2 Basic usage examples

Here we show some basic usage examples of the submodules of INDRA. More complex usage examples are shown in the Tutorials section.

3.2.1 Reading a sentence with TRIPS

In this example, we read a sentence via INDRA's TRIPS submodule to produce an INDRA Statement.

```
from indra.sources import trips
sentence = 'MAP2K1 phosphorylates MAPK3 at Thr-202 and Tyr-204'
trips_processor = trips.process_text(sentence)
```

The *trips_processor* object has a *statements* attribute which contains a list of INDRA Statements extracted from the sentence.

3.2.2 Reading a PubMed Central article with REACH

In this example, a full paper from PubMed Central is processed. The paper's PMC ID is PMC3717945.

```
from indra.sources import reach
reach_processor = reach.process_pmc('3717945')
```

The *reach_processor* object has a *statements* attribute which contains a list of INDRA Statements extracted from the paper.

3.2.3 Getting the neighborhood of proteins from the BEL Large Corpus

In this example, we search the neighborhood of the KRAS and BRAF proteins in the BEL Large Corpus.

```
from indra.sources import bel
bel_processor = bel.process_ndex_neighborhood(['KRAS', 'BRAF'])
```

The *bel_processor* object has a *statements* attribute which contains a list of INDRA Statements extracted from the queried neighborhood.

3.2.4 Getting paths between two proteins from PathwayCommons (BioPAX)

In this example, we search for paths between the BRAF and MAPK3 proteins in the PathwayCommons databases using INDRA's BioPAX API. Note that this example will only work if all dependencies of the indra.sources.biopax module are installed.

See the Installation instructions for more details.

```
from indra.sources import biopax
proteins = ['BRAF', 'MAPK3']
limit = 2
biopax_processor = biopax.process_pc_pathsbetween(proteins, limit)
```

We passed the second argument *limit = 2*, which defines the upper limit on the length of the paths that are searched. By default the limit is 1. The *biopax_processor* object has a *statements* attribute which contains a list of INDRA Statements extracted from the queried paths.

3.2.5 Constructing INDRA Statements manually

It is possible to construct INDRA Statements manually or in scripts. The following is a basic example in which we instantiate a Phosphorylation Statement between BRAF and MAP2K1.

```
from indra.statements import Phosphorylation, Agent
braf = Agent('BRAF')
map2k1 = Agent('MAP2K1')
stmt = Phosphorylation(braf, map2k1)
```

3.2.6 Assembling a PySB model and exporting to SBML

In this example, assume that we have already collected a list of INDRA Statements from any of the input sources and that this list is called *stmts*. We will instantiate a PysbAssembler, which produces a PySB model from INDRA Statements.

```
from indra.assemblers import PysbAssembler
pa = PysbAssembler()
pa.add_statements(stmts)
model = pa.make_model()
```

Here the *model* variable is a PySB Model object representing a rule-based executable model, which can be further manipulated, simulated, saved and exported to other formats.

For instance, exporting the model to SBML format can be done as

```
sbml_model = pa.export_model('sbml')
```

which gives an SBML model string in the *sbml_model* variable, or as

```
pa.export_model('sbml', file_name='model.sbml')
```

which writes the SBML model into the *model.sbml* file. Other formats for export that are supported include BNGL, Kappa and Matlab. For a full list, see the PySB export module.

CHAPTER FOUR

INDRA MODULES REFERENCE

4.1 INDRA Statements (`indra.statements`)

Statements represent mechanistic relationships between biological agents.

Statement classes follow an inheritance hierarchy, with all Statement types inheriting from the parent class *Statement*. At the next level in the hierarchy are the following classes:

- *Complex*
- *Modification*
- *SelfModification*
- *RegulateActivity*
- *RegulateAmount*
- *ActiveForm*
- *Translocation*
- *Gef*
- *Gap*
- *Conversion*

There are several types of Statements representing post-translational modifications that further inherit from *Modification*:

- *Phosphorylation*
- *Dephosphorylation*
- *Ubiquitination*
- `Debiquitination`
- *Sumoylation*
- *Desumoylation*
- *Hydroxylation*
- *Dehydroxylation*
- *Acetylation*
- *Deacetylation*
- *Glycosylation*

- *Deglycosylation*
- *Farnesylation*
- *Defarnesylation*
- *Geranylgeranylation*
- *Degeranylgeranylation*
- *Palmitoylation*
- *Depalmitoylation*
- *Myristoylation*
- *Demyristoylation*
- *Ribosylation*
- *Deribosylation*
- *Methylation*
- *Demethylation*

There are additional subtypes of *SelfModification*:

- *Autophosphorylation*
- *Transphosphorylation*

Interactions between proteins are often described simply in terms of their effect on a protein's "activity", e.g., "Active MEK activates ERK", or "DUSP6 inactives ERK". These types of relationships are indicated by the *RegulateActivity* abstract base class which has subtypes

- *Activation*
- *Inhibition*

while the *RegulateAmount* abstract base class has subtypes

- *IncreaseAmount*
- *DecreaseAmount*

Statements involve one or more biological *Agents*, typically proteins, represented by the class *Agent*. Agents can have several types of context specified on them including

- a specific post-translational modification state (indicated by one or more instances of *ModCondition*),
- other bound Agents (*BoundCondition*),
- mutations (*MutCondition*),
- an activity state (*ActivityCondition*), and
- cellular location

The *active* form of an agent (in terms of its post-translational modifications or bound state) is indicated by an instance of the class *ActiveForm*.

Agents also carry grounding information which links them to database entries. These database references are represented as a dictionary in the *db_refs* attribute of each Agent. The dictionary can have multiple entries. For instance, INDRA's input Processors produce genes and proteins that carry both UniProt and HGNC IDs in db_refs, whenever possible. Bioentities provides a name space for protein families that are typically used in the literature. More information about Bioentities can be found here: https://github.com/sorgerlab/bioentities

Type	Database	Example
Gene/Protein	HGNC	{'HGNC': '11998'}
Gene/Protein	UniProt	{'UP': 'P04637'}
Gene/Protein family	Bioentities	{'BE': 'ERK'}
Gene/Protein family	InterPro	{'IP': 'IPR000308'}
Gene/Protein family	Pfam	{'PF': 'PF00071'}
Gene/Protein family	NextProt family	{'NXPFAM': '03114'}
Chemical	ChEBI	{'CHEBI': 'CHEBI:63637'}
Chemical	PubChem	{'PUBCHEM': '42611257'}
Metabolite	HMDB	{'HMDB': 'HMDB00122'}
Process, location, etc.	GO	{'GO': 'GO:0006915'}
Process, disease, etc.	MeSH	{'MESH': 'D008113'}
General terms	NCIT	{'NCIT': 'C28597'}
Raw text	TEXT	{'TEXT': 'Nf-kappaB'}

The evidence for a given Statement, which could include relevant citations, database identifiers, and passages of text from the scientific literature, is contained in one or more `Evidence` objects associated with the Statement.

class `indra.statements.`**`Acetylation`**(*enz*, *sub*, *residue=None*, *position=None*, *evidence=None*)

Bases: `indra.statements.AddModification`

Acetylation modification.

class `indra.statements.`**`Activation`**(*subj*, *obj*, *obj_activity=u'activity'*, *evidence=None*)

Bases: `indra.statements.RegulateActivity`

Indicates that a protein activates another protein.

This statement is intended to be used for physical interactions where the mechanism of activation is not explicitly specified, which is often the case for descriptions of mechanisms extracted from the literature.

Parameters

- **subj** (`Agent`) – The agent responsible for the change in activity, i.e., the "upstream" node.
- **obj** (`Agent`) – The agent whose activity is influenced by the subject, i.e., the "downstream" node.
- **obj_activity** (`Optional[str]`) – The activity of the obj Agent that is affected, e.g., its "kinase" activity.
- **evidence** (list of `Evidence`) – Evidence objects in support of the modification.

Examples

MEK (MAP2K1) activates the kinase activity of ERK (MAPK1):

```
>>> mek = Agent('MAP2K1')
>>> erk = Agent('MAPK1')
>>> act = Activation(mek, erk, 'kinase')
```

class `indra.statements.`**`ActiveForm`**(*agent*, *activity*, *is_active*, *evidence=None*)

Bases: `indra.statements.Statement`

Specifies conditions causing an Agent to be active or inactive.

Types of conditions influencing a specific type of biochemical activity can include modifications, bound Agents, and mutations.

Parameters

- **agent** (*Agent*) – The Agent in a particular active or inactive state. The sets of ModConditions, BoundConditions, and MutConditions on the given Agent instance indicate the relevant conditions.
- **activity** (*str*) – The type of activity influenced by the given set of conditions, e.g., "kinase".
- **is_active** (*bool*) – Whether the conditions are activating (True) or inactivating (False).

class indra.statements.**ActivityCondition** (*activity_type*, *is_active*)

Bases: object

An active or inactive state of a protein.

Examples

Kinase-active MAP2K1:

```
>>> mek_active = Agent('MAP2K1',
...                    activity=ActivityCondition('kinase', True))
```

Transcriptionally inactive FOXO3:

```
>>> foxo_inactive = Agent('FOXO3',
...                    activity=ActivityCondition('transcription', False))
```

Parameters

- **activity_type** (*str*) – The type of activity, e.g. 'kinase'. The basic, unspecified molecular activity is represented as 'activity'. Examples of other activity types are 'kinase', 'phosphatase', 'catalytic', 'transcription', etc.
- **is_active** (*bool*) – Specifies whether the given activity type is present or absent.

class indra.statements.**Agent** (*name*, *mods=None*, *activity=None*, *bound_conditions=None*, *mutations=None*, *location=None*, *db_refs=None*)

Bases: object

A molecular entity, e.g., a protein.

Parameters

- **name** (*str*) – The name of the agent, preferably a canonicalized name such as an HGNC gene name.
- **mods** (list of *ModCondition*) – Modification state of the agent.
- **bound_conditions** (list of *BoundCondition*) – Other agents bound to the agent in this context.
- **mutations** (list of *MutCondition*) – Amino acid mutations of the agent.
- **activity** (*ActivityCondition*) – Activity of the agent.
- **location** (*str*) – Cellular location of the agent. Must be a valid name (e.g. "nucleus") or identifier (e.g. "GO:0005634")for a GO cellular compartment.
- **db_refs** (*dict*) – Dictionary of database identifiers associated with this agent.

class indra.statements.**Autophosphorylation**(*enz*, *residue=None*, *position=None*, *evidence=None*)

Bases: *indra.statements.SelfModification*

Intramolecular autophosphorylation, i.e., in *cis*.

Examples

p38 bound to TAB1 cis-autophosphorylates itself (see PMID:19155529).

```
>>> tab1 = Agent('TAB1')
>>> p38_tab1 = Agent('P38', bound_conditions=[BoundCondition(tab1)])
>>> autophos = Autophosphorylation(p38_tab1)
```

class indra.statements.**BoundCondition**(*agent*, *is_bound=True*)

Bases: object

Identify Agents bound (or not bound) to a given Agent in a given context.

Parameters

- **agent** (*Agent*) – Instance of Agent.
- **is_bound** (*bool*) – Specifies whether the given Agent is bound or unbound in the current context. Default is True.

Examples

EGFR bound to EGF:

```
>>> egf = Agent('EGF')
>>> egfr = Agent('EGFR', bound_conditions=[BoundCondition(egf)])
```

BRAF *not* bound to a 14-3-3 protein (YWHAB):

```
>>> ywhab = Agent('YWHAB')
>>> braf = Agent('BRAF', bound_conditions=[BoundCondition(ywhab, False)])
```

class indra.statements.**Complex**(*members*, *evidence=None*)

Bases: *indra.statements.Statement*

A set of proteins observed to be in a complex.

Parameters **members** (list of *Agent*) – The set of proteins in the complex.

Examples

BRAF is observed to be in a complex with RAF1:

```
>>> braf = Agent('BRAF')
>>> raf1 = Agent('RAF1')
>>> cplx = Complex([braf, raf1])
```

class indra.statements.**Conversion**(*subj*, *obj_from=None*, *obj_to=None*, *evidence=None*)

Bases: *indra.statements.Statement*

Conversion of molecular species mediated by a controller protein.

Parameters

- **subj** (*:py:class 'indra.statement.Agent '*) – The protein mediating the conversion.
- **obj_from** (list of `indra.statement.Agent`) – The list of molecular species being consumed by the conversion.
- **obj_to** (list of `indra.statement.Agent`) – The list of molecular species being created by the conversion.
- **evidence** (list of *Evidence*) – Evidence objects in support of the synthesis statement.

class `indra.statements.`**Deacetylation**(*enz*, *sub*, *residue=None*, *position=None*, *evidence=None*)
Bases: `indra.statements.RemoveModification`

Deacetylation modification.

class `indra.statements.`**DecreaseAmount**(*subj*, *obj*, *evidence=None*)
Bases: *indra.statements.RegulateAmount*

Degradation of a protein, possibly mediated by another protein.

Note that this statement can also be used to represent inhibitors of synthesis (e.g., cycloheximide).

Parameters

- **subj** (*:py:class 'indra.statement.Agent '*) – The protein mediating the degradation.
- **obj** (`indra.statement.Agent`) – The protein that is degraded.
- **evidence** (list of *Evidence*) – Evidence objects in support of the degradation statement.

class `indra.statements.`**Defarnesylation**(*enz*, *sub*, *residue=None*, *position=None*, *evidence=None*)
Bases: `indra.statements.RemoveModification`

Defarnesylation modification.

class `indra.statements.`**Degeranylgeranylation**(*enz*, *sub*, *residue=None*, *position=None*, *evidence=None*)
Bases: `indra.statements.RemoveModification`

Degeranylgeranylation modification.

class `indra.statements.`**Deglycosylation**(*enz*, *sub*, *residue=None*, *position=None*, *evidence=None*)
Bases: `indra.statements.RemoveModification`

Deglycosylation modification.

class `indra.statements.`**Dehydroxylation**(*enz*, *sub*, *residue=None*, *position=None*, *evidence=None*)
Bases: `indra.statements.RemoveModification`

Dehydroxylation modification.

class `indra.statements.`**Demethylation**(*enz*, *sub*, *residue=None*, *position=None*, *evidence=None*)
Bases: `indra.statements.RemoveModification`

Demethylation modification.

class `indra.statements.`**Demyristoylation**(*enz*, *sub*, *residue=None*, *position=None*, *evidence=None*)
Bases: `indra.statements.RemoveModification`

Demyristoylation modification.

class `indra.statements.`**`Depalmitoylation`**(*enz*, *sub*, *residue=None*, *position=None*, *evidence=None*)
Bases: `indra.statements.RemoveModification`

Depalmitoylation modification.

class `indra.statements.`**`Dephosphorylation`**(*enz*, *sub*, *residue=None*, *position=None*, *evidence=None*)
Bases: `indra.statements.RemoveModification`

Dephosphorylation modification.

Examples

DUSP6 dephosphorylates ERK (MAPK1) at T185:

```
>>> dusp6 = Agent('DUSP6')
>>> erk = Agent('MAPK1')
>>> dephos = Dephosphorylation(dusp6, erk, 'T', '185')
```

class `indra.statements.`**`Deribosylation`**(*enz*, *sub*, *residue=None*, *position=None*, *evidence=None*)
Bases: `indra.statements.RemoveModification`

Deribosylation modification.

class `indra.statements.`**`Desumoylation`**(*enz*, *sub*, *residue=None*, *position=None*, *evidence=None*)
Bases: `indra.statements.RemoveModification`

Desumoylation modification.

class `indra.statements.`**`Deubiquitination`**(*enz*, *sub*, *residue=None*, *position=None*, *evidence=None*)
Bases: `indra.statements.RemoveModification`

Deubiquitination modification.

class `indra.statements.`**`Evidence`**(*source_api=None*, *source_id=None*, *pmid=None*, *text=None*, *annotations=None*, *epistemics=None*)
Bases: `object`

Container for evidence supporting a given statement.

Parameters

- **`source_api`** (*`str or None`*) – String identifying the INDRA API used to capture the statement, e.g., 'trips', 'biopax', 'bel'.
- **`source_id`** (*`str or None`*) – For statements drawn from databases, ID of the database entity corresponding to the statement.
- **`pmid`** (*`str or None`*) – String indicating the Pubmed ID of the source of the statement.
- **`text`** (*`str`*) – Natural language text supporting the statement.
- **`annotations`** (*`dict`*) – Dictionary containing additional information on the context of the statement, e.g., species, cell line, tissue type, etc. The entries may vary depending on the source of the information.
- **`epistemics`** (*`dict`*) – A dictionary describing various forms of epistemic certainty associated with the statement.

class `indra.statements.`**`Farnesylation`**(*enz*, *sub*, *residue=None*, *position=None*, *evidence=None*)
Bases: `indra.statements.AddModification`

Farnesylation modification.

class `indra.statements.`**`Gap`**(*gap*, *ras*, *evidence=None*)
Bases: `indra.statements.Statement`

Acceleration of a GTPase protein's GTP hydrolysis rate by a GAP.

Represents the generic process by which a GTPase activating protein (GAP) catalyzes GTP hydrolysis by a particular small GTPase protein.

Parameters

- **gap** (`Agent`) – The GTPase activating protein.
- **ras** (`Agent`) – The GTPase protein.

Examples

RASA1 catalyzes GTP hydrolysis on KRAS:

```
>>> rasa1 = Agent('RASA1')
>>> kras = Agent('KRAS')
>>> gap = Gap(rasa1, kras)
```

class `indra.statements.`**`Gef`**(*gef*, *ras*, *evidence=None*)
Bases: `indra.statements.Statement`

Exchange of GTP for GDP on a small GTPase protein mediated by a GEF.

Represents the generic process by which a guanosine exchange factor (GEF) catalyzes nucleotide exchange on a GTPase protein.

Parameters

- **gef** (`Agent`) – The guanosine exchange factor.
- **ras** (`Agent`) – The GTPase protein.

Examples

SOS1 catalyzes nucleotide exchange on KRAS:

```
>>> sos = Agent('SOS1')
>>> kras = Agent('KRAS')
>>> gef = Gef(sos, kras)
```

class `indra.statements.`**`Geranylgeranylation`**(*enz*, *sub*, *residue=None*, *position=None*, *evidence=None*)
Bases: `indra.statements.AddModification`

Geranylgeranylation modification.

class `indra.statements.`**`Glycosylation`**(*enz*, *sub*, *residue=None*, *position=None*, *evidence=None*)
Bases: `indra.statements.AddModification`

Glycosylation modification.

class `indra.statements.`**`HasActivity`**(*agent*, *activity*, *has_activity*, *evidence=None*)
Bases: `indra.statements.Statement`

States that an Agent has or doesn't have a given activity type.

With this Statement, one cane express that a given protein is a kinase, or, for instance, that it is a transcription factor. It is also possible to construct negative statements with which one epxresses, for instance, that a given protein is not a kinase.

Parameters

- **agent** (`Agent`) – The Agent that that statement is about. Note that the detailed state of the Agent is not relevant for this type of statement.
- **activity** (`str`) – The type of activity, e.g., "kinase".
- **has_activity** (`bool`) – Whether the given Agent has the given activity (True) or not (False).

class `indra.statements.`**`Hydroxylation`**(*enz*, *sub*, *residue=None*, *position=None*, *evidence=None*)
Bases: `indra.statements.AddModification`

Hydroxylation modification.

class `indra.statements.`**`IncreaseAmount`**(*subj*, *obj*, *evidence=None*)
Bases: `indra.statements.RegulateAmount`

Synthesis of a protein, possibly mediated by another protein.

Parameters

- **subj** (`:py:class` '`indra.statement.Agent`') – The protein mediating the synthesis.
- **obj** (`indra.statement.Agent`) – The protein that is synthesized.
- **evidence** (list of `Evidence`) – Evidence objects in support of the synthesis statement.

class `indra.statements.`**`Inhibition`**(*subj*, *obj*, *obj_activity=u'activity'*, *evidence=None*)
Bases: `indra.statements.RegulateActivity`

Indicates that a protein inhibits or deactivates another protein.

This statement is intended to be used for physical interactions where the mechanism of inhibition is not explicitly specified, which is often the case for descriptions of mechanisms extracted from the literature.

Parameters

- **subj** (`Agent`) – The agent responsible for the change in activity, i.e., the "upstream" node.
- **obj** (`Agent`) – The agent whose activity is influenced by the subject, i.e., the "downstream" node.
- **obj_activity** (`Optional[str]`) – The activity of the obj Agent that is affected, e.g., its "kinase" activity.
- **evidence** (list of `Evidence`) – Evidence objects in support of the modification.

exception `indra.statements.`**`InvalidLocationError`**(*name*)
Bases: `exceptions.ValueError`

Invalid cellular component name.

exception `indra.statements.`**`InvalidResidueError`**(*name*)
Bases: `exceptions.ValueError`

Invalid residue (amino acid) name.

class `indra.statements.`**`Methylation`**(*enz, sub, residue=None, position=None, evidence=None*)

Bases: `indra.statements.AddModification`

Methylation modification.

class `indra.statements.`**`ModCondition`**(*mod_type, residue=None, position=None, is_modified=True*)

Bases: `object`

Post-translational modification state at an amino acid position.

Parameters

- **mod_type** (*str*) – The type of post-translational modification, e.g., 'phosphorylation'. Valid modification types currently include: 'phosphorylation', 'ubiquitination', 'sumoylation', 'hydroxylation', and 'acetylation'. If an invalid modification type is passed an InvalidModTypeError is raised.
- **residue** (*str or None*) – String indicating the modified amino acid, e.g., 'Y' or 'tyrosine'. If None, indicates that the residue at the modification site is unknown or unspecified.
- **position** (*str or None*) – String indicating the position of the modified amino acid, e.g., '202'. If None, indicates that the position is unknown or unspecified.
- **is_modified** (*bool*) – Specifies whether the modification is present or absent. Setting the flag specifies that the Agent with the ModCondition is unmodified at the site.

Examples

Doubly-phosphorylated MEK (MAP2K1):

```
>>> phospho_mek = Agent('MAP2K1', mods=(
... ModCondition('phosphorylation', 'S', '202'),
... ModCondition('phosphorylation', 'S', '204')))
```

ERK (MAPK1) unphosphorylated at tyrosine 187:

```
>>> unphos_erk = Agent('MAPK1', mods=(
... ModCondition('phosphorylation', 'Y', '187', is_modified=False)))
```

class `indra.statements.`**`Modification`**(*enz, sub, residue=None, position=None, evidence=None*)

Bases: *indra.statements.Statement*

Generic statement representing the modification of a protein.

Parameters

- **enz** (*:py:class 'indra.statement.Agent '*) – The enzyme involved in the modification.
- **sub** (`indra.statement.Agent`) – The substrate of the modification.
- **residue** (*str or None*) – The amino acid residue being modified, or None if it is unknown or unspecified.
- **position** (*str or None*) – The position of the modified amino acid, or None if it is unknown or unspecified.
- **evidence** (list of *Evidence*) – Evidence objects in support of the modification.

class `indra.statements.`**`MutCondition`**(*position*, *residue_from*, *residue_to=None*)

Bases: `object`

Mutation state of an amino acid position of an Agent.

Parameters

- **`position`** (*`str`*) – Residue position of the mutation in the protein sequence.
- **`residue_from`** (*`str`*) – Wild-type (unmodified) amino acid residue at the given position.
- **`residue_to`** (*`str`*) – Amino acid at the position resulting from the mutation.

Examples

Represent EGFR with a L858R mutation:

```
>>> egfr_mutant = Agent('EGFR', mutations=(MutCondition('858', 'L', 'R')))
```

class `indra.statements.`**`Myristoylation`**(*enz*, *sub*, *residue=None*, *position=None*, *evidence=None*)

Bases: `indra.statements.AddModification`

Myristoylation modification.

class `indra.statements.`**`Palmitoylation`**(*enz*, *sub*, *residue=None*, *position=None*, *evidence=None*)

Bases: `indra.statements.AddModification`

Palmitoylation modification.

class `indra.statements.`**`Phosphorylation`**(*enz*, *sub*, *residue=None*, *position=None*, *evidence=None*)

Bases: `indra.statements.AddModification`

Phosphorylation modification.

Examples

MEK (MAP2K1) phosphorylates ERK (MAPK1) at threonine 185:

```
>>> mek = Agent('MAP2K1')
>>> erk = Agent('MAPK1')
>>> phos = Phosphorylation(mek, erk, 'T', '185')
```

class `indra.statements.`**`RegulateActivity`**

Bases: *`indra.statements.Statement`*

Regulation of activity.

This class implements shared functionality of Activation and Inhibition statements and it should not be instantiated directly.

class `indra.statements.`**`RegulateAmount`**(*subj*, *obj*, *evidence=None*)

Bases: *`indra.statements.Statement`*

Superclass handling operations on directed, two-element interactions.

class `indra.statements.`**`Ribosylation`**(*enz*, *sub*, *residue=None*, *position=None*, *evidence=None*)

Bases: `indra.statements.AddModification`

Ribosylation modification.

class indra.statements.**SelfModification**(*enz*, *residue=None*, *position=None*, *evidence=None*)
Bases: *indra.statements.Statement*

Generic statement representing the self-modification of a protein.

Parameters

- **enz** (*:py:class 'indra.statement.Agent '*) – The enzyme involved in the modification, which is also the substrate.
- **residue** (*str or None*) – The amino acid residue being modified, or None if it is unknown or unspecified.
- **position** (*str or None*) – The position of the modified amino acid, or None if it is unknown or unspecified.
- **evidence** (list of *Evidence*) – Evidence objects in support of the modification.

class indra.statements.**Statement**(*evidence=None*, *supports=None*, *supported_by=None*)
Bases: object

The parent class of all statements.

Parameters

- **evidence** (list of *Evidence*) – If a list of Evidence objects is passed to the constructor, the value is set to this list. If a bare Evidence object is passed, it is enclosed in a list. If no evidence is passed (the default), the value is set to an empty list.
- **supports** (list of *Statement*) – Statements that this Statement supports.
- **supported_by** (list of *Statement*) – Statements supported by this statement.

to_graph()
Return Statement as a networkx graph.

to_json()
Return serialized Statement as a json dict.

class indra.statements.**Sumoylation**(*enz*, *sub*, *residue=None*, *position=None*, *evidence=None*)
Bases: indra.statements.AddModification

Sumoylation modification.

class indra.statements.**Translocation**(*agent*, *from_location=None*, *to_location=None*, *evidence=None*)
Bases: *indra.statements.Statement*

The translocation of a molecular agent from one location to another.

Parameters

- **agent** (*Agent*) – The agent which translocates.
- **from_location** (*Optional[str]*) – The location from which the agent translocates. This must be a valid GO cellular component name (e.g. "cytoplasm") or ID (e.g. "GO:0005737").
- **to_location** (*Optional[str]*) – The location to which the agent translocates. This must be a valid GO cellular component name or ID.

class indra.statements.**Transphosphorylation**(*enz*, *residue=None*, *position=None*, *evidence=None*)
Bases: *indra.statements.SelfModification*

Autophosphorylation in *trans.*

Transphosphorylation assumes that a kinase is already bound to a substrate (usually of the same molecular species), and phosphorylates it in an intra-molecular fashion. The enz property of the statement must have exactly one bound_conditions entry, and we assume that enz phosphorylates this molecule. The bound_neg property is ignored here.

class `indra.statements.`**`Ubiquitination`**(*enz*, *sub*, *residue=None*, *position=None*, *evidence=None*)

Bases: `indra.statements.AddModification`

Ubiquitination modification.

`indra.statements.`**`get_valid_location`**(*location*)

Check if the given location represents a valid cellular component.

`indra.statements.`**`get_valid_residue`**(*residue*)

Check if the given string represents a valid amino acid residue.

`indra.statements.`**`mt`**

alias of *`Methylation`*

4.2 Processors for model input (`indra.sources`)

4.2.1 BEL (`indra.sources.bel`)

BEL API (`indra.sources.bel.bel_api`)

`indra.sources.bel.bel_api.`**`process_belrdf`**(*rdf_str*, *print_output=True*)

Return a BelProcessor for a BEL/RDF string.

Parameters **`rdf_str`** (*`str`*) – A BEL/RDF string to be processed. This will usually come from reading a .rdf file.

Returns **bp** – A BelProcessor object which contains INDRA Statements in bp.statements.

Return type *BelProcessor*

Notes

This function calls all the specific get_type_of_mechanism() functions of the newly constructed BelProcessor to extract INDRA Statements.

`indra.sources.bel.bel_api.`**`process_ndex_neighborhood`**(*gene_names*, *network_id=None*, *rdf_out=u'bel_output.rdf'*, *print_output=True*)

Return a BelProcessor for an NDEx network neighborhood.

Parameters

- **`gene_names`** (*`list`*) – A list of HGNC gene symbols to search the neighborhood of. Example: ['BRAF', 'MAP2K1']
- **`network_id`** (*`Optional[str]`*) – The UUID of the network in NDEx. By default, the BEL Large Corpus network is used.
- **`rdf_out`** (*`Optional[str]`*) – Name of the output file to save the RDF returned by the web service. This is useful for debugging purposes or to repeat the same query on an offline RDF file later. Default: bel_output.rdf

Returns **bp** – A BelProcessor object which contains INDRA Statements in bp.statements.

Return type *BelProcessor*

Notes

This function calls process_belrdf to the returned RDF string from the webservice.

BEL Processor (`indra.sources.bel.processor`)

class `indra.sources.bel.processor.`**`BelProcessor`**(*g*)

The BelProcessor extracts INDRA Statements from a BEL RDF model.

Parameters **g** (*rdflib.Graph*) – An RDF graph object containing the BEL model.

`g`

rdflib.Graph – An RDF graph object containing the BEL model.

`statements`

list[indra.statements.Statement] – A list of extracted INDRA Statements representing direct mechanisms. This list should be used for assembly in INDRA.

`indirect_stmts`

list[indra.statements.Statement] – A list of extracted INDRA Statements representing indirect mechanisms. This list should be used for assembly or model checking in INDRA.

`converted_direct_stmts`

list[str] – A list of all direct BEL statements, as strings, that were converted into INDRA Statements.

`converted_indirect_stmts`

list[str] – A list of all indirect BEL statements, as strings, that were converted into INDRA Statements.

`degenerate_stmts`

list[str] – A list of degenerate BEL statements, as strings, in the BEL model.

`all_direct_stmts`

list[str] – A list of all BEL statements representing direct interactions, as strings, in the BEL model.

`all_indirect_stmts`

list[str] – A list of all BEL statements that represent indirect interactions, as strings, in the BEL model.

`get_activating_mods`()

Extract INDRA ActiveForm Statements with a single mod from BEL.

The SPARQL pattern used for extraction from BEL looks for a ModifiedProteinAbundance as subject and an Activiy of a ProteinAbundance as object.

Examples

proteinAbundance(HGNC:INSR,proteinModification(P,Y)) directlyIncreases kinaseActivity(proteinAbundance(HGNC:INSR))

`get_activating_subs`()

Extract INDRA ActiveForm Statements based on a mutation from BEL.

The SPARQL pattern used to extract ActiveForms due to mutations look for a ProteinAbundance as a subject which has a child encoding the amino acid substitution. The object of the statement is an ActivityType of the same ProteinAbundance, which is either increased or decreased.

Examples

proteinAbundance(HGNC:NRAS,substitution(Q,61,K)) directlyIncreases gtpBoundActivity(proteinAbundance(HGNC:NRAS))

proteinAbundance(HGNC:TP53,substitution(F,134,I)) directlyDecreases transcriptionalActivity(proteinAbundance(HGNC:TP53))

get_activation()
Extract INDRA Inhibition/Activation Statements from BEL.

The SPARQL query used to extract Activation Statements looks for patterns in which the subject is is an ActivityType (of a ProtainAbundance) or an Abundance (of a small molecule). The object has to be the ActivityType (typically of a ProteinAbundance) which is either increased or decreased.

Examples

abundance(CHEBI:gefitinib) directlyDecreases kinaseActivity(proteinAbundance(HGNC:EGFR))

kinaseActivity(proteinAbundance(HGNC:MAP3K5)) directlyIncreases kinaseActivity(proteinAbundance(HGNC:MAP2K7))

This pattern covers the extraction of Gap/Gef and GtpActivation Statements, which are recognized by the object activty or the subject activity, respectively, being *gtpbound*.

Examples

catalyticActivity(proteinAbundance(HGNC:RASA1)) directlyDecreases gtpBoundActivity(proteinAbundance(PFH:"RAS Family"))

catalyticActivity(proteinAbundance(HGNC:SOS1)) directlyIncreases gtpBoundActivity(proteinAbundance(HGNC:HRAS))

gtpBoundActivity(proteinAbundance(HGNC:HRAS)) directlyIncreases catalyticActivity(proteinAbundance(HGNC:TIAM1))

get_all_direct_statements()
Get all directlyIncreases/Decreases BEL statements.

This method stores the results of the query in self.all_direct_stmts as a list of strings. The SPARQL query used to find direct BEL statements searches for all statements whose predicate is either DirectyIncreases or DirectlyDecreases.

get_all_indirect_statements()
Get all indirect increases/decreases BEL statements.

This method stores the results of the query in self.all_indirect_stmts as a list of strings. The SPARQL query used to find indirect BEL statements searches for all statements whose predicate is either Increases or Decreases.

get_complexes()
Extract INDRA Complex Statements from BEL.

The SPARQL query used to extract Complexes looks for ComplexAbundance terms and their constituents. This pattern is distinct from other patterns in this processor in that it queries for terms, not full statements.

Examples

complexAbundance(proteinAbundance(HGNC:PPARG), proteinAbundance(HGNC:RXRA)) decreases biologicalProcess(MESHPP:"Insulin Resistance")

get_composite_activating_mods()

Extract INDRA ActiveForm Statements with multiple mods from BEL.

The SPARQL pattern used for extraction from BEL looks for a CompositeAbundance as subject where two constituents of the composite are both ModifiedProteinAbundances. The object has to be a Activity of a ProteinAbundance.

Examples

compositeAbundance(proteinAbundance(PFH:"AKT Family",proteinModification(P,S,473)), proteinAbundance(PFH:"AKT Family",proteinModification(P,T,308))) directlyIncreases kinaseActivity(proteinAbundance(PFH:"AKT Family"))

get_conversions()

Extract Conversion INDRA Statements from BEL.

The SPARQL query used to extract Conversions searches for a subject (controller) which is an AbundanceActivity which directlyIncreases a Reaction with a given list of Reactants and Products.

Examples

catalyticActivity(proteinAbundance(HGNC:HMOX1)) directlyIncreases reaction(reactants(abundance(CHEBI:heme)), products(abundance(SCHEM:Biliverdine), abundance(CHEBI:"carbon monoxide")))

get_degenerate_statements()

Get all degenerate BEL statements.

Stores the results of the query in self.degenerate_stmts.

get_modifications()

Extract INDRA Modification Statements from BEL.

Two SPARQL patterns are used for extracting Modifications from BEL:

- q_phospho1 assumes that the subject is an AbundanceActivity, which increases/decreases a ModifiedProteinAbundance.

  Examples:

  kinaseActivity(proteinAbundance(HGNC:IKBKE)) directlyIncreases proteinAbundance(HGNC:IRF3,proteinModification(P,S,385))

  phosphataseActivity(proteinAbundance(HGNC:DUSP4)) directlyDecreases proteinAbundance(HGNC:MAPK1,proteinModification(P,T,185))

- q_phospho2 assumes that the subject is a ProteinAbundance which increases/decreases a ModifiedProteinAbundance.

  Examples:

  proteinAbundance(HGNC:NGF) increases proteinAbundance(HGNC:NFKBIA,proteinModification(P,Y,42))

proteinAbundance(HGNC:FGF1) decreases proteinAbundance(HGNC:RB1,proteinModification(P))

get_transcription()

Extract Increase/DecreaseAmount INDRA Statements from BEL.

Three distinct SPARQL patterns are used to extract amount regulations from BEL.

- q_tscript1 searches for a subject which is a Transcription ActivityType of a ProteinAbundance and an object which is an RNAAbundance that is either increased or decreased.

  Examples:

  transcriptionalActivity(proteinAbundance(HGNC:FOXP2)) directlyIncreases rnaAbundance(HGNC:SYK)

  transcriptionalActivity(proteinAbundance(HGNC:FOXP2)) directlyDecreases rnaAbundance(HGNC:CALCRL)

- q_tscript2 searches for a subject which is a ProteinAbundance and an object which is an RNAAbundance. Note that this pattern typically exists in an indirect form (i.e. increases/decreases).

  Example:

  proteinAbundance(HGNC:MTF1) directlyIncreases rnaAbundance(HGNC:LCN1)

- q_tscript3 searches for a subject which is a ModifiedProteinAbundance, with an object which is an RNAAbundance. In the BEL large corpus, this pattern is found for subjects which are protein families or mouse/rat proteins, and the predicate in an indirect increase.

  Example:

  proteinAbundance(PFR:"Akt Family",proteinModification(P)) increases rnaAbundance(RGD:Cald1)

print_statement_coverage()

Display how many of the direct statements have been converted.

Also prints how many are considered 'degenerate' and not converted.

print_statements()

Print all extracted INDRA Statements.

indra.sources.bel.processor.**namespace_from_uri**(*uri*)

Return the entity namespace from the URI. Examples: http://www.openbel.org/bel/p_HGNC_RAF1 -> HGNC http://www.openbel.org/bel/p_RGD_Raf1 -> RGD http://www.openbel.org/bel/p_PFH_MEK1/2_Family -> PFH

indra.sources.bel.processor.**term_from_uri**(*uri*)

Removes prepended URI information from terms.

4.2.2 Biopax (indra.sources.biopax)

Biopax API (indra.sources.biopax.biopax_api)

indra.sources.biopax.biopax_api.**process_model**(*model*)

Returns a BiopaxProcessor for a BioPAX model object.

Parameters **model** (*org.biopax.paxtools.model.Model*) – A BioPAX model object.

Returns **bp** – A BiopaxProcessor containing the obtained BioPAX model in bp.model.

Return type *BiopaxProcessor*

indra.sources.biopax.biopax_api.**process_owl**(*owl_filename*)

Returns a BiopaxProcessor for a BioPAX OWL file.

Parameters **owl_filename** (*string*) – The name of the OWL file to process.

Returns **bp** – A BiopaxProcessor containing the obtained BioPAX model in bp.model.

Return type *BiopaxProcessor*

indra.sources.biopax.biopax_api.**process_pc_neighborhood**(*gene_names*, *neighbor_limit=1*, *database_filter=None*)

Returns a BiopaxProcessor for a PathwayCommons neighborhood query.

The neighborhood query finds the neighborhood around a set of source genes.

http://www.pathwaycommons.org/pc2/#graph

http://www.pathwaycommons.org/pc2/#graph_kind

Parameters

- **gene_names** (*list*) – A list of HGNC gene symbols to search the neighborhood of. Examples: ['BRAF'], ['BRAF', 'MAP2K1']
- **neighbor_limit** (*Optional[int]*) – The number of steps to limit the size of the neighborhood around the gene names being queried. Default: 1
- **database_filter** (*Optional[list]*) – A list of database identifiers to which the query is restricted. Examples: ['reactome'], ['biogrid', 'pid', 'psp'] If not given, all databases are used in the query. For a full list of databases see http://www.pathwaycommons.org/pc2/datasources

Returns **bp** – A BiopaxProcessor containing the obtained BioPAX model in bp.model.

Return type *BiopaxProcessor*

indra.sources.biopax.biopax_api.**process_pc_pathsbetween**(*gene_names*, *neighbor_limit=1*, *database_filter=None*)

Returns a BiopaxProcessor for a PathwayCommons paths-between query.

The paths-between query finds the paths between a set of genes. Here source gene names are given in a single list and all directions of paths between these genes are considered.

http://www.pathwaycommons.org/pc2/#graph

http://www.pathwaycommons.org/pc2/#graph_kind

Parameters

- **gene_names** (*list*) – A list of HGNC gene symbols to search for paths between. Examples: ['BRAF', 'MAP2K1']
- **neighbor_limit** (*Optional[int]*) – The number of steps to limit the length of the paths between the gene names being queried. Default: 1
- **database_filter** (*Optional[list]*) – A list of database identifiers to which the query is restricted. Examples: ['reactome'], ['biogrid', 'pid', 'psp'] If not given, all databases are used in the query. For a full list of databases see http://www.pathwaycommons.org/pc2/datasources

Returns **bp** – A BiopaxProcessor containing the obtained BioPAX model in bp.model.

Return type *BiopaxProcessor*

`indra.sources.biopax.biopax_api.`**`process_pc_pathsfromto`**(*source_genes*, *target_genes*, *neighbor_limit=1*, *database_filter=None*)

Returns a BiopaxProcessor for a PathwayCommons paths-from-to query.

The paths-from-to query finds the paths from a set of source genes to a set of target genes.

http://www.pathwaycommons.org/pc2/#graph

http://www.pathwaycommons.org/pc2/#graph_kind

Parameters

- **source_genes** (*list*) – A list of HGNC gene symbols that are the sources of paths being searched for. Examples: ['BRAF', 'RAF1', 'ARAF']
- **target_genes** (*list*) – A list of HGNC gene symbols that are the targets of paths being searched for. Examples: ['MAP2K1', 'MAP2K2']
- **neighbor_limit** (*Optional[int]*) – The number of steps to limit the length of the paths between the source genes and target genes being queried. Default: 1
- **database_filter** (*Optional[list]*) – A list of database identifiers to which the query is restricted. Examples: ['reactome'], ['biogrid', 'pid', 'psp'] If not given, all databases are used in the query. For a full list of databases see http://www.pathwaycommons.org/pc2/datasources

Returns **bp** – A BiopaxProcessor containing the obtained BioPAX model in bp.model.

Return type *BiopaxProcessor*

Biopax Processor (`indra.sources.biopax.processor`)

class `indra.sources.biopax.processor.`**`BiopaxProcessor`**(*model*)

The BiopaxProcessor extracts INDRA Statements from a BioPAX model.

The BiopaxProcessor uses pattern searches in a BioPAX OWL model to extract mechanisms from which it constructs INDRA Statements.

Parameters **model** (*org.biopax.paxtools.model.Model*) – A BioPAX model object (java object)

model

org.biopax.paxtools.model.Model – A BioPAX model object (java object) which is queried using Paxtools to extract INDRA Statements

statements

list[indra.statements.Statement] – A list of INDRA Statements that were extracted from the model.

get_activity_modification()

Extract INDRA ActiveForm statements from the BioPAX model.

This method extracts ActiveForm Statements that are due to protein modifications. This method reuses the structure of BioPAX Pattern's org.biopax.paxtools.pattern.PatternBox.constrolsStateChange pattern with additional constraints to specify the gain or loss of a modification occurring (phosphorylation, deubiquitination, etc.) and the gain or loss of activity due to the modification state change.

get_complexes()

Extract INDRA Complex Statements from the BioPAX model.

This method searches for org.biopax.paxtools.model.level3.Complex objects which represent molecular complexes. It doesn't reuse BioPAX Pattern's org.biopax.paxtools.pattern.PatternBox.inComplexWith query since that retrieves pairs of complex members rather than the full complex.

get_conversions()
Extract Conversion INDRA Statements from the BioPAX model.

This method uses a custom BioPAX Pattern (one that is not implemented PatternBox) to query for BiochemicalReactions whose left and right hand sides are collections of SmallMolecules. This pattern thereby extracts metabolic conversions as well as signaling processes via small molecules (e.g. lipid phosphorylation or cleavage).

get_gap()
Extract Gap INDRA Statements from the BioPAX model.

This method uses a custom BioPAX Pattern (one that is not implemented PatternBox) to query for controlled BiochemicalReactions in which the same protein is in complex with GTP on the left hand side and in complex with GDP on the right hand side. This implies that the controller is a GAP for the GDP/GTP-bound protein.

get_gef()
Extract Gef INDRA Statements from the BioPAX model.

This method uses a custom BioPAX Pattern (one that is not implemented PatternBox) to query for controlled BiochemicalReactions in which the same protein is in complex with GDP on the left hand side and in complex with GTP on the right hand side. This implies that the controller is a GEF for the GDP/GTP-bound protein.

get_modifications()
Extract INDRA Modification Statements from the BioPAX model.

To extract Modifications, this method reuses the structure of BioPAX Pattern's org.biopax.paxtools.pattern.PatternBox.constrolsStateChange pattern with additional constraints to specify the type of state change occurring (phosphorylation, deubiquitination, etc.).

get_regulate_activities()
Get Activation/Inhibition INDRA Statements from the BioPAX model.

This method extracts Activation/Inhibition Statements and reuses the structure of BioPAX Pattern's org.biopax.paxtools.pattern.PatternBox.constrolsStateChange pattern with additional constraints to specify the gain or loss of activity state but assuring that the activity change is not due to a modification state change (which are extracted by get_modifications and get_activity_modification).

get_regulate_amounts()
Extract INDRA RegulateAmount Statements from the BioPAX model.

This method extracts IncreaseAmount/DecreaseAmount Statements from the BioPAX model. It fully reuses BioPAX Pattern's org.biopax.paxtools.pattern.PatternBox.controlsExpressionWithTemplateReac pattern to find TemplateReactions which control the expression of a protein.

print_statements()
Print all INDRA Statements collected by the processors.

save_model(*file_name=None*)
Save the BioPAX model object in an OWL file.

Parameters **file_name** (*Optional[str]*) – The name of the OWL file to save the model in.

Pathway Commons Client (`indra.sources.biopax.pathway_commons_client`)

`indra.sources.biopax.pathway_commons_client.`**`graph_query`**(*kind*, *source*, *target=None*, *neighbor_limit=1*, *database_filter=None*)

Perform a graph query on PathwayCommons.

For more information on these queries, see http://www.pathwaycommons.org/pc2/#graph

Parameters

- **kind** (*str*) – The kind of graph query to perform. Currently 3 options are implemented, 'neighborhood', 'pathsbetween' and 'pathsfromto'.
- **source** (*list[str]*) – A list of gene names which are the source set for the graph query.
- **target** (*Optional[list[str]]*) – A list of gene names which are the target set for the graph query. Only needed for 'pathsfromto' queries.
- **neighbor_limit** (*Optional[int]*) – This limits the length of the longest path considered in the graph query. Default: 1

Returns **model** – A BioPAX model (java object).

Return type org.biopax.paxtools.model.Model

`indra.sources.biopax.pathway_commons_client.`**`model_to_owl`**(*model*, *fname*)

Save a BioPAX model object as an OWL file.

Parameters

- **model** (*org.biopax.paxtools.model.Model*) – A BioPAX model object (java object).
- **fname** (*str*) – The name of the OWL file to save the model in.

`indra.sources.biopax.pathway_commons_client.`**`owl_str_to_model`**(*owl_str*)

Return a BioPAX model object from an OWL string.

Parameters **owl_str** (*str*) – The model as an OWL string.

Returns **biopax_model** – A BioPAX model object (java object).

Return type org.biopax.paxtools.model.Model

`indra.sources.biopax.pathway_commons_client.`**`owl_to_model`**(*fname*)

Return a BioPAX model object from an OWL file.

Parameters **fname** (*str*) – The name of the OWL file containing the model.

Returns **biopax_model** – A BioPAX model object (java object).

Return type org.biopax.paxtools.model.Model

4.2.3 REACH (`indra.sources.reach`)

REACH API (`indra.sources.reach.reach_api`)

`indra.sources.reach.reach_api.`**`process_json_file`**(*file_name*, *citation=None*)

Return a ReachProcessor by processing the given REACH json file.

The output from the REACH parser is in this json format. This function is useful if the output is saved as a file and needs to be processed. For more information on the format, see: https://github.com/clulab/reach

Parameters

- **file_name** (*str*) – The name of the json file to be processed.
- **citation** (*Optional[str]*) – A PubMed ID passed to be used in the evidence for the extracted INDRA Statements. Default: None

Returns **rp** – A ReachProcessor containing the extracted INDRA Statements in rp.statements.

Return type *ReachProcessor*

indra.sources.reach.reach_api.**process_json_str**(*json_str*, *citation=None*)

Return a ReachProcessor by processing the given REACH json string.

The output from the REACH parser is in this json format. For more information on the format, see: https://github.com/clulab/reach

Parameters

- **json_str** (*str*) – The json string to be processed.
- **citation** (*Optional[str]*) – A PubMed ID passed to be used in the evidence for the extracted INDRA Statements. Default: None

Returns **rp** – A ReachProcessor containing the extracted INDRA Statements in rp.statements.

Return type *ReachProcessor*

indra.sources.reach.reach_api.**process_nxml_file**(*file_name*, *citation=None*, *offline=False*)

Return a ReachProcessor by processing the given NXML file.

NXML is the format used by PubmedCentral for papers in the open access subset.

Parameters

- **file_name** (*str*) – The name of the NXML file to be processed.
- **citation** (*Optional[str]*) – A PubMed ID passed to be used in the evidence for the extracted INDRA Statements. Default: None
- **offline** (*Optional[bool]*) – If set to True, the REACH system is ran offline. Otherwise (by default) the web service is called. Default: False

Returns **rp** – A ReachProcessor containing the extracted INDRA Statements in rp.statements.

Return type *ReachProcessor*

indra.sources.reach.reach_api.**process_nxml_str**(*nxml_str*, *citation=None*, *offline=False*)

Return a ReachProcessor by processing the given NXML string.

NXML is the format used by PubmedCentral for papers in the open access subset.

Parameters

- **nxml_str** (*str*) – The NXML string to be processed.
- **citation** (*Optional[str]*) – A PubMed ID passed to be used in the evidence for the extracted INDRA Statements. Default: None
- **offline** (*Optional[bool]*) – If set to True, the REACH system is ran offline. Otherwise (by default) the web service is called. Default: False

Returns **rp** – A ReachProcessor containing the extracted INDRA Statements in rp.statements.

Return type *ReachProcessor*

`indra.sources.reach.reach_api.`**`process_pmc`**(*pmc_id*, *offline=False*)

Return a ReachProcessor by processing a paper with a given PMC id.

Uses the PMC client to obtain the full text. If it's not available, None is returned.

Parameters

- **`pmc_id`** (`str`) – The ID of a PubmedCentral article. The string may start with PMC but passing just the ID also works. Examples: 3717945, PMC3717945 https://www.ncbi.nlm.nih.gov/pmc/
- **`offline`** (`Optional[bool]`) – If set to True, the REACH system is ran offline. Otherwise (by default) the web service is called. Default: False

Returns **rp** – A ReachProcessor containing the extracted INDRA Statements in rp.statements.

Return type *ReachProcessor*

`indra.sources.reach.reach_api.`**`process_pubmed_abstract`**(*pubmed_id*, *offline=False*)

Return a ReachProcessor by processing an abstract with a given Pubmed id.

Uses the Pubmed client to get the abstract. If that fails, None is returned.

Parameters

- **`pubmed_id`** (`str`) – The ID of a Pubmed article. The string may start with PMID but passing just the ID also works. Examples: 27168024, PMID27168024 https://www.ncbi.nlm.nih.gov/pubmed/
- **`offline`** (`Optional[bool]`) – If set to True, the REACH system is ran offline. Otherwise (by default) the web service is called. Default: False

Returns **rp** – A ReachProcessor containing the extracted INDRA Statements in rp.statements.

Return type *ReachProcessor*

`indra.sources.reach.reach_api.`**`process_text`**(*text*, *citation=None*, *offline=False*)

Return a ReachProcessor by processing the given text.

Parameters

- **`text`** (`str`) – The text to be processed.
- **`citation`** (`Optional[str]`) – A PubMed ID passed to be used in the evidence for the extracted INDRA Statements. This is used when the text to be processed comes from a publication that is not otherwise identified. Default: None
- **`offline`** (`Optional[bool]`) – If set to True, the REACH system is ran offline. Otherwise (by default) the web service is called. Default: False

Returns **rp** – A ReachProcessor containing the extracted INDRA Statements in rp.statements.

Return type *ReachProcessor*

REACH Processor (`indra.sources.reach.processor`)

class `indra.sources.reach.processor.`**`ReachProcessor`**(*json_dict*, *pmid=None*)

The ReachProcessor extracts INDRA Statements from REACH parser output.

Parameters

- **`json_dict`** (`dict`) – A JSON dictionary containing the REACH extractions.
- **`pmid`** (`Optional[str]`) – The PubMed ID associated with the extractions. This can be passed in case the PMID cannot be determined from the extractions alone.‘

tree
objectpath.Tree – The objectpath Tree object representing the extractions.

statements
list[indra.statements.Statement] – A list of INDRA Statements that were extracted by the processor.

citation
str – The PubMed ID associated with the extractions.

all_events
dict[str, str] – The frame IDs of all events by type in the REACH extraction.

get_activation()
Extract INDRA Activation Statements.

get_all_events()
Gather all event IDs in the REACH output by type.

These IDs are stored in the self.all_events dict.

get_complexes()
Extract INDRA Complex Statements.

get_modifications()
Extract Modification INDRA Statements.

get_regulate_amounts()
Extract RegulateAmount INDRA Statements.

get_translocation()
Extract INDRA Translocation Statements.

print_event_statistics()
Print the number of events in the REACH output by type.

REACH reader (indra.sources.reach.reach_reader)

class indra.sources.reach.reach_reader.**ReachReader**
The ReachReader wraps a singleton instance of the REACH reader.

This allows calling the reader many times without having to wait for it to start up each time.

api_ruler
org.clulab.reach.apis.ApiRuler – An instance of the REACH ApiRuler class (java object).

get_api_ruler()
Return the existing reader if it exists or launch a new one.

Returns **api_ruler** – An instance of the REACH ApiRuler class (java object).

Return type org.clulab.reach.apis.ApiRuler

4.2.4 TRIPS (indra.sources.trips)

TRIPS API (indra.sources.trips.trips_api)

indra.sources.trips.trips_api.**process_text**(*text*, *save_xml_name=u'trips_output.xml'*, *save_xml_pretty=True*, *service_endpoint=u'drum'*)
Return a TripsProcessor by processing text.

Parameters

- **text** (*str*) – The text to be processed.
- **save_xml_name** (*Optional[str]*) – The name of the file to save the returned TRIPS extraction knowledge base XML. Default: trips_output.xml
- **save_xml_pretty** (*Optional[bool]*) – If True, the saved XML is pretty-printed. Some third-party tools require non-pretty-printed XMLs which can be obtained by setting this to False. Default: True
- **service_endpoint** (*Optional[str]*) – Selects the TRIPS/DRUM web service endpoint to use. Is a choice between "drum" (default) and "drum-dev", a nightly build.

Returns **tp** – A TripsProcessor containing the extracted INDRA Statements in tp.statements.

Return type *TripsProcessor*

indra.sources.trips.trips_api.**process_xml**(*xml_string*)

Return a TripsProcessor by processing a TRIPS EKB XML string.

Parameters **xml_string** (*str*) – A TRIPS extraction knowledge base (EKB) string to be processed. http://trips.ihmc.us/parser/api.html

Returns **tp** – A TripsProcessor containing the extracted INDRA Statements in tp.statements.

Return type *TripsProcessor*

TRIPS Processor (indra.sources.trips.processor)

class indra.sources.trips.processor.**TripsProcessor**(*xml_string*)

The TripsProcessor extracts INDRA Statements from a TRIPS XML.

For more details on the TRIPS EKB XML format, see http://trips.ihmc.us/parser/cgi/drum

Parameters **xml_string** (*str*) – A TRIPS extraction knowledge base (EKB) in XML format as a string.

tree

xml.etree.ElementTree.Element – An ElementTree object representation of the TRIPS EKB XML.

statements

list[indra.statements.Statement] – A list of INDRA Statements that were extracted from the EKB.

doc_id

str – The PubMed ID of the paper that the extractions are from.

sentences

dict[str: str] – The list of all sentences in the EKB with their IDs

paragraphs

dict[str: str] – The list of all paragraphs in the EKB with their IDs

par_to_sec

dict[str: str] – A map from paragraph IDs to their associated section types

extracted_events

list[xml.etree.ElementTree.Element] – A list of Event elements that have been extracted as INDRA Statements.

get_activations()

Extract direct Activation INDRA Statements.

get_activations_causal()
Extract causal Activation INDRA Statements.

get_activations_stimulate()
Extract Activation INDRA Statements via stimulation.

get_active_forms()
Extract ActiveForm INDRA Statements.

get_active_forms_state()
Extract ActiveForm INDRA Statements.

get_all_events()
Make a list of all events in the TRIPS EKB.

The events are stored in self.all_events.

get_complexes()
Extract Complex INDRA Statements.

get_degradations()
Extract Degradation INDRA Statements.

get_modifications()
Extract all types of Modification INDRA Statements.

get_regulate_amounts()
Extract Increase/DecreaseAmount Statements.

get_syntheses()
Extract IncreaseAmount INDRA Statements.

TRIPS Client (indra.sources.trips.trips_client)

indra.sources.trips.trips_client.**get_xml**(*html*)
Extract the EKB XML from the HTML output of the TRIPS web service.

Parameters **html** (*str*) – The HTML output from the TRIPS web service.

Returns

- *The extraction knowledge base (EKB) XML that contains the event and term*
- *extractions.*

indra.sources.trips.trips_client.**save_xml**(*xml_str*, *file_name*, *pretty=True*)
Save the TRIPS EKB XML in a file.

Parameters

- **xml_str** (*str*) – The TRIPS EKB XML string to be saved.
- **file_name** (*str*) – The name of the file to save the result in.
- **pretty** (*Optional[bool]*) – If True, the XML is pretty printed.

indra.sources.trips.trips_client.**send_query**(*text*, *service_endpoint=u'drum'*, *query_args=None*)
Send a query to the TRIPS web service.

Parameters

- **text** (*str*) – The text to be processed.

- **service_endpoint** (*Optional[str]*) – Selects the TRIPS/DRUM web service endpoint to use. Is a choice between "drum" (default) and "drum-dev", a nightly build.
- **query_args** (*Optional[dict]*) – A dictionary of arguments to be passed with the query.

Returns **html** – The HTML result returned by the web service.

Return type str

4.2.5 NDEx CX API (indra.sources.ndex_cx_api)

indra.sources.ndex_cx.ndex_cx_api.**process_cx**(*cx_json*)
Process a CX JSON object into Statements.

Parameters **cx_json** (*list*) – CX JSON object.

Returns Processor containing Statements.

Return type *NdexCxProcessor*

indra.sources.ndex_cx.ndex_cx_api.**process_cx_file**(*file_name*)
Process a CX JSON file into Statements.

Parameters **file_name** (*str*) – Path to file containing CX JSON.

Returns Processor containing Statements.

Return type *NdexCxProcessor*

indra.sources.ndex_cx.ndex_cx_api.**process_ndex_network**(*network_id, username=None, password=None*)
Process an NDEx network into Statements.

Parameters

- **network_id** (*str*) – NDEx network ID.
- **username** (*str*) – NDEx username.
- **password** (*str*) – NDEx password.

Returns Processor containing Statements. Returns None if there if the HTTP status code indicates an unsuccessful request.

Return type *NdexCxProcessor*

NDEx CX Processor (indra.sources.ndex_cx.processor)

class indra.sources.ndex_cx.processor.**NdexCxProcessor**(*cx*)
The NdexCxProcessor extracts INDRA Statements from Cytoscape CX JSON.

Parameters **cx** (*list of dicts*) – JSON content containing the Cytoscape network in CX format.

statements
list – A list of extracted INDRA Statements. Not all edges in the network may be converted into Statements.

get_agents()
Get list of grounded nodes in the network as Agents.

Returns Only nodes containing sufficient information to be grounded will be contained in this list.

Return type list of Agents

get_node_names()
Get list of all nodes in the network by name.

get_pmids()
Get list of all PMIDs associated with edges in the network.

get_statements()
Convert network edges into Statements.

Returns Converted INDRA Statements.

Return type list of Statements

4.3 Database clients (indra.databases)

4.3.1 HGNC client (indra.hgnc_client)

indra.databases.hgnc_client.**get_entrez_id**(*hgnc_id*)
Return the Entrez ID corresponding to the given HGNC ID.

Parameters **hgnc_id** (*str*) – The HGNC ID to be converted. Note that the HGNC ID is a number that is passed as a string. It is not the same as the HGNC gene symbol.

Returns **entrez_id** – The Entrez ID corresponding to the given HGNC ID.

Return type str

indra.databases.hgnc_client.**get_hgnc_from_entrez**(*entrez_id*)
Return the HGNC ID corresponding to the given Entrez ID.

Parameters **entrez_id** (*str*) – The EntrezC ID to be converted, a number passed as a strig.

Returns **hgnc_id** – The HGNC ID corresponding to the given Entrez ID.

Return type str

indra.databases.hgnc_client.**get_hgnc_from_mouse**(*mgi_id*)
Return the HGNC ID corresponding to the given MGI mouse gene ID.

Parameters **mgi_id** (*str*) – The MGI ID to be converted. Example: "2444934"

Returns **hgnc_id** – The HGNC ID corresponding to the given MGI ID.

Return type str

indra.databases.hgnc_client.**get_hgnc_from_rat**(*rgd_id*)
Return the HGNC ID corresponding to the given RGD rat gene ID.

Parameters **rgd_id** (*str*) – The RGD ID to be converted. Example: "1564928"

Returns **hgnc_id** – The HGNC ID corresponding to the given RGD ID.

Return type str

indra.databases.hgnc_client.**get_hgnc_id**(*hgnc_name*)
Return the HGNC ID corresponding to the given HGNC symbol.

Parameters **hgnc_name** (*str*) – The HGNC symbol to be converted. Example: BRAF

Returns **hgnc_id** – The HGNC ID corresponding to the given HGNC symbol.

Return type str

`indra.databases.hgnc_client.`**`get_hgnc_name`**(*hgnc_id*)
Return the HGNC symbol corresponding to the given HGNC ID.

Parameters **`hgnc_id`** (*`str`*) – The HGNC ID to be converted.

Returns **hgnc_name** – The HGNC symbol corresponding to the given HGNC ID.

Return type str

`indra.databases.hgnc_client.`**`get_mouse_id`**(*hgnc_id*)
Return the MGI mouse ID corresponding to the given HGNC ID.

Parameters **`hgnc_id`** (*`str`*) – The HGNC ID to be converted. Example: ""

Returns **mgi_id** – The MGI ID corresponding to the given HGNC ID.

Return type str

`indra.databases.hgnc_client.`**`get_rat_id`**(*hgnc_id*)
Return the RGD rat ID corresponding to the given HGNC ID.

Parameters **`hgnc_id`** (*`str`*) – The HGNC ID to be converted. Example: ""

Returns **rgd_id** – The RGD ID corresponding to the given HGNC ID.

Return type str

`indra.databases.hgnc_client.`**`get_uniprot_id`**(*hgnc_id*)
Return the UniProt ID corresponding to the given HGNC ID.

Parameters **`hgnc_id`** (*`str`*) – The HGNC ID to be converted. Note that the HGNC ID is a number that is passed as a string. It is not the same as the HGNC gene symbol.

Returns **uniprot_id** – The UniProt ID corresponding to the given HGNC ID.

Return type str

4.3.2 Uniprot client (`indra.databases.uniprot_client`)

`indra.databases.uniprot_client.`**`get_family_members`**(*family_name*, *human_only=True*)
Return the HGNC gene symbols which are the members of a given family.

Parameters

- **`family_name`** (*`str`*) – Family name to be queried.
- **`human_only`** (*`bool`*) – If True, only human proteins in the family will be returned. Default: True

Returns **gene_names** – The HGNC gene symbols corresponding to the given family.

Return type list

`indra.databases.uniprot_client.`**`get_gene_name`**(*protein_id*, *web_fallback=True*)
Return the gene name for the given UniProt ID.

This is an alternative to get_hgnc_name and is useful when HGNC name is not availabe (for instance, when the organism is not homo sapiens).

Parameters

- **`protein_id`** (*`str`*) – UniProt ID to be mapped.

- **web_fallback** (*Optional[bool]*) – If True and the offline lookup fails, the UniProt web service is used to do the query.

Returns **gene_name** – The gene name corresponding to the given Uniprot ID.

Return type str

indra.databases.uniprot_client.**get_id_from_mgi**(*mgi_id*)
Return the UniProt ID given the MGI ID of a mouse protein.

Parameters **mgi_id** (*str*) – The MGI ID of the mouse protein.

Returns **up_id** – The UniProt ID of the mouse protein.

Return type str

indra.databases.uniprot_client.**get_id_from_mnemonic**(*uniprot_mnemonic*)
Return the UniProt ID for the given UniProt mnemonic.

Parameters **uniprot_mnemonic** (*str*) – UniProt mnemonic to be mapped.

Returns **uniprot_id** – The UniProt ID corresponding to the given Uniprot mnemonic.

Return type str

indra.databases.uniprot_client.**get_id_from_rgd**(*rgd_id*)
Return the UniProt ID given the RGD ID of a rat protein.

Parameters **rgd_id** (*str*) – The RGD ID of the rat protein.

Returns **up_id** – The UniProt ID of the rat protein.

Return type str

indra.databases.uniprot_client.**get_mgi_id**(*protein_id*)
Return the MGI ID given the protein id of a mouse protein.

Parameters **protein_id** (*str*) – UniProt ID of the mouse protein

Returns **mgi_id** – MGI ID of the mouse protein

Return type str

indra.databases.uniprot_client.**get_mnemonic**(*protein_id*, *web_fallback=False*)
Return the UniProt mnemonic for the given UniProt ID.

Parameters

- **protein_id** (*str*) – UniProt ID to be mapped.
- **web_fallback** (*Optional[bool]*) – If True and the offline lookup fails, the UniProt web service is used to do the query.

Returns **mnemonic** – The UniProt mnemonic corresponding to the given Uniprot ID.

Return type str

indra.databases.uniprot_client.**get_mouse_id**(*human_protein_id*)
Return the mouse UniProt ID given a human UniProt ID.

Parameters **human_protein_id** (*str*) – The UniProt ID of a human protein.

Returns **mouse_protein_id** – The UniProt ID of a mouse protein orthologous to the given human protein

Return type str

`indra.databases.uniprot_client.`**`get_primary_id`**`(`*protein_id*`)`
Return a primary entry corresponding to the UniProt ID.

Parameters **`protein_id`** (`str`) – The UniProt ID to map to primary.

Returns **primary_id** – If the given ID is primary, it is returned as is. Othwewise the primary IDs are looked up. If there are multiple primary IDs then the first human one is returned. If there are no human primary IDs then the first primary found is returned.

Return type str

`indra.databases.uniprot_client.`**`get_rat_id`**`(`*human_protein_id*`)`
Return the rat UniProt ID given a human UniProt ID.

Parameters **`human_protein_id`** (`str`) – The UniProt ID of a human protein.

Returns **rat_protein_id** – The UniProt ID of a rat protein orthologous to the given human protein

Return type str

`indra.databases.uniprot_client.`**`get_rgd_id`**`(`*protein_id*`)`
Return the RGD ID given the protein id of a rat protein.

Parameters **`protein_id`** (`str`) – UniProt ID of the rat protein

Returns **rgd_id** – RGD ID of the rat protein

Return type str

`indra.databases.uniprot_client.`**`is_human`**`(`*protein_id*`)`
Return True if the given protein id corresponds to a human protein.

Parameters **`protein_id`** (`str`) – UniProt ID of the protein

Returns

Return type True if the protein_id corresponds to a human protein, otherwise False.

`indra.databases.uniprot_client.`**`is_mouse`**`(`*protein_id*`)`
Return True if the given protein id corresponds to a mouse protein.

Parameters **`protein_id`** (`str`) – UniProt ID of the protein

Returns

Return type True if the protein_id corresponds to a mouse protein, otherwise False.

`indra.databases.uniprot_client.`**`is_rat`**`(`*protein_id*`)`
Return True if the given protein id corresponds to a rat protein.

Parameters **`protein_id`** (`str`) – UniProt ID of the protein

Returns

Return type True if the protein_id corresponds to a rat protein, otherwise False.

`indra.databases.uniprot_client.`**`is_secondary`**`(`*protein_id*`)`
Return True if the UniProt ID corresponds to a secondary accession.

Parameters **`protein_id`** (`str`) – The UniProt ID to check.

Returns

Return type True if it is a secondary accessing entry, False otherwise.

`indra.databases.uniprot_client.`**`verify_location`**`(`*protein_id*, *residue*, *location*`)`
Return True if the residue is at the given location in the UP sequence.

Parameters

- **protein_id** (*str*) – UniProt ID of the protein whose sequence is used as reference.
- **residue** (*str*) – A single character amino acid symbol (Y, S, T, V, etc.)
- **location** (*str*) – The location on the protein sequence (starting at 1) at which the residue should be checked against the reference sequence.

Returns

- *True if the given residue is at the given position in the sequence*
- *corresponding to the given UniProt ID, otherwise False.*

indra.databases.uniprot_client.**verify_modification**(*protein_id*, *residue*, *location=None*)

Return True if the residue at the given location has a known modifiation.

Parameters

- **protein_id** (*str*) – UniProt ID of the protein whose sequence is used as reference.
- **residue** (*str*) – A single character amino acid symbol (Y, S, T, V, etc.)
- **location** (*Optional[str]*) – The location on the protein sequence (starting at 1) at which the modification is checked.

Returns

- *True if the given residue is reported to be modified at the given position*
- *in the sequence corresponding to the given UniProt ID, otherwise False.*
- *If location is not given, we only check if there is any residue of the*
- *given type that is modified.*

4.3.3 ChEBI client (indra.databases.chebi_client)

indra.databases.chebi_client.**get_chebi_id_from_pubchem**(*pubchem_id*)

Return the ChEBI ID corresponding to a given Pubchem ID.

Parameters **pubchem_id** (*str*) – Pubchem ID to be converted.

Returns **chebi_id** – ChEBI ID corresponding to the given Pubchem ID. If the lookup fails, None is returned.

Return type str

indra.databases.chebi_client.**get_pubchem_id**(*chebi_id*)

Return the PubChem ID corresponding to a given ChEBI ID.

Parameters **chebi_id** (*str*) – ChEBI ID to be converted.

Returns **pubchem_id** – PubChem ID corresponding to the given ChEBI ID. If the lookup fails, None is returned.

Return type str

4.3.4 BioGRID client (`indra.databases.biogrid_client`)

`indra.databases.biogrid_client.`**`get_publications`**(*gene_names*, *save_json_name=None*)
Return evidence publications for interaction between the given genes.

Parameters

- **`gene_names`** (*`list[str]`*) – A list of gene names (HGNC symbols) to query interactions between. Currently supports exactly two genes only.
- **`save_json_name`** (*`Optional[str]`*) – A file name to save the raw BioGRID web service output in. By default, the raw output is not saved.

Returns **publications** – A list of Publication objects that provide evidence for interactions between the given list of genes.

Return type list[Publication]

4.3.5 Cell type context client (`indra.databases.context_client`)

`indra.databases.context_client.`**`get_mutations`**(*gene_names*, *cell_types*)
Return protein amino acid changes in given genes and cell types.

Parameters

- **`gene_names`** (*`list`*) – HGNC gene symbols for which mutations are queried.
- **`cell_types`** (*`list`*) – List of cell type names in which mutations are queried. The cell type names follow the CCLE database conventions.

  Example: LOXIMVI_SKIN, BT20_BREAST

Returns **res** – A dictionary keyed by cell line, which contains another dictionary that is keyed by gene name, with a list of amino acid substitutions as values.

Return type dict[dict[list]]

`indra.databases.context_client.`**`get_protein_expression`**(*gene_names*, *cell_types*)
Return the protein expression levels of genes in cell types.

Parameters

- **`gene_names`** (*`list`*) – HGNC gene symbols for which expression levels are queried.
- **`cell_types`** (*`list`*) – List of cell type names in which expression levels are queried. The cell type names follow the CCLE database conventions.

  Example: LOXIMVI_SKIN, BT20_BREAST

Returns **res** – A dictionary keyed by cell line, which contains another dictionary that is keyed by gene name, with estimated protein amounts as values.

Return type dict[dict[float]]

4.3.6 Network relevance client (`indra.databases.relevance_client`)

`indra.databases.relevance_client.`**`get_heat_kernel`**(*network_id*)
Return the identifier of a heat kernel calculated for a given network.

Parameters **`network_id`** (*`str`*) – The UUID of the network in NDEx.

Returns **kernel_id** – The identifier of the heat kernel calculated for the given network.

Return type str

`indra.databases.relevance_client.`**`get_relevant_nodes`**(*network_id*, *query_nodes*)
Return a set of network nodes relevant to a given query set.

A heat diffusion algorithm is used on a pre-computed heat kernel for the given network which starts from the given query nodes. The nodes in the network are ranked according to heat score which is a measure of relevance with respect to the query nodes.

Parameters

- **network_id** (*str*) – The UUID of the network in NDEx.
- **query_nodes** (*list[str]*) – A list of node names with respect to which relevance is queried.

Returns **ranked_entities** – A list containing pairs of node names and their relevance scores.

Return type list[(str, float)]

4.3.7 NDEx client (indra.databases.ndex_client)

`indra.databases.ndex_client.`**`send_request`**(*ndex_service_url*, *params*, *is_json=True*, *use_get=False*)
Send a request to the NDEx server.

Parameters

- **ndex_service_url** (*str*) – The URL of the service to use for the request.
- **params** (*dict*) – A dictionary of parameters to send with the request. Parameter keys differ based on the type of request.
- **is_json** (*bool*) – True if the response is in json format, otherwise it is assumed to be text. Default: False
- **use_get** (*bool*) – True if the request needs to use GET instead of POST.

Returns **res** – Depending on the type of service and the is_json parameter, this function either returns a text string or a json dict.

Return type str

4.3.8 cBio portal client (indra.databases.cbio_client)

`indra.databases.cbio_client.`**`get_cancer_studies`**(*study_filter=None*)
Return a list of cancer study identifiers, optionally filtered.

There are typically multiple studies for a given type of cancer and a filter can be used to constrain the returned list.

Parameters **study_filter** (*Optional[str]*) – A string used to filter the study IDs to return. Example: "paad"

Returns **study_ids** – A list of study IDs. For instance "paad" as a filter would result in a list of study IDs with paad in their name like "paad_icgc", "paad_tcga", etc.

Return type list[str]

`indra.databases.cbio_client.`**`get_cancer_types`**(*cancer_filter=None*)
Return a list of cancer types, optionally filtered.

Parameters **cancer_filter** (*Optional[str]*) – A string used to filter cancer types. Its value is the name or part of the name of a type of cancer. Example: "melanoma", "pancreatic", "non-small cell lung"

Returns **type_ids** – A list of cancer types matching the filter. Example: for cancer_filter="pancreatic", the result includes "panet" (neuro-endocrine) and "paad" (adenocarcinoma)

Return type list[str]

indra.databases.cbio_client.**get_case_lists**(*study_id*)

Return a list of the case set ids for a particular study.

TAKE NOTE the "case_list_id" are the same thing as "case_set_id" Within the data, this string is referred to as a "case_list_id". Within API calls it is referred to as a 'case_set_id'. The documentation does not make this explicitly clear.

Parameters **study_id** (*str*) – The ID of the cBio study. Example: 'cellline_ccle_broad' or 'paad_icgc'

Returns **case_set_ids** – A dict keyed to cases containing a dict keyed to genes containing int

Return type dict[dict[int]]

indra.databases.cbio_client.**get_ccle_cna**(*gene_list*, *cell_lines*)

Return a dict of CNAs in given genes and cell lines from CCLE.

This is a specialized call to get_mutations tailored to CCLE cell lines.

Parameters

- **gene_list** (*list[str]*) – A list of HGNC gene symbols to get mutations in
- **cell_lines** (*list[str]*) – A list of CCLE cell line names to get mutations for.

Returns **profile_data** – A dict keyed to cases containing a dict keyed to genes containing int

Return type dict[dict[int]]

indra.databases.cbio_client.**get_ccle_lines_for_mutation**(*gene*, *amino_acid_change*)

Return cell lines with a given point mutation in a given gene.

Checks which cell lines in CCLE have a particular point mutation in a given gene and return their names in a list.

Parameters

- **gene** (*str*) – The HGNC symbol of the mutated gene in whose product the amino acid change occurs. Example: "BRAF"
- **amino_acid_change** (*str*) – The amino acid change of interest. Example: "V600E"

Returns **cell_lines** – A list of CCLE cell lines in which the given mutation occurs.

Return type list

indra.databases.cbio_client.**get_ccle_mrna**(*gene_list*, *cell_lines*)

Return a dict of mRNA amounts in given genes and cell lines from CCLE.

Parameters

- **gene_list** (*list[str]*) – A list of HGNC gene symbols to get mRNA amounts for.
- **cell_lines** (*list[str]*) – A list of CCLE cell line names to get mRNA amounts for.

Returns **mrna_amounts** – A dict keyed to cell lines containing a dict keyed to genes containing float

Return type dict[dict[float]]

`indra.databases.cbio_client.`**`get_genetic_profiles`**(*study_id*, *profile_filter=None*)

Return all the genetic profiles (data sets) for a given study.

Genetic profiles are different types of data for a given study. For instance the study 'cellline_ccle_broad' has profiles such as 'cellline_ccle_broad_mutations' for mutations, 'cellline_ccle_broad_CNA' for copy number alterations, etc.

Parameters

- **study_id** (*str*) – The ID of the cBio study. Example: 'paad_icgc'
- **profile_filter** (*Optional[str]*) – A string used to filter the profiles to return. Will be one of: - MUTATION - MUTATION_EXTENDED - COPY_NUMBER_ALTERATION - MRNA_EXPRESSION - METHYLATION The genetic profiles can include "mutation", "CNA", "rppa", "methylation", etc.

Returns **genetic_profiles** – A list of genetic profiles available for the given study.

Return type list[str]

`indra.databases.cbio_client.`**`get_mutations`**(*study_id*, *gene_list*, *mutation_type=None*, *case_id=None*)

Return mutations as a list of genes and list of amino acid changes.

Parameters

- **study_id** (*str*) – The ID of the cBio study. Example: 'cellline_ccle_broad' or 'paad_icgc'
- **gene_list** (*list[str]*) – A list of genes with their HGNC symbols. Example: ['BRAF', 'KRAS']
- **mutation_type** (*Optional[str]*) – The type of mutation to filter to. mutation_type can be one of: missense, nonsense, frame_shift_ins, frame_shift_del, splice_site
- **case_id** (*Optional[str]*) – The case ID within the study to filter to.

Returns **mutations** – A tuple of two lists, the first one containing a list of genes, and the second one a list of amino acid changes in those genes.

Return type tuple[list]

`indra.databases.cbio_client.`**`get_mutations_ccle`**(*gene_list*, *cell_lines*, *mutation_type=None*)

Return a dict of mutations in given genes and cell lines from CCLE.

This is a specialized call to get_mutations tailored to CCLE cell lines.

Parameters

- **gene_list** (*list[str]*) – A list of HGNC gene symbols to get mutations in
- **cell_lines** (*list[str]*) – A list of CCLE cell line names to get mutations for.
- **mutation_type** (*Optional[str]*) – The type of mutation to filter to. mutation_type can be one of: missense, nonsense, frame_shift_ins, frame_shift_del, splice_site

Returns

mutations – The result from cBioPortal as a dict in the format {cell_line : {gene : [mutation1, mutation2, ...] }}

Example: {'LOXIMVI_SKIN': {'BRAF': ['V600E', 'I208V']}, 'SKMEL30_SKIN': {'BRAF': ['D287H', 'E275K']}}

Return type dict

indra.databases.cbio_client.**get_num_sequenced**(*study_id*)

Return number of sequenced tumors for given study.

This is useful for calculating mutation statistics in terms of the prevalence of certain mutations within a type of cancer.

Parameters **study_id** (*str*) – The ID of the cBio study. Example: 'paad_icgc'

Returns **num_case** – The number of sequenced tumors in the given study

Return type int

indra.databases.cbio_client.**get_profile_data**(*study_id*, *gene_list*, *profile_filter*, *case_set_filter=None*)

Return dict of cases and genes and their respective values.

Parameters

- **study_id** (*str*) – The ID of the cBio study. Example: 'cellline_ccle_broad' or 'paad_icgc'
- **gene_list** (*list[str]*) – A list of genes with their HGNC symbols. Example: ['BRAF', 'KRAS']
- **profile_filter** (*str*) – A string used to filter the profiles to return. Will be one of: - MUTATION - MUTATION_EXTENDED - COPY_NUMBER_ALTERATION - MRNA_EXPRESSION - METHYLATION
- **case_set_filter** (*Optional[str]*) – A string that specifices which case_set_id to use, based on a complete or partial match. If not provided, will look for study_id + '_all'

Returns **profile_data** – A dict keyed to cases containing a dict keyed to genes containing int

Return type dict[dict[int]]

4.4 Literature clients (indra.literature)

indra.literature.**get_full_text**(*paper_id*, *idtype*, *preferred_content_type=u'text/xml'*)

Return the content and the content type of an article.

This function retreives the content of an article by its PubMed ID, PubMed Central ID, or DOI. It prioritizes full text content when available and returns an abstract from PubMed as a fallback.

Parameters

- **paper_id** (*string*) – ID of the article.
- **idtype** (*'pmid', 'pmcid', or 'doi*) – Type of the ID.
- **preferred_content_type** (*Optional[st]r*) – Preference for full-text format, if available. Can be one of 'text/xml', 'text/plain', 'application/pdf'. Default: 'text/xml'

Returns

- **content** (*str*) – The content of the article.
- **content_type** (*str*) – The content type of the article

`indra.literature.`**`id_lookup`**(*paper_id*, *idtype*)

Take an ID of type PMID, PMCID, or DOI and lookup the other IDs.

If the DOI is not found in Pubmed, try to obtain the DOI by doing a reverse-lookup of the DOI in CrossRef using article metadata.

Parameters

- **paper_id** (*string*) – ID of the article.
- **idtype** (*'pmid', 'pmcid', or 'doi*) – Type of the ID.

Returns **ids** – A dictionary with the following keys: pmid, pmcid and doi.

Return type dict

4.4.1 Pubmed client (indra.literature.pubmed_client)

Search and get metadata for articles in Pubmed.

`indra.literature.pubmed_client.`**`expand_pagination`**(*pages*)

Convert a page number to long form, e.g., from 456-7 to 456-457.

`indra.literature.pubmed_client.`**`get_abstract`**(*pubmed_id*, *prepend_title=True*)

Get the abstract of an article in the Pubmed database.

`indra.literature.pubmed_client.`**`get_metadata_for_ids`**(*pmid_list*, *get_issns_from_nlm=False*)

Get article metadata for up to 200 PMIDs from the Pubmed database.

Parameters

- **pmid_list** (*list of PMIDs as strings*) – Can contain 1-200 PMIDs.
- **get_issns_from_nlm** (*boolean*) – Look up the full list of ISSN number for the journal associated with the article, which helps to match articles to CrossRef search results. Defaults to False, since it slows down performance.

Returns Contains the following fields: 'doi', 'title', 'authors', 'journal_title', 'journal_abbrev', 'journal_nlm_id', 'issn_list', 'page'.

Return type dict

`indra.literature.pubmed_client.`**`get_title`**(*pubmed_id*)

Get the title of an article in the Pubmed database.

4.4.2 Pubmed Central client (indra.literature.pmc_client)

`indra.literature.pmc_client.`**`filter_pmids`**(*pmid_list*, *source_type*)

Filter a list of PMIDs for ones with full text from PMC.

Parameters

- **pmid_list** (*list*) – List of PMIDs to filter.
- **source_type** (*string*) – One of 'fulltext', 'oa_xml', 'oa_txt', or 'auth_xml'.

Returns

Return type list of PMIDs available in the specified source/format type.

`indra.literature.pmc_client.`**`id_lookup`**(*paper_id*, *idtype=None*)
This function takes a Pubmed ID, Pubmed Central ID, or DOI and use the Pubmed ID mapping service and looks up all other IDs from one of these. The IDs are returned in a dictionary.

4.4.3 CrossRef client (`indra.literature.crossref_client`)

`indra.literature.crossref_client.`**`doi_query`**(*pmid*, *search_limit=10*)
Get the DOI for a PMID by matching CrossRef and Pubmed metadata.

Searches CrossRef using the article title and then accepts search hits only if they have a matching journal ISSN and page number with what is obtained from the Pubmed database.

`indra.literature.crossref_client.`**`get_fulltext_links`**(*doi*)
Return a list of links to the full text of an article given its DOI. Each list entry is a dictionary with keys: - URL: the URL to the full text - content-type: e.g. text/xml or text/plain - content-version - intended-application: e.g. text-mining

4.4.4 Elsevier client (`indra.literature.elsevier_client`)

For information on the Elsevier API, see:

- API Specification: http://dev.elsevier.com/api_docs.html
- Authentication: https://dev.elsevier.com/tecdoc_api_authentication.html

`indra.literature.elsevier_client.`**`download_article`**(*doi*)
Download an article in XML format from Elsevier.

`indra.literature.elsevier_client.`**`get_abstract`**(*doi*)
Get the abstract of an article from Elsevier.

`indra.literature.elsevier_client.`**`get_article`**(*doi*, *output=u'txt'*)
Get the full body of an article from Elsevier. There are two output modes: 'txt' strips all xml tags and joins the pieces of text in the main text, while 'xml' simply takes the tag containing the body of the article and returns it as is . In the latter case, downstream code needs to be able to interpret Elsever's XML format.

4.5 Preassembly (`indra.preassembler`)

4.5.1 Preassembler (`indra.preassembler`)

class `indra.preassembler.`**`Preassembler`**(*hierarchies*, *stmts=None*)
De-duplicates statements and arranges them in a specificity hierarchy.

Parameters

- **`hierarchies`** (dict[*`indra.preassembler.hierarchy_manager`*]) – A dictionary of hierarchies with keys such as 'entity' (hierarchy of entities, primarily specifying relationships between genes and their families) and 'modification' pointing to HierarchyManagers
- **`stmts`** (list of *`indra.statements.Statement`* or None) – A set of statements to perform pre-assembly on. If None, statements should be added using the *`add_statements()`* method.

`stmts`
list of *`indra.statements.Statement`* – Starting set of statements for preassembly.

unique_stmts
list of *indra.statements.Statement* – Statements resulting from combining duplicates.

related_stmts
list of *indra.statements.Statement* – Top-level statements after building the refinement hierarchy.

hierarchies
dict[*indra.preassembler.hierarchy_manager*] – A dictionary of hierarchies with keys such as 'entity' and 'modification' pointing to HierarchyManagers

add_statements (*stmts*)
Add to the current list of statements.

Parameters **stmts** (list of *indra.statements.Statement*) – Statements to add to the current list.

static combine_duplicate_stmts (*stmts*)
Combine evidence from duplicate Statements.

Statements are deemed to be duplicates if they have the same key returned by the *matches_key()* method of the Statement class. This generally means that statements must be identical in terms of their arguments and can differ only in their associated *Evidence* objects.

This function keeps the first instance of each set of duplicate statements and merges the lists of Evidence from all of the other statements.

Parameters **stmts** (list of *indra.statements.Statement*) – Set of statements to deduplicate.

Returns Unique statements with accumulated evidence across duplicates.

Return type list of *indra.statements.Statement*

Examples

De-duplicate and combine evidence for two statements differing only in their evidence lists:

```
>>> map2k1 = Agent('MAP2K1')
>>> mapk1 = Agent('MAPK1')
>>> stmt1 = Phosphorylation(map2k1, mapk1, 'T', '185',
... evidence=[Evidence(text='evidence 1')])
>>> stmt2 = Phosphorylation(map2k1, mapk1, 'T', '185',
... evidence=[Evidence(text='evidence 2')])
>>> uniq_stmts = Preassembler.combine_duplicate_stmts([stmt1, stmt2])
>>> uniq_stmts
[Phosphorylation(MAP2K1(), MAPK1(), T, 185)]
>>> sorted([e.text for e in uniq_stmts[0].evidence])
['evidence 1', 'evidence 2']
```

combine_duplicates ()
Combine duplicates among *stmts* and save result in *unique_stmts*.

A wrapper around the static method *combine_duplicate_stmts()*.

combine_related (*return_toplevel=True, poolsize=None, size_cutoff=100*)
Connect related statements based on their refinement relationships.

This function takes as a starting point the unique statements (with duplicates removed) and returns a modified flat list of statements containing only those statements which do not represent a refinement of

other existing statements. In other words, the more general versions of a given statement do not appear at the top level, but instead are listed in the *supports* field of the top-level statements.

If `unique_stmts` has not been initialized with the de-duplicated statements, `combine_duplicates()` is called internally.

After this function is called the attribute `related_stmts` is set as a side-effect.

The procedure for combining statements in this way involves a series of steps:

1. The statements are grouped by type (e.g., Phosphorylation) and each type is iterated over independently.
2. Statements of the same type are then grouped according to their Agents' entity hierarchy component identifiers. For instance, ERK, MAPK1 and MAPK3 are all in the same connected component in the entity hierarchy and therefore all Statements of the same type referencing these entities will be grouped. This grouping assures that relations are only possible within Statement groups and not among groups. For two Statements to be in the same group at this step, the Statements must be the same type and the Agents at each position in the Agent lists must either be in the same hierarchy component, or if they are not in the hierarchy, must have identical entity_matches_keys. Statements with None in one of the Agent list positions are collected separately at this stage.
3. Statements with None at either the first or second position are iterated over. For a statement with a None as the first Agent, the second Agent is examined; then the Statement with None is added to all Statement groups with a corresponding component or entity_matches_key in the second position. The same procedure is performed for Statements with None at the second Agent position.
4. The statements within each group are then compared; if one statement represents a refinement of the other (as defined by the *refinement_of()* method implemented for the Statement), then the more refined statement is added to the *supports* field of the more general statement, and the more general statement is added to the *supported_by* field of the more refined statement.
5. A new flat list of statements is created that contains only those statements that have no *supports* entries (statements containing such entries are not eliminated, because they will be retrievable from the *supported_by* fields of other statements). This list is returned to the caller.

On multi-core machines, the algorithm can be parallelized by setting the poolsize argument to the desired number of worker processes. This feature is only available in Python > 3.4.

Note: Subfamily relationships must be consistent across arguments

For now, we require that merges can only occur if the *isa* relationships are all in the *same direction for all the agents* in a Statement. For example, the two statement groups: *RAF_family* -> *MEK1* and *BRAF* -> *MEK_family* would not be merged, since BRAF *isa* RAF_family, but MEK_family is not a MEK1. In the future this restriction could be revisited.

Parameters

- **`return_toplevel`** (*`Optional[bool]`*) – If True only the top level statements are returned. If False, all statements are returned. Default: True
- **`poolsize`** (*`Optional[int]`*) – The number of worker processes to use to parallelize the comparisons performed by the function. If None (default), no parallelization is performed. NOTE: Parallelization is only available on Python 3.4 and above.
- **`size_cutoff`** (*`Optional[int]`*) – Groups with size_cutoff or more statements are sent to worker processes, while smaller groups are compared in the parent process. Default value is 100. Not relevant when parallelization is not used.

Returns The returned list contains Statements representing the more concrete/refined versions of the Statements involving particular entities. The attribute *related_stmts* is also set to this list. However, if return_toplevel is False then all statements are returned, irrespective of level of specificity. In this case the relationships between statements can be accessed via the supports/supported_by attributes.

Return type list of `indra.statement.Statement`

Examples

A more general statement with no information about a Phosphorylation site is identified as supporting a more specific statement:

```
>>> from indra.preassembler.hierarchy_manager import hierarchies
>>> braf = Agent('BRAF')
>>> map2k1 = Agent('MAP2K1')
>>> st1 = Phosphorylation(braf, map2k1)
>>> st2 = Phosphorylation(braf, map2k1, residue='S')
>>> pa = Preassembler(hierarchies, [st1, st2])
>>> combined_stmts = pa.combine_related()
>>> combined_stmts
[Phosphorylation(BRAF(), MAP2K1(), S)]
>>> combined_stmts[0].supported_by
[Phosphorylation(BRAF(), MAP2K1())]
>>> combined_stmts[0].supported_by[0].supports
[Phosphorylation(BRAF(), MAP2K1(), S)]
```

`indra.preassembler.`**`flatten_evidence`**(*stmts*)

Add evidence from *supporting* stmts to evidence for *supported* stmts.

Parameters **`stmts`** (list of *indra.statements.Statement*) – A list of top-level statements with associated supporting statements resulting from building a statement hierarchy with `combine_related()`.

Returns **stmts** – Statement hierarchy identical to the one passed, but with the evidence lists for each statement now containing all of the evidence associated with the statements they are supported by.

Return type list of *indra.statements.Statement*

Examples

Flattening evidence adds the two pieces of evidence from the supporting statement to the evidence list of the top-level statement:

```
>>> from indra.preassembler.hierarchy_manager import hierarchies
>>> braf = Agent('BRAF')
>>> map2k1 = Agent('MAP2K1')
>>> st1 = Phosphorylation(braf, map2k1,
... evidence=[Evidence(text='foo'), Evidence(text='bar')])
>>> st2 = Phosphorylation(braf, map2k1, residue='S',
... evidence=[Evidence(text='baz'), Evidence(text='bak')])
>>> pa = Preassembler(hierarchies, [st1, st2])
>>> pa.combine_related()
[Phosphorylation(BRAF(), MAP2K1(), S)]
>>> [e.text for e in pa.related_stmts[0].evidence]
```

```
['baz', 'bak']
>>> flattened = flatten_evidence(pa.related_stmts)
>>> sorted([e.text for e in flattened[0].evidence])
['bak', 'bar', 'baz', 'foo']
```

indra.preassembler.**flatten_stmts**(*stmts*)

Return the full set of unique stms in a pre-assembled stmt graph.

The flattened list of of statements returned by this function can be compared to the original set of unique statements to make sure no statements have been lost during the preassembly process.

Parameters **stmts** (list of *indra.statements.Statement*) – A list of top-level statements with associated supporting statements resulting from building a statement hierarchy with `combine_related()`.

Returns **stmts** – List of all statements contained in the hierarchical statement graph.

Return type list of *indra.statements.Statement*

Examples

Calling `combine_related()` on two statements results in one top-level statement; calling *flatten_stmts()* recovers both:

```
>>> from indra.preassembler.hierarchy_manager import hierarchies
>>> braf = Agent('BRAF')
>>> map2k1 = Agent('MAP2K1')
>>> st1 = Phosphorylation(braf, map2k1)
>>> st2 = Phosphorylation(braf, map2k1, residue='S')
>>> pa = Preassembler(hierarchies, [st1, st2])
>>> pa.combine_related()
[Phosphorylation(BRAF(), MAP2K1(), S)]
>>> flattened = flatten_stmts(pa.related_stmts)
>>> flattened.sort(key=lambda x: x.matches_key())
>>> flattened
[Phosphorylation(BRAF(), MAP2K1()), Phosphorylation(BRAF(), MAP2K1(), S)]
```

indra.preassembler.**render_stmt_graph**(*statements*, *agent_style=None*)

Render the statement hierarchy as a pygraphviz graph.

Parameters

- **stmts** (list of *indra.statements.Statement*) – A list of top-level statements with associated supporting statements resulting from building a statement hierarchy with `combine_related()`.
- **agent_style** (*dict or None*) – Dict of attributes specifying the visual properties of nodes. If None, the following default attributes are used:

```
agent_style = {'color': 'lightgray', 'style': 'filled',
               'fontname': 'arial'}
```

Returns Pygraphviz graph with nodes representing statements and edges pointing from supported statements to supported_by statements.

Return type pygraphviz.AGraph

Examples

Pattern for getting statements and rendering as a Graphviz graph:

```
>>> from indra.preassembler.hierarchy_manager import hierarchies
>>> braf = Agent('BRAF')
>>> map2k1 = Agent('MAP2K1')
>>> st1 = Phosphorylation(braf, map2k1)
>>> st2 = Phosphorylation(braf, map2k1, residue='S')
>>> pa = Preassembler(hierarchies, [st1, st2])
>>> pa.combine_related()
[Phosphorylation(BRAF(), MAP2K1(), S)]
>>> graph = render_stmt_graph(pa.related_stmts)
>>> graph.write('example_graph.dot') # To make the DOT file
>>> graph.draw('example_graph.png', prog='dot') # To make an image
```

Resulting graph:

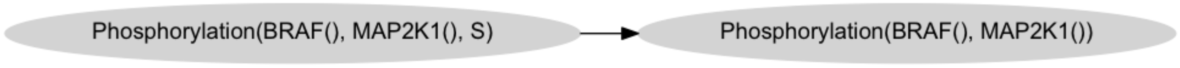

4.5.2 Entity grounding curation and mapping (`indra.preassembler.grounding_mapper`)

`indra.preassembler.grounding_mapper.`**`protein_map_from_twg`**(*twg*)
Build map of entity texts to validated protein grounding.

Looks at the grounding of the entity texts extracted from the statements and finds proteins where there is grounding to a human protein that maps to an HGNC name that is an exact match to the entity text. Returns a dict that can be used to update/expand the grounding map.

4.5.3 Site curation and mapping (`indra.preassembler.sitemapper`)

class `indra.preassembler.sitemapper.`**`MappedStatement`**(*original_stmt*, *mapped_mods*, *mapped_stmt*)
Information about a Statement found to have invalid sites.

Parameters

- **`original_stmt`** (*`indra.statements.Statement`*) – The statement prior to mapping.
- **`mapped_mods`** (*`list of tuples`*) – A list of invalid sites, where each entry in the list has two elements: ((gene_name, residue, position), mapped_site). If the invalid position was not found in the site map, mapped_site is None; otherwise it is a tuple consisting of (residue, position, comment).
- **`mapped_stmt`** (*`indra.statements.Statement`*) – The statement after mapping. Note that if no information was found in the site map, it will be identical to the original statement.

class `indra.preassembler.sitemapper.`**`SiteMapper`**(*site_map*)
Use curated site information to standardize modification sites in stmts.

Parameters **`site_map`** (dict (as returned by *`load_site_map()`*)) – A dict mapping tuples of the form *(gene, orig_res, orig_pos)* to a tuple of the form *(correct_res, correct_pos, comment)*, where *gene* is the string name of the gene (canonicalized to HGNC); *orig_res* and *orig_pos* are the residue and position to be mapped; *correct_res* and *correct_pos* are the corrected residue and

position, and *comment* is a string describing the reason for the mapping (species error, isoform error, wrong residue name, etc.).

Examples

Fixing site errors on both the modification state of an agent (MAP2K1) and the target of a Phosphorylation statement (MAPK1):

```
>>> map2k1_phos = Agent('MAP2K1', db_refs={'UP':'Q02750'}, mods=[
... ModCondition('phosphorylation', 'S', '217'),
... ModCondition('phosphorylation', 'S', '221')])
>>> mapk1 = Agent('MAPK1', db_refs={'UP':'P28482'})
>>> stmt = Phosphorylation(map2k1_phos, mapk1, 'T','183')
>>> (valid, mapped) = default_mapper.map_sites([stmt])
>>> valid
[]
>>> mapped
[
MappedStatement:
    original_stmt: Phosphorylation(MAP2K1(mods: (phosphorylation, S, 217),␣
↪(phosphorylation, S, 221)), MAPK1(), T, 183)
    mapped_mods: (('MAP2K1', 'S', '217'), ('S', '218', 'off by one'))
                 (('MAP2K1', 'S', '221'), ('S', '222', 'off by one'))
                 (('MAPK1', 'T', '183'), ('T', '185', 'off by two; mouse sequence
↪'))
    mapped_stmt: Phosphorylation(MAP2K1(mods: (phosphorylation, S, 218),␣
↪(phosphorylation, S, 222)), MAPK1(), T, 185)
]
>>> ms = mapped[0]
>>> ms.original_stmt
Phosphorylation(MAP2K1(mods: (phosphorylation, S, 217), (phosphorylation, S,␣
↪221)), MAPK1(), T, 183)
>>> ms.mapped_mods
[(('MAP2K1', 'S', '217'), ('S', '218', 'off by one')), (('MAP2K1', 'S', '221'), (
↪'S', '222', 'off by one')), (('MAPK1', 'T', '183'), ('T', '185', 'off by two;␣
↪mouse sequence'))]
>>> ms.mapped_stmt
Phosphorylation(MAP2K1(mods: (phosphorylation, S, 218), (phosphorylation, S,␣
↪222)), MAPK1(), T, 185)
```

map_sites (*stmts*, *do_methionine_offset=True*, *do_orthology_mapping=True*, *do_isoform_mapping=True*)

Check a set of statements for invalid modification sites.

Statements are checked against Uniprot reference sequences to determine if residues referred to by post-translational modifications exist at the given positions.

If there is nothing amiss with a statement (modifications on any of the agents, modifications made in the statement, etc.), then the statement goes into the list of valid statements. If there is a problem with the statement, the offending modifications are looked up in the site map (`site_map`), and an instance of *MappedStatement* is added to the list of mapped statements.

Parameters

- **stmts** (list of `indra.statement.Statement`) – The statements to check for site errors.
- **do_methionine_offset** (*boolean*) – Whether to check for off-by-one errors in site

position (possibly) attributable to site numbering from mature proteins after cleavage of the initial methionine. If True, checks the reference sequence for a known modification at 1 site position greater than the given one; if there exists such a site, creates the mapping. Default is True.

- **do_orthology_mapping** (*boolean*) – Whether to check sequence positions for known modification sites in mouse or rat sequences (based on PhosphoSitePlus data). If a mouse/rat site is found that is linked to a site in the human reference sequence, a mapping is created. Default is True.
- **do_isoform_mapping** (*boolean*) – Whether to check sequence positions for known modifications in other human isoforms of the protein (based on PhosphoSitePlus data). If a site is found that is linked to a site in the human reference sequence, a mapping is created. Default is True.

Returns 2-tuple containing (valid_statements, mapped_statements). The first element of the tuple is a list valid statements (`indra.statement.Statement`) that were not found to contain any site errors. The second element of the tuple is a list of mapped statements (*MappedStatement*) with information on the incorrect sites and corresponding statements with correctly mapped sites.

Return type tuple

`indra.preassembler.sitemapper.`**`default_mapper`** = **<indra.preassembler.sitemapper.SiteMapper object>**

A default instance of *SiteMapper* that contains the site information found in resources/curated_site_map.csv'.

`indra.preassembler.sitemapper.`**`load_site_map`**(*path*)

Load the modification site map from a file.

The site map file should be a comma-separated file with six columns:

```
Gene: HGNC gene name
OrigRes: Original (incorrect) residue
OrigPos: Original (incorrect) residue position
CorrectRes: The correct residue for the modification
CorrectPos: The correct residue position
Comment: Description of the reason for the error.
```

Parameters **path** (*string*) – Path to the tab-separated site map file.

Returns A dict mapping tuples of the form *(gene, orig_res, orig_pos)* to a tuple of the form *(correct_res, correct_pos, comment)*, where *gene* is the string name of the gene (canonicalized to HGNC); *orig_res* and *orig_pos* are the residue and position to be mapped; *correct_res* and *correct_pos* are the corrected residue and position, and *comment* is a string describing the reason for the mapping (species error, isoform error, wrong residue name, etc.).

Return type dict

4.5.4 Hierarchy manager (indra.preassembler.hierarchy_manager)

class `indra.preassembler.hierarchy_manager.`**`HierarchyManager`**(*rdf_file*, *build_closure=True*, *uri_as_name=True*)

Store hierarchical relationships between different types of entities.

Used to store, e.g., entity hierarchies (proteins and protein families) and modification hierarchies (serine phosphorylation vs. phosphorylation).

Parameters

- **rdf_file** (*string*) – Path to the RDF file containing the hierarchy.
- **build_closure** (*Optional[bool]*) – If True, the transitive closure of the hierarchy is generated up from to speed up processing. Default: True
- **uri_as_name** (*Optional[bool]*) – If True, entries are accessed directly by their URIs. If False entries are accessed by finding their name through the hasName relationship. Default: True

graph
instance of *rdflib.Graph* – The RDF graph containing the hierarchy.

build_transitive_closures()
Build the transitive closures of the hierarchy.

This method constructs dictionaries which contain terms in the hierarchy as keys and either all the "isa+" or "partof+" related terms as values.

get_children(*uri*)
Return all (not just immediate) children of a given entry.

Parameters uri (*str*) – The URI of the entry whose children are to be returned. See the get_uri method to construct this URI from a name space and id.

get_parents(*uri*, *type=u'all'*)
Return parents of a given entry.

Parameters

- **uri** (*str*) – The URI of the entry whose parents are to be returned. See the get_uri method to construct this URI from a name space and id.
- **type** (*str*) – 'all': return all parents irrespective of level; 'immediate': return only the immediate parents; 'top': return only the highest level parents

isa(*ns1*, *id1*, *ns2*, *id2*)
Indicate whether one entity has an "isa" relationship to another.

Parameters

- **ns1** (*string*) – Namespace code for an entity.
- **id1** (*string*) – URI for an entity.
- **ns2** (*string*) – Namespace code for an entity.
- **id2** (*string*) – URI for an entity.

Returns True if t1 has an "isa" relationship with t2, either directly or through a series of intermediates; False otherwise.

Return type bool

partof(*ns1*, *id1*, *ns2*, *id2*)
Indicate whether one entity is physically part of another.

Parameters

- **ns1** (*string*) – Namespace code for an entity.
- **id1** (*string*) – URI for an entity.
- **ns2** (*string*) – Namespace code for an entity.
- **id2** (*string*) – URI for an entity.

Returns True if t1 has a "partof" relationship with t2, either directly or through a series of intermediates; False otherwise.

Return type bool

4.6 Belief Engine (`indra.belief`)

class `indra.belief.`**`BeliefEngine`**(*prior_probs=None*)

Assigns beliefs to INDRA Statements based on supporting evidence.

Parameters **`prior_probs`** (*`Optional[dict[dict]]`*) – A dictionary of prior probabilities used to override/extend the default ones. There are two types of prior probabilities: rand and syst corresponding to random error and systematic error rate for each knowledge source. The prior_probs dictionary has the general structure {'rand': {'s1': pr1, ..., 'sn': prn}, 'syst': {'s1': ps1, ..., 'sn': psn}} where 's1' ... 'sn' are names of input sources and pr1 ... prn and ps1 ... psn are error probabilities. Examples: {'rand': {'some_source': 0.1}} sets the random error rate for some_source to 0.1; {'rand': {''}}

`prior_probs`

dict[dict] – A dictionary of prior systematic and random error probabilities for each knowledge source.

`set_hierarchy_probs`(*statements*)

Sets hierarchical belief probabilities for a list of INDRA Statements.

The Statements are assumed to be in a hierarchical relation graph with the supports and supported_by attribute of each Statement object having been set. The hierarchical belief probability of each Statement is calculated based on its prior probability and the probabilities propagated from Statements supporting it in the hierarchy graph.

Parameters **`statements`** (*`list[indra.statements.Statement]`*) – A list of INDRA Statements whose belief scores are to be calculated. Each Statement object's belief attribute is updated by this function.

`set_linked_probs`(*linked_statements*)

Sets the belief probabilities for a list of linked INDRA Statements.

The list of LinkedStatement objects is assumed to come from the MechanismLinker. The belief probability of the inferred Statement is assigned the joint probability of its source Statements.

Parameters **`linked_statements`**(*`list[indra.mechlinker.LinkedStatement]`*) – A list of INDRA LinkedStatements whose belief scores are to be calculated. The belief attribute of the inferred Statement in the LinkedStatement object is updated by this function.

`set_prior_probs`(*statements*)

Sets the prior belief probabilities for a list of INDRA Statements.

The Statements are assumed to be de-duplicated. In other words, each Statement in the list passed to this function is assumed to have a list of Evidence objects that support it. The prior probability of each Statement is calculated based on the number of Evidences it has and their sources.

Parameters **`statements`** (*`list[indra.statements.Statement]`*) – A list of INDRA Statements whose belief scores are to be calculated. Each Statement object's belief attribute is updated by this function.

4.7 Mechanism Linker (`indra.mechlinker`)

class `indra.mechlinker.`**`AgentState`**(*agent*)
A class representing Agent state without identifying a specific Agent.

`bound_conditions`
list[indra.statements.BoundCondition]

`mods`
list[indra.statements.ModCondition]

`mutations`
list[indra.statements.Mutation]

`location`
indra.statements.location

`apply_to`(*agent*)
Apply this object's state to an Agent.

Parameters **`agent`** (`indra.statements.Agent`) – The agent to which the state should be applied

class `indra.mechlinker.`**`BaseAgent`**(*name*)
Represents all activity types and active forms of an Agent.

Parameters

- **`name`** (*`str`*) – The name of the BaseAgent
- **`activity_types`** (*`list[str]`*) – A list of activity types that the Agent has
- **`active_states`** (*`dict`*) – A dict of activity types and their associated Agent states
- **`activity_reductions`** (*`dict`*) – A dict of activity types and the type they are reduced to by inference.

class `indra.mechlinker.`**`BaseAgentSet`**
Container for a set of BaseAgents.

This class wraps a dict of BaseAgent instance and can be used to get and set BaseAgents.

`get_create_base_agent`(*agent*)
Return BaseAgent from an Agent, creating it if needed.

Parameters **`agent`** (`indra.statements.Agent`) –

Returns **base_agent**

Return type *indra.mechlinker.BaseAgent*

class `indra.mechlinker.`**`LinkedStatement`**(*source_stmts*, *inferred_stmt*)
A tuple containing a list of source Statements and an inferred Statement.

The list of source Statements are the basis for the inferred Statement.

Parameters

- **`source_stmts`** (*`list[indra.statements.Statement]`*) – A list of source Statements
- **`inferred_stmts`** (`indra.statements.Statement`) – A Statement that was inferred from the source Statements.

class `indra.mechlinker.`**`MechLinker`**(*stmts=None*)

Rewrite the activation pattern of Statements and derive new Statements.

The mechanism linker (MechLinker) traverses a corpus of Statements and uses various inference steps to make the activity types and active forms consistent among Statements.

`add_statements`(*stmts*)

Add statements to the MechLinker.

Parameters **`stmts`** (*`list[indra.statements.Statement]`*) – A list of Statements to add.

`gather_explicit_activities`()

Aggregate all explicit activities and active forms of Agents.

This function iterates over self.statements and extracts explicitly stated activity types and active forms for Agents.

`gather_implicit_activities`()

Aggregate all implicit activities and active forms of Agents.

Iterate over self.statements and collect the implied activities and active forms of Agents that appear in the Statements.

Note that using this function to collect implied Agent activities can be risky. Assume, for instance, that a Statement from a reading system states that EGF bound to EGFR phosphorylates ERK. This would be interpreted as implicit evidence for the EGFR-bound form of EGF to have 'kinase' activity, which is clearly incorrect.

In contrast the alternative pair of this function: gather_explicit_activities collects only explicitly stated activities.

static **`infer_activations`**(*stmts*)

Return inferred RegulateActivity from Modification + ActiveForm.

This function looks for combinations of Modification and ActiveForm Statements and infers Activation/Inhibition Statements from them. For example, if we know that A phosphorylates B, and the phosphorylated form of B is active, then we can infer that A activates B. This can also be viewed as having "explained" a given Activation/Inhibition Statement with a combination of more mechanistic Modification + ActiveForm Statements.

Parameters **`stmts`** (*`list[indra.statements.Statement]`*) – A list of Statements to infer RegulateActivity from.

Returns **linked_stmts** – A list of LinkedStatements representing the inferred Statements.

Return type list[indra.mechlinker.LinkedStatement]

static **`infer_active_forms`**(*stmts*)

Return inferred ActiveForm from RegulateActivity + Modification.

This function looks for combinations of Activation/Inhibition Statements and Modification Statements, and infers an ActiveForm from them. For example, if we know that A activates B and A phosphorylates B, then we can infer that the phosphorylated form of B is active.

Parameters **`stmts`** (*`list[indra.statements.Statement]`*) – A list of Statements to infer ActiveForms from.

Returns **linked_stmts** – A list of LinkedStatements representing the inferred Statements.

Return type list[indra.mechlinker.LinkedStatement]

static **`infer_complexes`**(*stmts*)

Return inferred Complex from Statements implying physical interaction.

Parameters **stmts** (*list[indra.statements.Statement]*) – A list of Statements to infer Complexes from.

Returns **linked_stmts** – A list of LinkedStatements representing the inferred Statements.

Return type list[indra.mechlinker.LinkedStatement]

static infer_modifications(*stmts*)

Return inferred Modification from RegulateActivity + ActiveForm.

This function looks for combinations of Activation/Inhibition Statements and ActiveForm Statements that imply a Modification Statement. For example, if we know that A activates B, and phosphorylated B is active, then we can infer that A leads to the phosphorylation of B. An additional requirement when making this assumption is that the activity of B should only be dependent on the modified state and not other context - otherwise the inferred Modification is not necessarily warranted.

Parameters **stmts** (*list[indra.statements.Statement]*) – A list of Statements to infer Modifications from.

Returns **linked_stmts** – A list of LinkedStatements representing the inferred Statements.

Return type list[indra.mechlinker.LinkedStatement]

reduce_activities()

Rewrite the activity types referenced in Statements for consistency.

Activity types are reduced to the most specific form whenever possible. For instance, if 'kinase' is the only specific activity type known for the BaseAgent of BRAF, its generic 'activity' forms are rewritten to 'kinase'.

replace_activations(*linked_stmts=None*)

Remove RegulateActivity Statements that can be inferred out.

This function iterates over self.statements and looks for RegulateActivity Statements that either match or are refined by inferred RegulateActivity Statements that were linked (provided as the linked_stmts argument). It removes RegulateActivity Statements from self.statements that can be explained by the linked statements.

Parameters **linked_stmts** (*Optional[list[indra.mechlinker.LinkedStatement]]*) – A list of linked statements, optionally passed from outside. If None is passed, the MechLinker runs self.infer_activations to infer RegulateActivities and obtain a list of LinkedStatements that are then used for removing existing Complexes in self.statements.

replace_complexes(*linked_stmts=None*)

Remove Complex Statements that can be inferred out.

This function iterates over self.statements and looks for Complex Statements that either match or are refined by inferred Complex Statements that were linked (provided as the linked_stmts argument). It removes Complex Statements from self.statements that can be explained by the linked statements.

Parameters **linked_stmts** (*Optional[list[indra.mechlinker.LinkedStatement]]*) – A list of linked statements, optionally passed from outside. If None is passed, the MechLinker runs self.infer_complexes to infer Complexes and obtain a list of LinkedStatements that are then used for removing existing Complexes in self.statements.

require_active_forms()

Rewrites Statements with Agents' active forms in active positions.

As an example, the enzyme in a Modification Statement can be expected to be in an active state. Similarly, subjects of RegulateAmount and RegulateActivity Statements can be expected to be in an active form. This function takes the collected active states of Agents in their corresponding BaseAgents and then rewrites other Statements to apply the active Agent states to them.

Returns **new_stmts** – A list of Statements which includes the newly rewritten Statements. This list is also set as the internal Statement list of the MechLinker.

Return type list[indra.statements.Statement]

4.8 Assemblers of model output (`indra.assemblers`)

4.8.1 Executable PySB models (`indra.assemblers.pysb_assembler`)

class `indra.assemblers.pysb_assembler.`**`PysbAssembler`**(*policies=None*)

Assembler creating a PySB model from a set of INDRA Statements.

Parameters **`policies`** (`Optional[Union[str, dict]]`) – A string or dictionary that defines one or more assembly policies.

If policies is a string, it defines a global assembly policy that applies to all Statement types. Example: contact_only, one_step

A dictionary of policies has keys corresponding to Statement types and values to the policy to be applied to that type of Statement. For Statement types whose policy is undefined, the 'default' policy is applied. Example: {'Phosphorylation': 'two_step'}

`policies`

dict – A dictionary of policies that defines assembly policies for Statement types. It is assigned in the constructor.

`statements`

list – A list of INDRA statements to be assembled.

`model`

pysb.Model – A PySB model object that is assembled by this class.

`agent_set`

_BaseAgentSet – A set of BaseAgents used during the assembly process.

`add_default_initial_conditions`(*value=None*)

Set default initial conditions in the PySB model.

Parameters **`value`** (`Optional[float]`) – Optionally a value can be supplied which will be the initial amount applied. Otherwise a built-in default is used.

`add_statements`(*stmts*)

Add INDRA Statements to the assembler's list of statements.

Parameters **`stmts`** (`list[indra.statements.Statement]`) – A list of `indra.statements.Statement` to be added to the statement list of the assembler.

`export_model`(*format, file_name=None*)

Save the assembled model in a modeling formalism other than PySB.

For more details on exporting PySB models, see http://pysb.readthedocs.io/en/latest/modules/export/index.html

Parameters

- **`format`** (`str`) – The format to export into, for instance "kappa", "bngl", "sbml", "matlab", "mathematica", "potterswheel". See http://pysb.readthedocs.io/en/latest/modules/export/index.html for a list of supported formats.

- **file_name** (*Optional[str]*) – An optional file name to save the exported model into.

Returns **exp_str** – The exported model string

Return type str

make_model (*policies=None*, *initial_conditions=True*, *reverse_effects=False*)
Assemble the PySB model from the collected INDRA Statements.

This method assembles a PySB model from the set of INDRA Statements. The assembled model is both returned and set as the assembler's model argument.

Parameters

- **policies** (*Optional[Union[str, dict]]*) – A string or dictionary of policies, as defined in `indra.assemblers.PysbAssembler`. This set of policies locally supersedes the default setting in the assembler. This is useful when this function is called multiple times with different policies.
- **initial_conditions** (*Optional[bool]*) – If True, default initial conditions are generated for the Monomers in the model.

Returns **model** – The assembled PySB model object.

Return type pysb.Model

print_model ()
Print the assembled model as a PySB program string.

This function is useful when the model needs to be passed as a string to another component.

save_model (*file_name=u'pysb_model.py'*)
Save the assembled model as a PySB program file.

Parameters **file_name** (*Optional[str]*) – The name of the file to save the model program code in. Default: pysb-model.py

save_rst (*file_name=u'pysb_model.rst'*, *module_name=u'pysb_module'*)
Save the assembled model as an RST file for literate modeling.

Parameters

- **file_name** (*Optional[str]*) – The name of the file to save the RST in. Default: pysb_model.rst
- **module_name** (*Optional[str]*) – The name of the python function defining the module. Default: pysb_module

set_context (*cell_type*)
Set protein expression data as initial conditions.

This method uses *indra.databases.context_client* to get protein expression levels for a given cell type and set initial conditions for Monomers in the model accordingly.

Parameters

- **cell_type** (*str*) – Cell type name for which expression levels are queried. The cell type name follows the CCLE database conventions.
- **Example** (*LOXIMVI_SKIN, BT20_BREAST*) –

indra.assemblers.pysb_assembler.**add_rule_to_model** (*model*, *rule*, *annotations=None*)
Add a Rule to a PySB model and handle duplicate component errors.

indra.assemblers.pysb_assembler.**complex_monomers_default**(*stmt*, *agent_set*)
In this (very simple) implementation, proteins in a complex are each given site names corresponding to each of the other members of the complex (lower case). So the resulting complex can be "fully connected" in that each member can be bound to all the others.

indra.assemblers.pysb_assembler.**complex_monomers_one_step**(*stmt*, *agent_set*)
In this (very simple) implementation, proteins in a complex are each given site names corresponding to each of the other members of the complex (lower case). So the resulting complex can be "fully connected" in that each member can be bound to all the others.

indra.assemblers.pysb_assembler.**get_agent_rule_str**(*agent*)
Construct a string from an Agent as part of a PySB rule name.

indra.assemblers.pysb_assembler.**get_annotation**(*component*, *db_name*, *db_ref*)
Construct model Annotations for each component.

Annotation formats follow guidelines at http://identifiers.org/.

indra.assemblers.pysb_assembler.**get_binding_site_name**(*agent*)
Return a binding site name from a given agent.

indra.assemblers.pysb_assembler.**get_create_parameter**(*model*, *name*, *value*, *unique=True*)
Return parameter with given name, creating it if needed.

If unique is false and the parameter exists, the value is not changed; if it does not exist, it will be created. If unique is true then upon conflict a number is added to the end of the parameter name.

indra.assemblers.pysb_assembler.**get_mod_site_name**(*mod_type*, *residue*, *position*)
Return site names for a modification.

indra.assemblers.pysb_assembler.**get_monomer_pattern**(*model*, *agent*, *extra_fields=None*)
Construct a PySB MonomerPattern from an Agent.

indra.assemblers.pysb_assembler.**get_site_pattern**(*agent*)
Construct a dictionary of Monomer site states from an Agent.

This crates the mapping to the associated PySB monomer from an INDRA Agent object.

indra.assemblers.pysb_assembler.**get_uncond_agent**(*agent*)
Construct the unconditional state of an Agent.

The unconditional Agent is a copy of the original agent but without any bound conditions and modification conditions. Mutation conditions, however, are preserved since they are static.

indra.assemblers.pysb_assembler.**grounded_monomer_patterns**(*model*, *agent*)
Get monomer patterns for the agent accounting for grounding information.

indra.assemblers.pysb_assembler.**parse_identifiers_url**(*url*)
Parse an identifiers.org URL into (namespace, ID) tuple.

indra.assemblers.pysb_assembler.**set_base_initial_condition**(*model*, *monomer*, *value*)
Set an initial condition for a monomer in its 'default' state.

indra.assemblers.pysb_assembler.**set_extended_initial_condition**(*model*, *monomer=None*, *value=0*)
Set an initial condition for monomers in "modified" state.

This is useful when using downstream analysis that relies on reactions being active in the model. One example is BioNetGen-based reaction network diagram generation.

4.8.2 Cytoscape networks (`indra.assemblers.cx_assembler`)

class `indra.assemblers.cx_assembler.`**`CxAssembler`**(*stmts=None, network_name=u'indra_assembled'*)

This class assembles a CX network from a set of INDRA Statements.

The CX format is an aspect oriented data mode for networks. The format is defined at http://www.home.ndexbio.org/data-model/. The CX format is the standard for NDEx and is compatible with CytoScape via the CyNDEx plugin.

Parameters

- **`stmts`** (*`Optional[list[indra.statements.Statement]]`*) – A list of INDRA Statements to be assembled.
- **`network_name`** (*`Optional[str]`*) – The name of the network to be assembled. Default: indra_assembled

`statements`

list[indra.statements.Statement] – A list of INDRA Statements to be assembled.

`network_name`

str – The name of the network to be assembled.

`cx`

dict – The structure of the CX network that is assembled.

`add_statements`(*stmts*)

Add INDRA Statements to the assembler's list of statements.

Parameters **`stmts`** (*`list[indra.statements.Statement]`*) – A list of *`indra.statements.Statement`* to be added to the statement list of the assembler.

`make_model`(*add_indra_json=True*)

Assemble the CX network from the collected INDRA Statements.

This method assembles a CX network from the set of INDRA Statements. The assembled network is set as the assembler's cx argument.

Parameters **`add_indra_json`** (*`Optional[bool]`*) – If True, the INDRA Statement JSON annotation is added to each edge in the network. Default: True

Returns **cx_str** – The json serialized CX model.

Return type str

`print_cx`(*pretty=True*)

Return the assembled CX network as a json string.

Parameters **`pretty`** (*`bool`*) – If True, the CX string is formatted with indentation (for human viewing) otherwise no indentation is used.

Returns **json_str** – A json formatted string representation of the CX network.

Return type str

`save_model`(*file_name=u'model.cx'*)

Save the assembled CX network in a file.

Parameters **`file_name`** (*`Optional[str]`*) – The name of the file to save the CX network to. Default: model.cx

set_context (*cell_type*)
Set protein expression data and mutational status as node attribute

This method uses *indra.databases.context_client* to get protein expression levels and mutational status for a given cell type and set a node attribute for proteins accordingly.

Parameters **cell_type** (*str*) – Cell type name for which expression levels are queried. The cell type name follows the CCLE database conventions. Example: LOXIMVI_SKIN, BT20_BREAST

upload_model (*ndex_cred*)
Creates a new NDEx network of the assembled CX model.

To upload the assembled CX model to NDEx, you need to have a registered account on NDEx (http://ndexbio.org/) and have the *ndex* python package installed. The uploaded network is private by default.

Parameters **ndex_cred** (*dict*) – A dictionary with the following entries: 'user': NDEx user name 'password': NDEx password

Returns **network_id** – The UUID of the NDEx network that was created by uploading the assembled CX model.

Return type str

4.8.3 Natural language (indra.assemblers.english_assembler)

class indra.assemblers.english_assembler.**EnglishAssembler** (*stmts=None*)
This assembler generates English sentences from INDRA Statements.

Parameters **stmts** (*Optional[list[indra.statements.Statement]]*) – A list of INDRA Statements to be added to the assembler.

statements
list[indra.statements.Statement] – A list of INDRA Statements to assemble.

model
str – The assembled sentences as a single string.

add_statements (*stmts*)
Add INDRA Statements to the assembler's list of statements.

Parameters **stmts** (*list[indra.statements.Statement]*) – A list of *indra.statements.Statement* to be added to the statement list of the assembler.

make_model ()
Assemble text from the set of collected INDRA Statements.

Returns **stmt_strs** – Return the assembled text as unicode string. By default, the text is a single string consisting of one or more sentences with periods at the end.

Return type str

4.8.4 Node-edge graphs (indra.assemblers.graph_assembler)

class indra.assemblers.graph_assembler.**GraphAssembler** (*stmts=None, graph_properties=None, node_properties=None, edge_properties=None*)
The Graph assembler assembles INDRA Statements into a Graphviz node-edge graph.

Parameters

- **stmts** (*Optional[list[indra.statements.Statement]]*) – A list of INDRA Statements to be added to the assembler's list of Statements.
- **graph_properties** (*Optional[dict[str: str]]*) – A dictionary of graphviz graph properties overriding the default ones.
- **node_properties** (*Optional[dict[str: str]]*) – A dictionary of graphviz node properties overriding the default ones.
- **edge_properties** (*Optional[dict[str: str]]*) – A dictionary of graphviz edge properties overriding the default ones.

statements
list[indra.statements.Statement] – A list of INDRA Statements to be assembled.

graph
pygraphviz.AGraph – A pygraphviz graph that is assembled by this assembler.

existing_nodes
list[tuple] – The list of nodes (identified by node key tuples) that are already in the graph.

existing_edges
list[tuple] – The list of edges (identified by edge key tuples) that are already in the graph.

graph_properties
dict[str: str] – A dictionary of graphviz graph properties used for assembly.

node_properties
dict[str: str] – A dictionary of graphviz node properties used for assembly.

edge_properties
dict[str: str] – A dictionary of graphviz edge properties used for assembly. Note that most edge properties are determined based on the type of the edge by the assembler (e.g. color, arrowhead). These settings cannot be directly controlled through the API.

add_statements(*stmts*)
Add a list of statements to be assembled.

Parameters **stmts** (*list[indra.statements.Statement]*) – A list of INDRA Statements to be appended to the assembler's list.

get_string()
Return the assembled graph as a string.

Returns **graph_string** – The assembled graph as a string.

Return type str

make_model()
Assemble the graph from the assembler's list of INDRA Statements.

save_dot(*file_name=u'graph.dot'*)
Save the graph in a graphviz dot file.

Parameters **file_name** (*Optional[str]*) – The name of the file to save the graph dot string to.

save_pdf(*file_name=u'graph.pdf', prog=u'dot'*)
Draw the graph and save as an image or pdf file.

Parameters

- **file_name** (*Optional[str]*) – The name of the file to save the graph as. Default: graph.pdf
- **prog** (*Optional[str]*) – The graphviz program to use for graph layout. Default: dot

4.8.5 SIF / Boolean networks (indra.assemblers.sif_assembler)

class indra.assemblers.sif_assembler.**SifAssembler**(*stmts=None*)

The SIF assembler assembles INDRA Statements into a networkx graph.

This graph can then be exported into SIF (simple ineraction format) or a Boolean network.

Parameters **stmts** (*Optional[list[indra.statements.Statement]]*) – A list of INDRA Statements to be added to the assembler's list of Statements.

graph

networkx.DiGraph – A networkx graph that is assembled by this assembler.

make_model(*use_name_as_key=False, include_mods=False, include_complexes=False*)

Assemble the graph from the assembler's list of INDRA Statements.

Parameters

- **use_name_as_key** (*boolean*) – If True, uses the name of the agent as the key to the nodes in the network. If False (default) uses the matches_key() of the agent.
- **include_mods** (*boolean*) – If True, adds Modification statements into the graph as directed edges. Default is False.
- **include_complexes** (*boolean*) – If True, creates two edges (in both directions) between all pairs of nodes in Complex statements. Default is False.

print_boolean_net(*out_file=None*)

Return a Boolean network from the assembled graph.

See https://github.com/ialbert/booleannet for details about the format used to encode the Boolean rules.

Parameters **out_file** (*Optional[str]*) – A file name in which the Boolean network is saved.

Returns **full_str** – The string representing the Boolean network.

Return type str

print_loopy(*as_url=True*)

Return

Parameters **out_file** (*Optional[str]*) – A file name in which the Loopy network is saved.

Returns **full_str** – The string representing the Loopy network.

Return type str

print_model(*include_unsigned_edges=False*)

Return a SIF string of the assembled model.

Parameters **include_unsigned_edges** (*bool*) – If True, includes edges with an unknown activating/inactivating relationship (e.g., most PTMs). Default is False.

save_model(*fname*)

Save the assembled model's SIF string into a file.

Parameters **fname** (*str*) – The name of the file to save the SIF into.

4.8.6 MITRE "index cards" (`indra.assemblers.index_card_assembler`)

class `indra.assemblers.index_card_assembler.`**`IndexCardAssembler`**(*statements=None*, *pmc_override=None*)

Assembler creating index cards from a set of INDRA Statements.

Parameters

- **`statements`** (*`list`*) – A list of INDRA statements to be assembled.
- **`pmc_override`** (*`Optional[str]`*) – A PMC ID to assign to the index card.

`statements`

list – A list of INDRA statements to be assembled.

`add_statements`(*statements*)

Add statements to the assembler.

Parameters **`statements`** (*`list[indra.statement.Statements]`*) – The list of Statements to add to the assembler.

`make_model`()

Assemble statements into index cards.

`print_model`()

Return the assembled cards as a JSON string.

Returns **cards_json** – The JSON string representing the assembled cards.

Return type str

`save_model`(*file_name=u'index_cards.json'*)

Save the assembled cards into a file.

Parameters **`file_name`** (*`Optional[str]`*) – The name of the file to save the cards into. Default: index_cards.json

4.8.7 SBGN output (`indra.assemblers.sbgn_assembler`)

class `indra.assemblers.sbgn_assembler.`**`SBGNAssembler`**(*statements=None*)

This class assembles an SBGN model from a set of INDRA Statements.

The Systems Biology Graphical Notation (SBGN) is a widely used graphical notation standard for systems biology models. This assembler creates SBGN models following the Process Desctiption (PD) standard, documented at: https://github.com/sbgn/process-descriptions/blob/master/UserManual/sbgn_PD-level1-user-public.pdf. For more information on SBGN, see: http://sbgn.github.io/sbgn/

Parameters **`stmts`** (*`Optional[list[indra.statements.Statement]]`*) – A list of INDRA Statements to be assembled.

`statements`

list[indra.statements.Statement] – A list of INDRA Statements to be assembled.

`sbgn`

lxml.etree.ElementTree – The structure of the SBGN model that is assembled, represented as an XML ElementTree.

`add_statements`(*stmts*)

Add INDRA Statements to the assembler's list of statements.

Parameters **stmts** (*list[indra.statements.Statement]*) – A list of *indra.statements.Statement* to be added to the statement list of the assembler.

make_model()
Assemble the SBGN model from the collected INDRA Statements.

This method assembles an SBGN model from the set of INDRA Statements. The assembled model is set as the assembler's sbgn attribute (it is represented as an XML ElementTree internally). The model is returned as a serialized XML string.

Returns **sbgn_str** – The XML serialized SBGN model.

Return type str

print_model(*pretty=True*, *encoding=u'utf8'*)
Return the assembled SBGN model as an XML string.

Parameters **pretty** (*Optional[bool]*) – If True, the SBGN string is formatted with indentation (for human viewing) otherwise no indentation is used. Default: True

Returns **sbgn_str** – An XML string representation of the SBGN model.

Return type bytes (str in Python 2)

save_model(*file_name=u'model.sbgn'*)
Save the assembled SBGN model in a file.

Parameters **file_name** (*Optional[str]*) – The name of the file to save the SBGN network to. Default: model.sbgn

4.9 Explanation (indra.explanation)

4.9.1 Check whether a rule-based model satisfies a property (indra.explanation.model_checker)

class indra.explanation.model_checker.**ModelChecker**(*model*, *statements=None*, *agent_obs=None*)
Check a PySB model against a set of INDRA statements.

Parameters

- **model** (*pysb.Model*) – A PySB model to check.
- **statements** (*Optional[list[indra.statements.Statement]]*) – A list of INDRA Statements to check the model against.
- **agent_obs** (*Optional[list[indra.statements.Agent]]*) – A list of INDRA Agents in a given state to be observed.

add_statements(*stmts*)
Add to the list of statements to check against the model.

Parameters **stmts** (*list[indra.statements.Statement]*) – The list of Statements to be added for checking.

check_model(*max_paths=1*, *max_path_length=5*)
Check all the statements added to the ModelChecker.

Parameters

- **max_paths** (*Optional[int]*) – The maximum number of specific paths to return for each Statement to be explained. Default: 1
- **max_path_length** (*Optional[int]*) – The maximum length of specific paths to return. Default: 5

Returns Each tuple contains the Statement checked against the model and a PathResult object describing the results of model checking.

Return type list of (Statement, PathResult)

check_statement(*stmt*, *max_paths=1*, *max_path_length=5*)
Check a single Statement against the model.

Parameters

- **stmt** (indra.statements.Statement) – The Statement to check.
- **max_paths** (*Optional[int]*) – The maximum number of specific paths to return for each Statement to be explained. Default: 1
- **max_path_length** (*Optional[int]*) – The maximum length of specific paths to return. Default: 5

Returns True if the model satisfies the Statement.

Return type boolean

get_im(*force_update=False*)
Get the influence map for the model, generating it if necessary.

Parameters **force_update** (*bool*) – Whether to generate the influence map when the function is called. If False, returns the previously generated influence map if available. Defaults to True.

Returns

The influence map can be rendered as a pdf using the dot layout program as follows:

```
influence_map.draw('influence_map.pdf', prog='dot')
```

Return type pygraphviz AGraph object containing the influence map.

class indra.explanation.model_checker.**PathMetric**(*source_node*, *target_node*, *polarity*, *length*)
Describes results of simple path search (path existence).

class indra.explanation.model_checker.**PathResult**(*path_found*, *result_code*, *max_paths*, *max_path_length*)
Describes results of running the ModelChecker on a single Statement.

Parameters

- **path_found** (*bool*) –
- **result_code** (*string*) – STATEMENT_TYPE_NOT_HANDLED SUBJECT_MONOMERS_NOT_FOUND OBSERVABLES_NOT_FOUND NO_PATHS_FOUND MAX_PATH_LENGTH_EXCEEDED PATHS_FOUND INPUT_RULES_NOT_FOUND

path_found
boolean

result_code
string

path_metrics
list of PathMetric

paths
list of paths

max_paths

max_path_length

4.10 Tools (indra.tools)

4.10.1 Run assembly components in a pipeline (indra.tools.assemble_corpus)

indra.tools.assemble_corpus.**dump_statements**(*stmts*, *fname*)
Dump a list of statements into a pickle file.

Parameters **fname** (*str*) – The name of the pickle file to dump statements into.

indra.tools.assemble_corpus.**dump_stmt_strings**(*stmts*, *fname*)
Save printed statements in a file.

Parameters

- **stmts_in** (*list[indra.statements.Statement]*) – A list of statements to save in a text file.
- **fname** (*Optional[str]*) – The name of a text file to save the printed statements into.

indra.tools.assemble_corpus.**expand_families**(*stmts_in*, ***kwargs*)
Expand Bioentities Agents to individual genes.

Parameters

- **stmts_in** (*list[indra.statements.Statement]*) – A list of statements to expand.
- **save** (*Optional[str]*) – The name of a pickle file to save the results (stmts_out) into.

Returns **stmts_out** – A list of expanded statements.

Return type list[indra.statements.Statement]

indra.tools.assemble_corpus.**filter_belief**(*stmts_in*, *belief_cutoff*, ***kwargs*)
Filter to statements with belief above a given cutoff.

Parameters

- **stmts_in** (*list[indra.statements.Statement]*) – A list of statements to filter.
- **belief_cutoff** (*float*) – Only statements with belief above the belief_cutoff will be returned. Here 0 < belief_cutoff < 1.
- **save** (*Optional[str]*) – The name of a pickle file to save the results (stmts_out) into.

Returns **stmts_out** – A list of filtered statements.

Return type list[indra.statements.Statement]

indra.tools.assemble_corpus.**filter_by_type**(*stmts_in*, *stmt_type*, ***kwargs*)
Filter to a given statement type.

Parameters

- **stmts_in** (*list[indra.statements.Statement]*) – A list of statements to filter.
- **stmt_type** (indra.statements.Statement) – The class of the statement type to filter for. Example: indra.statements.Modification
- **invert** (*Optional[bool]*) – If True, the statements that are not of the given type are returned. Default: False
- **save** (*Optional[str]*) – The name of a pickle file to save the results (stmts_out) into.

Returns **stmts_out** – A list of filtered statements.

Return type list[indra.statements.Statement]

`indra.tools.assemble_corpus.`**`filter_direct`**(*stmts_in*, ***kwargs*)

Filter to statements that are direct interactions

Parameters

- **stmts_in** (*list[indra.statements.Statement]*) – A list of statements to filter.
- **save** (*Optional[str]*) – The name of a pickle file to save the results (stmts_out) into.

Returns **stmts_out** – A list of filtered statements.

Return type list[indra.statements.Statement]

`indra.tools.assemble_corpus.`**`filter_enzyme_kinase`**(*stmts_in*, ***kwargs*)

Filter Phosphorylations to ones where the enzyme is a known kinase.

Parameters

- **stmts_in** (*list[indra.statements.Statement]*) – A list of statements to filter.
- **save** (*Optional[str]*) – The name of a pickle file to save the results (stmts_out) into.

Returns **stmts_out** – A list of filtered statements.

Return type list[indra.statements.Statement]

`indra.tools.assemble_corpus.`**`filter_evidence_source`**(*stmts_in*, *source_apis*, *policy=u'one'*, ***kwargs*)

Filter to statements that have evidence from a given set of sources.

Parameters

- **stmts_in** (*list[indra.statements.Statement]*) – A list of statements to filter.
- **source_apis** (*list[str]*) – A list of sources to filter for. Examples: biopax, bel, reach
- **policy** (*Optional[str]*) – If 'one', a statement that hase evidence from any of the sources is kept. If 'all', only those statements are kept which have evidence from all the input sources specified in source_apis. If 'none', only those statements are kept that don't have evidence from any of the sources specified in source_apis.
- **save** (*Optional[str]*) – The name of a pickle file to save the results (stmts_out) into.

Returns **stmts_out** – A list of filtered statements.

Return type list[indra.statements.Statement]

`indra.tools.assemble_corpus.`**`filter_gene_list`**(*stmts_in*, *gene_list*, *policy*, *allow_families=False*, ***kwargs*)

Return statements that contain genes given in a list.

Parameters

- **stmts_in** (*list[indra.statements.Statement]*) – A list of statements to filter.
- **gene_list** (*list[str]*) – A list of gene symbols to filter for.
- **policy** (*str*) – The policy to apply when filtering for the list of genes. "one": keep statements that contain at least one of the list of genes and possibly others not in the list "all": keep statements that only contain genes given in the list
- **allow_families** (*Optional[bool]*) – Will include statements involving Bioentities families containing one of the genes in the gene list. Default: False
- **save** (*Optional[str]*) – The name of a pickle file to save the results (stmts_out) into.

Returns **stmts_out** – A list of filtered statements.

Return type list[indra.statements.Statement]

`indra.tools.assemble_corpus.`**`filter_genes_only`**(*stmts_in*, ***kwargs*)

Filter to statements containing genes only.

Parameters

- **stmts_in** (*list[indra.statements.Statement]*) – A list of statements to filter.
- **specific_only** (*Optional[bool]*) – If True, only elementary genes/proteins will be kept and families will be filtered out. If False, families are also included in the output. Default: False
- **save** (*Optional[str]*) – The name of a pickle file to save the results (stmts_out) into.

Returns **stmts_out** – A list of filtered statements.

Return type list[indra.statements.Statement]

`indra.tools.assemble_corpus.`**`filter_grounded_only`**(*stmts_in*, ***kwargs*)

Filter to statements that have grounded agents.

Parameters

- **stmts_in** (*list[indra.statements.Statement]*) – A list of statements to filter.
- **save** (*Optional[str]*) – The name of a pickle file to save the results (stmts_out) into.

Returns **stmts_out** – A list of filtered statements.

Return type list[indra.statements.Statement]

`indra.tools.assemble_corpus.`**`filter_human_only`**(*stmts_in*, ***kwargs*)

Filter out statements that are not grounded to human genes.

Parameters

- **stmts_in** (*list[indra.statements.Statement]*) – A list of statements to filter.
- **save** (*Optional[str]*) – The name of a pickle file to save the results (stmts_out) into.

Returns **stmts_out** – A list of filtered statements.

Return type list[indra.statements.Statement]

indra.tools.assemble_corpus.**filter_inconsequential_acts**(*stmts_in*, *whitelist=None*, ***kwargs*)

Filter out Activations that modify inconsequential activities

Inconsequential here means that the site is not mentioned / tested in any other statement. In some cases specific activity types should be preserved, for instance, to be used as readouts in a model. In this case, the given activities can be passed in a whitelist.

Parameters

- **stmts_in** (*list[indra.statements.Statement]*) – A list of statements to filter.
- **whitelist** (*Optional[dict]*) – A whitelist containing agent activity types which should be preserved even if no other statement refers to them. The whitelist parameter is a dictionary in which the key is a gene name and the value is a list of activity types. Example: whitelist = {'MAP2K1': ['kinase']}
- **save** (*Optional[str]*) – The name of a pickle file to save the results (stmts_out) into.

Returns **stmts_out** – A list of filtered statements.

Return type list[indra.statements.Statement]

indra.tools.assemble_corpus.**filter_inconsequential_mods**(*stmts_in*, *whitelist=None*, ***kwargs*)

Filter out Modifications that modify inconsequential sites

Inconsequential here means that the site is not mentioned / tested in any other statement. In some cases specific sites should be preserved, for instance, to be used as readouts in a model. In this case, the given sites can be passed in a whitelist.

Parameters

- **stmts_in** (*list[indra.statements.Statement]*) – A list of statements to filter.
- **whitelist** (*Optional[dict]*) – A whitelist containing agent modification sites whose modifications should be preserved even if no other statement refers to them. The whitelist parameter is a dictionary in which the key is a gene name and the value is a list of tuples of (modification_type, residue, position). Example: whitelist = {'MAP2K1': [('phosphorylation', 'S', '222')]}
- **save** (*Optional[str]*) – The name of a pickle file to save the results (stmts_out) into.

Returns **stmts_out** – A list of filtered statements.

Return type list[indra.statements.Statement]

indra.tools.assemble_corpus.**filter_mod_nokinase**(*stmts_in*, ***kwargs*)

Filter non-phospho Modifications to ones with a non-kinase enzyme.

Parameters

- **stmts_in** (*list[indra.statements.Statement]*) – A list of statements to filter.
- **save** (*Optional[str]*) – The name of a pickle file to save the results (stmts_out) into.

Returns **stmts_out** – A list of filtered statements.

Return type list[indra.statements.Statement]

`indra.tools.assemble_corpus.`**`filter_mutation_status`**(*stmts_in*, *mutations*, *deletions*, ***kwargs*)

Filter statements based on existing mutations/deletions

This filter helps to contextualize a set of statements to a given cell type. Given a list of deleted genes, it removes statements that refer to these genes. It also takes a list of mutations and removes statements that refer to mutations not relevant for the given context.

Parameters

- **stmts_in** (*list[indra.statements.Statement]*) – A list of statements to filter.
- **mutations** (*dict*) – A dictionary whose keys are gene names, and the values are lists of tuples of the form (residue_from, position, residue_to). Example: mutations = {'BRAF': [('V', '600', 'E')]}
- **deletions** (*list*) – A list of gene names that are deleted.
- **save** (*Optional[str]*) – The name of a pickle file to save the results (stmts_out) into.

Returns **stmts_out** – A list of filtered statements.

Return type list[indra.statements.Statement]

`indra.tools.assemble_corpus.`**`filter_no_hypothesis`**(*stmts_in*, ***kwargs*)

Filter to statements that are not marked as hypothesis in epistemics.

Parameters

- **stmts_in** (*list[indra.statements.Statement]*) – A list of statements to filter.
- **save** (*Optional[str]*) – The name of a pickle file to save the results (stmts_out) into.

Returns **stmts_out** – A list of filtered statements.

Return type list[indra.statements.Statement]

`indra.tools.assemble_corpus.`**`filter_top_level`**(*stmts_in*, ***kwargs*)

Filter to statements that are at the top-level of the hierarchy.

Here top-level statements correspond to most specific ones.

Parameters

- **stmts_in** (*list[indra.statements.Statement]*) – A list of statements to filter.
- **save** (*Optional[str]*) – The name of a pickle file to save the results (stmts_out) into.

Returns **stmts_out** – A list of filtered statements.

Return type list[indra.statements.Statement]

`indra.tools.assemble_corpus.`**`filter_transcription_factor`**(*stmts_in*, ***kwargs*)

Filter out RegulateAmounts where subject is not a transcription factor.

Parameters

- **stmts_in** (*list[indra.statements.Statement]*) – A list of statements to filter.
- **save** (*Optional[str]*) – The name of a pickle file to save the results (stmts_out) into.

Returns **stmts_out** – A list of filtered statements.

Return type list[indra.statements.Statement]

`indra.tools.assemble_corpus.`**`filter_uuid_list`**(*stmts_in*, *uuids*, ***kwargs*)
Filter to Statements corresponding to given UUIDs

Parameters

- **stmts_in** (*list[indra.statements.Statement]*) – A list of statements to filter.
- **uuids** (*list[str]*) – A list of UUIDs to filter for.
- **save** (*Optional[str]*) – The name of a pickle file to save the results (stmts_out) into.

Returns **stmts_out** – A list of filtered statements.

Return type list[indra.statements.Statement]

`indra.tools.assemble_corpus.`**`load_statements`**(*fname*, *as_dict=False*)
Load statements from a pickle file.

Parameters

- **fname** (*str*) – The name of the pickle file to load statements from.
- **as_dict** (*Optional[bool]*) – If True and the pickle file contains a dictionary of statements, it is returned as a dictionary. If False, the statements are always returned in a list. Default: False

Returns **stmts** – A list or dict of statements that were loaded.

Return type list

`indra.tools.assemble_corpus.`**`map_grounding`**(*stmts_in*, ***kwargs*)
Map grounding using the GroundingMapper.

Parameters

- **stmts_in** (*list[indra.statements.Statement]*) – A list of statements to map.
- **do_rename** (*Optional[bool]*) – If True, Agents are renamed based on their mapped grounding.
- **save** (*Optional[str]*) – The name of a pickle file to save the results (stmts_out) into.

Returns **stmts_out** – A list of mapped statements.

Return type list[indra.statements.Statement]

`indra.tools.assemble_corpus.`**`map_sequence`**(*stmts_in*, ***kwargs*)
Map sequences using the SiteMapper.

Parameters

- **stmts_in** (*list[indra.statements.Statement]*) – A list of statements to map.
- **do_methionine_offset** (*boolean*) – Whether to check for off-by-one errors in site position (possibly) attributable to site numbering from mature proteins after cleavage of the initial methionine. If True, checks the reference sequence for a known modification at 1 site position greater than the given one; if there exists such a site, creates the mapping. Default is True.
- **do_orthology_mapping** (*boolean*) – Whether to check sequence positions for known modification sites in mouse or rat sequences (based on PhosphoSitePlus data). If a mouse/rat site is found that is linked to a site in the human reference sequence, a mapping is created. Default is True.

- **do_isoform_mapping** (*boolean*) – Whether to check sequence positions for known modifications in other human isoforms of the protein (based on PhosphoSitePlus data). If a site is found that is linked to a site in the human reference sequence, a mapping is created. Default is True.
- **save** (*Optional[str]*) – The name of a pickle file to save the results (stmts_out) into.

Returns **stmts_out** – A list of mapped statements.

Return type list[indra.statements.Statement]

indra.tools.assemble_corpus.**reduce_activities**(*stmts_in*, ***kwargs*)

Reduce the activity types in a list of statements

Parameters

- **stmts_in** (*list[indra.statements.Statement]*) – A list of statements to reduce activity types in.
- **save** (*Optional[str]*) – The name of a pickle file to save the results (stmts_out) into.

Returns **stmts_out** – A list of reduced activity statements.

Return type list[indra.statements.Statement]

indra.tools.assemble_corpus.**run_preassembly**(*stmts_in*, ***kwargs*)

Run preassembly on a list of statements.

Parameters

- **stmts_in** (*list[indra.statements.Statement]*) – A list of statements to preassemble.
- **return_toplevel** (*Optional[bool]*) – If True, only the top-level statements are returned. If False, all statements are returned irrespective of level of specificity. Default: True
- **poolsize** (*Optional[int]*) – The number of worker processes to use to parallelize the comparisons performed by the function. If None (default), no parallelization is performed. NOTE: Parallelization is only available on Python 3.4 and above.
- **size_cutoff** (*Optional[int]*) – Groups with size_cutoff or more statements are sent to worker processes, while smaller groups are compared in the parent process. Default value is 100. Not relevant when parallelization is not used.
- **save** (*Optional[str]*) – The name of a pickle file to save the results (stmts_out) into.
- **save_unique** (*Optional[str]*) – The name of a pickle file to save the unique statements into.

Returns **stmts_out** – A list of preassembled top-level statements.

Return type list[indra.statements.Statement]

indra.tools.assemble_corpus.**run_preassembly_duplicate**(*preassembler*, *beliefengine*, ***kwargs*)

Run deduplication stage of preassembly on a list of statements.

Parameters

- **preassembler** (indra.preassembler.Preassembler) – A Preassembler instance
- **beliefengine** (indra.belief.BeliefEngine) – A BeliefEngine instance
- **save** (*Optional[str]*) – The name of a pickle file to save the results (stmts_out) into.

Returns **stmts_out** – A list of unique statements.

Return type list[indra.statements.Statement]

indra.tools.assemble_corpus.**run_preassembly_related**(*preassembler*, *beliefengine*, ***kwargs*)

Run related stage of preassembly on a list of statements.

Parameters

- **preassembler** (indra.preassembler.Preassembler) – A Preassembler instance which already has a set of unique statements internally.
- **beliefengine** (indra.belief.BeliefEngine) – A BeliefEngine instance
- **return_toplevel** (*Optional[bool]*) – If True, only the top-level statements are returned. If False, all statements are returned irrespective of level of specificity. Default: True
- **poolsize** (*Optional[int]*) – The number of worker processes to use to parallelize the comparisons performed by the function. If None (default), no parallelization is performed. NOTE: Parallelization is only available on Python 3.4 and above.
- **size_cutoff** (*Optional[int]*) – Groups with size_cutoff or more statements are sent to worker processes, while smaller groups are compared in the parent process. Default value is 100. Not relevant when parallelization is not used.
- **save** (*Optional[str]*) – The name of a pickle file to save the results (stmts_out) into.

Returns **stmts_out** – A list of preassembled top-level statements.

Return type list[indra.statements.Statement]

indra.tools.assemble_corpus.**strip_agent_context**(*stmts_in*, ***kwargs*)

Strip any context on agents within each statement.

Parameters

- **stmts_in** (*list[indra.statements.Statement]*) – A list of statements whose agent context should be stripped.
- **save** (*Optional[str]*) – The name of a pickle file to save the results (stmts_out) into.

Returns **stmts_out** – A list of stripped statements.

Return type list[indra.statements.Statement]

4.10.2 Build a network from a gene list (indra.tools.gene_network)

class indra.tools.gene_network.**GeneNetwork**(*gene_list*, *basename=None*)

Build a set of INDRA statements for a given gene list from databases.

Parameters

- **gene_list** (*string*) – List of gene names.
- **basename** (*string or None (default)*) – Filename prefix to be used for caching of intermediates (Biopax OWL file, pickled statement lists, etc.). If None, no results are cached and no cached files are used.

gene_list

string – List of gene names

basename
string or None – Filename prefix for cached intermediates, or None if no cached used.

results
dict – Dict containing results of preassembly (see return type for `run_preassembly()`.

get_bel_stmts (*filter=False*)
Get relevant statements from the BEL large corpus.

Performs a series of neighborhood queries and then takes the union of all the statements. Because the query process can take a long time for large gene lists, the resulting list of statements are cached in a pickle file with the filename *<basename>_bel_stmts.pkl*. If the pickle file is present, it is used by default; if not present, the queries are performed and the results are cached.

Parameters **filter** (`bool`) – If True, includes only those statements that exclusively mention genes in `gene_list`. Default is False. Note that the full (unfiltered) set of statements are cached.

Returns List of INDRA statements extracted from the BEL large corpus.

Return type list of `indra.statements.Statement`

get_biopax_stmts (*filter=False*, *query=u'pathsbetween'*)
Get relevant statements from Pathway Commons.

Performs a "paths between" query for the genes in `gene_list` and uses the results to build statements. This function caches two files: the list of statements built from the query, which is cached in *<basename>_biopax_stmts.pkl*, and the OWL file returned by the Pathway Commons Web API, which is cached in *<basename>_pc_pathsbetween.owl*. If these cached files are found, then the results are returned based on the cached file and Pathway Commons is not queried again.

Parameters

- **filter** (`bool`) – If True, includes only those statements that exclusively mention genes in `gene_list`. Default is False.
- **query** (`str`) – Defined what type of query is executed. The two options are 'pathsbetween' which finds paths between the given list of genes and only works if more than 1 gene is given, and 'neighborhood' which searches the immediate neighborhood of each given gene.

Returns List of INDRA statements extracted from Pathway Commons.

Return type list of `indra.statements.Statement`

get_statements (*filter=False*)
Return the combined list of statements from BEL and Pathway Commons.

Internally calls `get_biopax_stmts()` and `get_bel_stmts()`.

Parameters **filter** (`bool`) – If True, includes only those statements that exclusively mention genes in `gene_list`. Default is False.

Returns List of INDRA statements extracted the BEL large corpus and Pathway Commons.

Return type list of `indra.statements.Statement`

run_preassembly (*stmts*, *print_summary=True*)
Run complete preassembly procedure on the given statements.

Results are returned as a dict and stored in the attribute `results`. They are also saved in the pickle file *<basename>_results.pkl*.

Parameters

- **stmts** (list of *indra.statements.Statement*) – Statements to preassemble.
- **print_summary** (*bool*) – If True (default), prints a summary of the preassembly process to the console.

Returns

A dict containing the following entries:

- *raw*: the starting set of statements before preassembly.
- *duplicates1*: statements after initial de-duplication.
- *valid*: statements found to have valid modification sites.
- *mapped*: mapped statements (list of *indra.preassembler.sitemapper.MappedStatement*).
- *mapped_stmts*: combined list of valid statements and statements after mapping.
- *duplicates2*: statements resulting from de-duplication of the statements in *mapped_stmts*.
- *related2*: top-level statements after combining the statements in *duplicates2*.

Return type dict

4.10.3 Build an executable model from a fragment of a large network (indra.tools.executable_subnetwork)

indra.tools.executable_subnetwork.**get_subnetwork**(*statements*, *nodes*, *relevance_network=None*, *relevance_node_lim=10*)

Return a PySB model based on a subset of given INDRA Statements.

Statements are first filtered for nodes in the given list and other nodes are optionally added based on relevance in a given network. The filtered statements are then assembled into an executable model using INDRA's PySB Assembler.

Parameters

- **statements** (*list[indra.statements.Statement]*) – A list of INDRA Statements to extract a subnetwork from.
- **nodes** (*list[str]*) – The names of the nodes to extract the subnetwork for.
- **relevance_network** (*Optional[str]*) – The UUID of the NDEx network in which nodes relevant to the given nodes are found.
- **relevance_node_lim** (*Optional[int]*) – The maximal number of additional nodes to add to the subnetwork based on relevance.

Returns **model** – A PySB model object assembled using INDRA's PySB Assembler from the INDRA Statements corresponding to the subnetwork.

Return type pysb.Model

4.10.4 Build a model incrementally over time (indra.tools.incremental_model)

class indra.tools.incremental_model.**IncrementalModel**(*model_fname=None*)

Assemble a model incrementally by iteratively adding new Statements.

Parameters **model_fname** (*Optional[str]*) – The name of the pickle file in which a set of INDRA Statements are stored in a dict keyed by PubMed IDs. This is the state of an IncrementalModel that is loaded upon instantiation.

stmts
dict[str, list[indra.statements.Statement]] – A dictionary of INDRA Statements keyed by PMIDs that stores the current state of the IncrementalModel.

assembled_stmts
list[indra.statements.Statement] – A list of INDRA Statements after assembly.

add_statements (*pmid*, *stmts*)
Add INDRA Statements to the incremental model indexed by PMID.

Parameters

- **pmid** (*str*) – The PMID of the paper from which statements were extracted.
- **stmts** (*list[indra.statements.Statement]*) – A list of INDRA Statements to be added to the model.

get_model_agents ()
Return a list of all Agents from all Statements.

Returns **agents** – A list of Agents that are in the model.

Return type list[indra.statements.Agent]

get_statements ()
Return a list of all Statements in a single list.

Returns **stmts** – A list of all the INDRA Statements in the model.

Return type list[indra.statements.Statement]

get_statements_noprior ()
Return a list of all non-prior Statements in a single list.

Returns **stmts** – A list of all the INDRA Statements in the model (excluding the prior).

Return type list[indra.statements.Statement]

get_statements_prior ()
Return a list of all prior Statements in a single list.

Returns **stmts** – A list of all the INDRA Statements in the prior.

Return type list[indra.statements.Statement]

load_prior (*prior_fname*)
Load a set of prior statements from a pickle file.

The prior statements have a special key in the stmts dictionary called “prior”.

Parameters **prior_fname** (*str*) – The name of the pickle file containing the prior Statements.

preassemble (*filters=None*)
Preassemble the Statements collected in the model.

Use INDRA’s GroundingMapper, Preassembler and BeliefEngine on the IncrementalModel and save the unique statements and the top level statements in class attributes.

Currently the following filter options are implemented: - grounding: require that all Agents in statements are grounded - human_only: require that all proteins are human proteins - prior_one: require that at least one Agent is in the prior model - prior_all: require that all Agents are in the prior model

Parameters **filters** (*Optional[list[str]]*) – A list of filter options to apply when choosing the statements. See description above for more details. Default: None

save (*model_fname=u'model.pkl'*)

Save the state of the IncrementalModel in a pickle file.

Parameters **model_fname** (*Optional[str]*) – The name of the pickle file to save the state of the IncrementalModel in. Default: model.pkl

CHAPTER

FIVE

TUTORIALS

5.1 Using natural language to build models

In this tutorial we build a simple model using natural language, and export it into different formats.

5.1.1 Read INDRA Statements from a natural language string

First we import INDRA's API to the TRIPS reading system. We then define a block of text which serves as the description of the mechanism to be modeled in the *model_text* variable. Finally, *indra.sources.trips.process_text* is called which sends a request to the TRIPS web service, gets a response and processes the extraction knowledge base to obtain a list of INDRA Statements

```
In [1]: from indra.sources import trips

In [2]: model_text = 'MAP2K1 phosphorylates MAPK1 and DUSP6 dephosphorylates MAPK1.'

In [3]: tp = trips.process_text(model_text)
```

At this point *tp.statements* should contain 2 INDRA Statements: a Phosphorylation Statement and a Dephosphorylation Statement. Note that the evidence sentence for each Statement is propagated:

```
In [4]: for st in tp.statements:
   ...:     print('%s with evidence "%s"' % (st, st.evidence[0].text))
   ...:
Phosphorylation(MAP2K1(), MAPK1()) with evidence "MAP2K1 phosphorylates MAPK1 and␣
↪DUSP6 dephosphorylates MAPK1."
Dephosphorylation(DUSP6(), MAPK1()) with evidence "MAP2K1 phosphorylates MAPK1 and␣
↪DUSP6 dephosphorylates MAPK1."
```

5.1.2 Assemble the INDRA Statements into a rule-based executable model

We next use INDRA's PySB Assembler to automatically assemble a rule-based model representing the biochemical mechanisms described in *model_text*. First a PysbAssembler object is instantiated, then the list of INDRA Statements is added to the assembler. Finally, the assembler's *make_model* method is called which assembles the model and returns it, while also storing it in *pa.model*. Notice that we are using *policies='two_step'* as an argument of *make_model*. This directs the assemble to use rules in which enzymatic catalysis is modeled as a two-step process in which enzyme and substrate first reversibly bind and the enzyme-substrate complex produces and releases a product irreversibly.

```
In [5]: from indra.assemblers.pysb_assembler import PysbAssembler
```

```
In [6]: pa = PysbAssembler()

In [7]: pa.add_statements(tp.statements)

In [8]: pa.make_model(policies='two_step')
Out[8]: <Model 'None' (monomers: 3, rules: 6, parameters: 9, expressions: 0,␣
↪compartments: 0) at 0x7f1e30e21610>
```

At this point *pa.model* contains a PySB model object with 3 monomers,

```
In [9]: for monomer in pa.model.monomers:
   ...:     print(monomer)
   ...:
Monomer(u'DUSP6', [u'mapk1'])
Monomer(u'MAP2K1', [u'mapk1'])
Monomer(u'MAPK1', [u'phospho', u'map2k1', u'dusp6'], {u'phospho': [u'u', u'p']})
```

6 rules,

```
In [10]: for rule in pa.model.rules:
   ....:     print(rule)
   ....:
Rule(u'MAP2K1_phosphorylation_bind_MAPK1_phospho', MAP2K1(mapk1=None) +␣
↪MAPK1(phospho=u'u', map2k1=None) >> MAP2K1(mapk1=1) % MAPK1(phospho=u'u',␣
↪map2k1=1), kf_mm_bind_1)
Rule(u'MAP2K1_phosphorylation_MAPK1_phospho', MAP2K1(mapk1=1) % MAPK1(phospho=u'u',␣
↪map2k1=1) >> MAP2K1(mapk1=None) + MAPK1(phospho=u'p', map2k1=None), kc_mm_
↪phosphorylation_1)
Rule(u'MAP2K1_dissoc_MAPK1', MAP2K1(mapk1=1) % MAPK1(map2k1=1) >> MAP2K1(mapk1=None)␣
↪+ MAPK1(map2k1=None), kr_mm_bind_1)
Rule(u'DUSP6_dephosphorylation_bind_MAPK1_phospho', DUSP6(mapk1=None) +␣
↪MAPK1(phospho=u'p', dusp6=None) >> DUSP6(mapk1=1) % MAPK1(phospho=u'p', dusp6=1),␣
↪kf_dm_bind_1)
Rule(u'DUSP6_dephosphorylation_MAPK1_phospho', DUSP6(mapk1=1) % MAPK1(phospho=u'p',␣
↪dusp6=1) >> DUSP6(mapk1=None) + MAPK1(phospho=u'u', dusp6=None), kc_dm_
↪dephosphorylation_1)
Rule(u'DUSP6_dissoc_MAPK1', DUSP6(mapk1=1) % MAPK1(dusp6=1) >> DUSP6(mapk1=None) +␣
↪MAPK1(dusp6=None), kr_dm_bind_1)
```

and 9 parameters (6 kinetic rate constants and 3 total protein amounts) that are set to nominal but plausible values,

```
In [11]: for parameter in pa.model.parameters:
   ....:     print(parameter)
   ....:
Parameter(u'kf_mm_bind_1', 1e-06)
Parameter(u'kr_mm_bind_1', 0.1)
Parameter(u'kc_mm_phosphorylation_1', 100.0)
Parameter(u'kf_dm_bind_1', 1e-06)
Parameter(u'kr_dm_bind_1', 0.1)
Parameter(u'kc_dm_dephosphorylation_1', 100.0)
Parameter(u'DUSP6_0', 10000.0)
Parameter(u'MAP2K1_0', 10000.0)
Parameter(u'MAPK1_0', 10000.0)
```

The model also contains extensive annotations that tie the monomers to database identifiers and also annotate the semantics of each component of each rule.

```
In [12]: for annotation in pa.model.annotations:
   ....:     print(annotation)
   ....:
Annotation(DUSP6, u'http://identifiers.org/ncit/C106026', u'is')
Annotation(DUSP6, u'http://identifiers.org/uniprot/Q16828', u'is')
Annotation(DUSP6, u'http://identifiers.org/hgnc/HGNC:3072', u'is')
Annotation(MAP2K1, u'http://identifiers.org/ncit/C17808', u'is')
Annotation(MAP2K1, u'http://identifiers.org/uniprot/Q02750', u'is')
Annotation(MAP2K1, u'http://identifiers.org/hgnc/HGNC:6840', u'is')
Annotation(MAPK1, u'http://identifiers.org/ncit/C17589', u'is')
Annotation(MAPK1, u'http://identifiers.org/uniprot/P28482', u'is')
Annotation(MAPK1, u'http://identifiers.org/hgnc/HGNC:6871', u'is')
Annotation(MAP2K1_phosphorylation_bind_MAPK1_phospho, u'ba5e8261-d19e-404a-8b79-
↪5685b539e97f', u'from_indra_statement')
Annotation(MAP2K1_phosphorylation_MAPK1_phospho, u'MAP2K1', u'rule_has_subject')
Annotation(MAP2K1_phosphorylation_MAPK1_phospho, u'MAPK1', u'rule_has_object')
Annotation(MAP2K1_phosphorylation_MAPK1_phospho, u'ba5e8261-d19e-404a-8b79-
↪5685b539e97f', u'from_indra_statement')
Annotation(MAP2K1_dissoc_MAPK1, u'ba5e8261-d19e-404a-8b79-5685b539e97f', u'from_indra_
↪statement')
Annotation(DUSP6_dephosphorylation_bind_MAPK1_phospho, u'ac5dccaa-f1ba-4516-a9e4-
↪ec86f72aa067', u'from_indra_statement')
Annotation(DUSP6_dephosphorylation_MAPK1_phospho, u'DUSP6', u'rule_has_subject')
Annotation(DUSP6_dephosphorylation_MAPK1_phospho, u'MAPK1', u'rule_has_object')
Annotation(DUSP6_dephosphorylation_MAPK1_phospho, u'ac5dccaa-f1ba-4516-a9e4-
↪ec86f72aa067', u'from_indra_statement')
Annotation(DUSP6_dissoc_MAPK1, u'ac5dccaa-f1ba-4516-a9e4-ec86f72aa067', u'from_indra_
↪statement')
```

5.1.3 Exporting the model into other common formats

From the assembled PySB format it is possible to export the model into other common formats such as SBML, BNGL and Kappa. One can also generate a Matlab or Mathematica script with ODEs corresponding to the model.

```
pa.export_model('sbml')
pa.export_model('bngl')
```

One can also pass a file name argument to the *export_model* function to save the exported model directly into a file:

```
pa.export_model('sbml', 'example_model.sbml')
```

5.2 Large-Scale Machine Reading with Starcluster

The following doc describes the steps involved in reading a large numbers of papers in parallel on Amazon EC2 using REACH, caching the JSON output on Amazon S3, then processing the REACH output into INDRA Statements. Prerequisites for doing the following are:

- A cluster of Amazon EC2 nodes configured using Starcluster, with INDRA installed and in the PYTHONPATH
- An Amazon S3 bucket containing full text contents for papers, keyed by Pubmed ID (creation of this S3 repository will be described in another tutorial).

This tutorial goes through the individual steps involved before describing how all of them can be run through the use of a single submission script, submit_reading_pipeline.py.

Note also that the prerequisite installation steps can be streamlined by putting them in a setup script that can be re-run upon instantiating a new Amazon cluster or by using them to configure a custom Amazon EC2 AMI.

5.2.1 Install REACH

Install SBT. On an EC2 Linux machine, run the following lines (drawn from http://www.scala-sbt.org/0.13/docs/Installing-sbt-on-Linux.html):

```
echo "deb https://dl.bintray.com/sbt/debian /" | sudo tee -a /etc/apt/sources.list.d/
↪sbt.list
sudo apt-key adv --keyserver hkp://keyserver.ubuntu.com:80 --recv 642AC823
sudo apt-get update
sudo apt-get install sbt
```

Clone REACH from https://github.com/clulab/reach.

Add the following line to reach/build.sbt:

```
mainClass in assembly := Some("org.clulab.reach.ReachCLI")
```

This assigns ReachCLI as the main class.

Compile and assemble REACH. Note that the path to the .ivy2 directory must be given. Use the assembly task to assemble a fat JAR containing all of the dependencies with the correct main class. Run the following from the directory containing the REACH build.sbt file (e.g., /pmc/reach).:

```
sbt -Dsbt.ivy.home=/pmc/reach/.ivy2 compile
sbt -Dsbt.ivy.home=/pmc/reach/.ivy2 assembly
```

5.2.2 Install Amazon S3 support

Install boto3:

```
pip install boto3
```

Note: If using EC2, make sure to install boto3, jsonpickle, and Amazon credentials on all nodes, not just the master node.

Add Amazon credentials to access the S3 bucket. First create the .aws directory on the EC2 instance:

```
mkdir /home/sgeadmin/.aws
```

Then set up Amazon credentials, for example by copying from your local machine using StarCluster:

```
starcluster put mycluster ~/.aws/credentials /home/sgeadmin/.aws
```

5.2.3 Install other dependencies

```
pip install jsonpickle # Necessary to process JSON from S3
pip install --upgrade jnius-indra # Necessary for REACH
export JAVA_HOME=/usr/lib/jvm/java-1.7.0-openjdk-amd64
```

5.2.4 Assemble a Corpus of PMIDs

The first step in large-scale reading is to put together a file containing relevant Pubmed IDs. The simplest way to do this is to use the Pubmed search API to find papers associated with particular gene names, biological processes, or other search terms.

For example, to assemble a list of papers for SOS2 curated in Entrez Gene that are available in the Pubmed Central Open Access subset:

```
In [1]: from indra.literature import *

# Pick an example gene
In [2]: gene = 'SOS2'

# Get a list of PMIDs for the gene
In [3]: pmids = pubmed_client.get_ids_for_gene(gene)

# Get the PMIDs that have XML in PMC
In [4]: pmids_oa_xml = pmc_client.filter_pmids(pmids, 'oa_xml')

# Write the results to a file
In [5]: with open('%s_pmids.txt' % gene, 'w') as f:
   ...:     for pmid in pmids_oa_xml:
   ...:         f.write('%s\n' % pmid)
   ...:
```

This creates a file, SOS2_pmids.txt, containing the PMIDs that we will read with REACH.

5.2.5 Process the papers with REACH

The next step is to read the content of the papers with REACH in a parallelizable, high-throughput way. To do this, run the script indra/tools/reading/run_reach_on_pmids.py. If necessary update the lines at the top of the script with the REACH settings, e.g.:

```
cleanup = False
verbose = True
path_to_reach = '/pmc/reach/target/scala-2.11/reach-assembly-1.3.2-SNAPSHOT.jar'
reach_version = '1.3.2'
source_text = 'pmc_oa_xml'
force_read = False
```

The reach_version is important because it is used to determine whether the paper has already been read with this version of REACH (in which case it will be skipped), or if the REACH output needs to be updated. Alternatively, if you want to read all the papers regardless of whether they've been read before with the given version of REACH, set the force_read variable to True.

Next, create a top-level temporary directory to use during reading. This will be used to store the input files and the JSON output:

```
mkdir my_temp_dir
```

Run run_reach_on_pmids.py, passing arguments for the PMID list file, the temp directory, the number of cores to use on the machine, the PMID start index (in the PMID list file) and the end index. The start and end indices are used to subdivide the job into parallelizable chunks. If the end index is greater than the total number of PMIDs, it will process up to the last one in the list. For example:

```
python run_reach_on_pmids.py SOS2_pmids.txt my_temp_dir 8 0 10
```

This uses 8 cores to process the first ten papers listed in the file SOS2_pmids.txt. REACH will run, output the JSON files in the temporary directory, e.g. in my_temp_dir/read_0_to_10_MSP6YI/output, assemble the JSON files together, and upload the results to S3. If you attempt to process the files again with the same version of REACH, the script will detect that the JSON output from that version is already on S3 and skip those papers.

This can be submitted to run offline using the job scheduler on EC2 with, e.g.:

```
qsub -b y -cwd -V -pe orte 8 python run_reach_on_pmids.py SOS2_pmids.txt my_temp_dir␣
↪8 0 10
```

Note: The number of cores requested in the qsub call ('-pe orte 8') should match the number of cores passed to the run_reach_on_pmids.py script, which determines the number of threads that REACH will attempt to use (the third-to-last argument above). This should also match the total number of nodes on the Amazon EC2 node (e.g., 8 cores for c3.2xlarge). This way the job scheduler will schedule the job to run on all the cores of a single EC2 node, and REACH will use them all.

5.2.6 Extract INDRA Statements from the REACH output on S3

The script indra/tools/reading/process_reach_from_s3.py is used to extract INDRA Statements from the REACH output uploaded to S3 in the previous step. This process can also be parallelized by submitting chunks of papers to be processed by different cores. The INDRA statements for each chunk of papers are pickled and can be assembled into a single pickle file in a subsequent step.

Following the example above, run the following to process the REACH output for the SOS2 papers into INDRA statements. We'll do this in two chunks to show how the process can be parallelized and the statements assembled from multiple files:

```
python process_reach_from_s3.py SOS2_pmids.txt 0 5
python process_reach_from_s3.py SOS2_pmids.txt 5 10
```

The two runs create two different files for the results from the seven papers, reach_stmts_0_5.pkl (with statements from the first five papers) and reach_stmts_5_7.pkl (with statements from the last two). Note that the results are pickled as a dict (rather than a list), with PMIDs as keys and lists of Statements as values.

Of course, what we really want is a single file containing all of the statements for the entire corpus. To get this, run:

```
python assemble_reach_stmts.py reach_stmts_*.pkl
```

The results will be stored in reach_stmts.pkl.

5.2.7 Running the whole pipeline with one script

If you want to run the whole pipeline in one go, you can run the script submit_reading_pipeline.py (in indra/tools/reading) on a cluster of Amazon EC2 nodes. The script divides up the jobs evenly among the nodes and cores. Usage:

```
python submit_reading_pipeline.py pmid_list tmp_dir num_nodes num_cores_per_node
```

For example if you have a cluster with 8 c3.8xlarge nodes with 32 VCPUs each, you would call it with:

```
python submit_reading_pipeline.py SOS2_pmids.txt my_tmp_dir 8 32
```

The script submits the jobs to the scheduler with appropriate dependencies such that the REACH reading step completes first, then the INDRA processing step, and then the final assembly into a single pickle file.

5.3 Large-Scale Machine Reading with Amazon Batch

The following doc describes the steps involved in reading a large numbers of papers in parallel on Amazon EC2 using REACH, caching the JSON output on Amazon S3, processing the REACH output into INDRA Statements, and then caching the statements also on S3. Prerequisites for doing the following are:

- An Amazon S3 bucket containing full text contents for papers, keyed by Pubmed ID (creation of this S3 repository will be described in another tutorial).
- Amazon AWS credentials for using AWS Batch.
- A corpus of PMIDs (see *Large-Scale Machine Reading with Starcluster* for information on how to assemble this)
- Optional: Elsevier text and data mining API key and institution key for subscriber access to Elsevier full text content.

5.3.1 How it Works

- The reading pipeline makes use of a Docker image that contains INDRA and all necessary dependencies, including REACH, Kappa, PySB, etc. The Docker file for this image is available at: https://github.com/johnbachman/indra_docker.
- The INDRA Docker image is built by AWS Codebuild and pushed to Amazon's EC2 Container Service (ECS), where it is available via the Repository URI:

  ```
  292075781285.dkr.ecr.us-east-1.amazonaws.com/indra
  ```

- An AWS Batch *Compute Environment* named "run_reach" is configured to use this Docker image for handling AWS jobs. This compute environment is configured to use only Spot instances with a maximum spot price of 40% of the on-demand price, and 16 vCPUs.
- An AWS *Job Queue*, "run_reach_queue", is configured to use instances of the "run_reach" Compute Environment.
- An AWS *Job Definition*, "run_reach_jobdef", is configured to run in the "run_reach_queue", and to use 16 vCPUs and 30GiB of RAM.
- Reading jobs are submitted by running the script:

  ```
  python -m indra.tools.reading.submit_reading_pipeline_aws read [args]
  ```

  which, given a list of PMIDs:

  – Copies the PMID list to the key `reading_results/[job_name]/pmids` on Amazon S3

  – Breaks the list up into chunks (e.g., of 3000 PMIDs) and submits an AWS Batch job for each (using the "run_reach_jobdef" definition as a template).

- The ECS instance created by the AWS Batch job runs the script `indra.tools.reading.run_reach_on_pmids_aws`, which:

  - Checks for cached content on Amazon S3
  - If the PMID has not been read by the current version of REACH, checks for content
  - If the content is not available, downloads the content using the INDRA literature client, and caches on S3
  - The content to be read is downloaded to the `/tmp` directory of the instance
  - REACH is run using the command-line interface (RunReachCLI), and configured to read the papers in the `/tmp` directory using all of the vCPUs on the instance
  - When done, the result REACH JSON in the output folder is uploaded to S3
  - The JSON for both the previously and newly read papers is processed in parallel to INDRA Statements
  - The resulting subset of statements for the given range of papers is cached on S3 at `reading_results/[job_name]/stmts/[start_ix]_[end_ix].pkl`. This set of statements takes the form of a pickled (protocol 3) Python dict with PMIDs as keys and lists of INDRA Statements as values.
  - In addition, information about the sources of content available for each PMID is cached for each PMID subset at `reading_results/[job_name]/content_types/[start_ix]_[end_ix].pkl`.
- When the reading jobs for each of the subsets of PMIDs have been completed and cached on S3, the final combined set of statements (and combined information on content sources) can be assembled using:

```
python -m indra.tools.reading.submit_reading_pipeline_aws combine [job_name]
```

  - This script submits an AWS batch job for a machine with 1 vCPU but a large amount of memory (60GiB)
  - The job runs the script `indra.tools.reading.assemble_reach_stmts_aws`, which unpickles the results from all of the PMID subsets, combines them, and stores them on S3
  - The resulting files are obtainable from S3 at `reading_results/[job_name]/stmts.pkl` and `reading_results/[job_name]/content_types.pkl`.
- To run the entire pipeline, where the assembly of the combined set of statements is automatically performed after the reading step is completed, run:

```
python -m indra.tools.reading.submit_reading_pipeline_aws full [args]
```

5.4 Assembling everything known about a particular gene

Assume you are interested in collecting all mechanisms that a particular gene is involved in. Using INDRA, it is possible to collect everything curated about the gene in pathway databases and then read all the accessible literature discussing the gene of interest. This knowledge is aggregated as a set of INDRA Statements which can then be assembled into several different model and network formats and possibly shared online.

For the sake of example, assume that the gene of interest is TMEM173.

It is important to use the standard HGNC gene symbol of the gene throughout the example (this information is available on http://www.genenames.org/ or http://www.uniprot.org/) - abritrary synonyms will not work!

5.4.1 Collect mechanisms from PathwayCommons and the BEL Large Corpus

We first collect Statements from the PathwayCommons database via INDRA's BioPAX API and then collect Statements from the BEL Large Corpus via INDRA's BEL API.

```
from indra.tools.gene_network import GeneNetwork

gn = GeneNetwork(['TMEM173'])
biopax_stmts = gn.get_biopax_stmts()
bel_stmts = gn.get_bel_stmts()
```

at this point *biopax_stmts* and *bel_stmts* are two lists of INDRA Statements.

5.4.2 Collect a list of publications that discuss the gene of interest

We next use INDRA's literature client to find PubMed IDs (PMIDs) that discuss the gene of interest. To find articles that are annotated with the given gene, INDRA first looks up the Entrez ID corresponding to the gene name and then finds associated publications.

```
from indra import literature

pmids = literature.pubmed_client.get_ids_for_gene('TMEM173')
```

The variable *pmid* now contains a list of PMIDs associated with the gene.

5.4.3 Get the full text or abstract corresponding to the publications

Next we use INDRA's literature client to fetch the full text (if available) or the abstract corresponding to the PMIDs we have just collected.

```
from indra import literature

paper_contents = {}
for pmid in pmids:
    content, content_type = literature.get_full_text(pmid, 'pmid')
    paper_contents[pmid] = (content, content_type)
```

We now have a dictionary called *paper_contents* which stores the content and the content type of each PMID we looked up.

5.4.4 Read the content of the publications

We next run the REACH reading system on the publications. Depending on the content type, different calls need to be made via INDRA's REACH API.

```
from indra import literature
from indra.sources import reach

read_offline = True

literature_stmts = []
for pmid, (content, content_type) in paper_contents.items():
    rp = None
    print('Reading %s' % pmid)
    if content_type == 'abstract':
        rp = reach.process_text(content, citation=pmid, offline=read_offline)
    elif content_type == 'pmc_oa_xml':
        rp = reach.process_nxml_str(content, offline=read_offline)
```

```
    elif content_type == 'elsevier_xml':
        txt = literature.elsevier_client.extract_text(content)
        if txt:
            rp = reach.process_text(txt, citation=pmid, offline=read_offline)
    if rp is not None:
        literature_stmts += rp.statements
```

The list *literature_stmts* now contains the results of all the statements that were read.

5.4.5 Combine all statements and run pre-assembly

```
from indra.tools import assemble_corpus

stmts = biopax_stmts + bel_stmts + literature_stmts

stmts = assemble_corpus.map_grounding(stmts)
stmts = assemble_corpus.map_sequence(stmts)
stmts = assemble_corpus.run_preassembly(stmts)
```

At this point *stmts* contains a list of Statements collected with grounding, sequences having been mapped, duplicates combined and less specific variants of statements hidden. It is possible to run other filters on the results such as to keep only human genes, remove Statements with ungrounded genes, or to keep only certain types of interactions.

5.4.6 Assemble the statements into a network model

```
from indra.assemblers import CxAssembler

cxa = CxAssembler(stmts)
cxa.make_model()
```

we can now upload this network to the Network Data Exchange (NDEx).

```
ndex_cred = {'user': 'myusername', 'password': 'xxx'}
network_id = cxa.upload_model(ndex_cred)
print(network_id)
```

CHAPTER

SIX

REST API

Many functionalities of INDRA can be used via a REST API. This enables making use of INDRA's knowledge sources and assembly capabilities in a RESTful, platform independent fashion. The REST service is meant to be used locally (on a single machine or local network) and is currently not offered as a public web service by the creators of INDRA.

6.1 Installation

The REST service requires the *bottle* package to be installed in addition to all the other requirements of INDRA.

6.2 Launching the REST service

The REST service can be launched by running *api.py* in the *rest_api* folder within *indra*.

6.3 Documentation

The specific end-points and input/output parameters offered by the REST API are documented in *rest_api/docs/index.html*, which is accessible locally within the *indra* folder.

CHAPTER

SEVEN

INDICES AND TABLES

- genindex
- modindex
- search

i

indra.assemblers.cx_assembler, 67
indra.assemblers.english_assembler, 68
indra.assemblers.graph_assembler, 68
indra.assemblers.index_card_assembler, 71
indra.assemblers.pysb_assembler, 64
indra.assemblers.sbgn_assembler, 71
indra.assemblers.sif_assembler, 70
indra.belief, 60
indra.databases.biogrid_client, 45
indra.databases.cbio_client, 46
indra.databases.chebi_client, 44
indra.databases.context_client, 45
indra.databases.hgnc_client, 40
indra.databases.ndex_client, 46
indra.databases.relevance_client, 45
indra.databases.uniprot_client, 41
indra.explanation.model_checker, 72
indra.literature, 49
indra.literature.crossref_client, 51
indra.literature.elsevier_client, 51
indra.literature.pmc_client, 50
indra.literature.pubmed_client, 50
indra.mechlinker, 61
indra.preassembler, 51
indra.preassembler.grounding_mapper, 56
indra.preassembler.hierarchy_manager, 58
indra.preassembler.sitemapper, 56
indra.sources.bel.bel_api, 25
indra.sources.bel.processor, 26
indra.sources.biopax.biopax_api, 29
indra.sources.biopax.pathway_commons_client, 33
indra.sources.biopax.processor, 31
indra.sources.ndex_cx.ndex_cx_api, 39
indra.sources.ndex_cx.processor, 39
indra.sources.reach.processor, 35
indra.sources.reach.reach_api, 33
indra.sources.reach.reach_reader, 36
indra.sources.trips.processor, 37
indra.sources.trips.trips_api, 36
indra.sources.trips.trips_client, 38
indra.statements, 13
indra.tools.assemble_corpus, 74
indra.tools.executable_subnetwork, 83
indra.tools.gene_network, 81
indra.tools.incremental_model, 83

A

Acetylation (class in indra.statements), 15
Activation (class in indra.statements), 15
ActiveForm (class in indra.statements), 15
ActivityCondition (class in indra.statements), 16
add_default_initial_conditions() (indra.assemblers.pysb_assembler.PysbAssembler method), 64
add_rule_to_model() (in module indra.assemblers.pysb_assembler), 65
add_statements() (indra.assemblers.cx_assembler.CxAssembler method), 67
add_statements() (indra.assemblers.english_assembler.EnglishAssembler method), 68
add_statements() (indra.assemblers.graph_assembler.GraphAssembler method), 69
add_statements() (indra.assemblers.index_card_assembler.IndexCardAssembler method), 71
add_statements() (indra.assemblers.pysb_assembler.PysbAssembler method), 64
add_statements() (indra.assemblers.sbgn_assembler.SBGNAssembler method), 71
add_statements() (indra.explanation.model_checker.ModelChecker method), 72
add_statements() (indra.mechlinker.MechLinker method), 62
add_statements() (indra.preassembler.Preassembler method), 52
add_statements() (indra.tools.incremental_model.IncrementalModel method), 84
Agent (class in indra.statements), 16
agent_set (indra.assemblers.pysb_assembler.PysbAssembler attribute), 64
AgentState (class in indra.mechlinker), 61
all_direct_stmts (indra.sources.bel.processor.BelProcessor attribute), 26
all_events (indra.sources.reach.processor.ReachProcessor attribute), 36
all_indirect_stmts (indra.sources.bel.processor.BelProcessor attribute), 26
api_ruler (indra.sources.reach.reach_reader.ReachReader attribute), 36
apply_to() (indra.mechlinker.AgentState method), 61
assembled_stmts (indra.tools.incremental_model.IncrementalModel attribute), 84
Autophosphorylation (class in indra.statements), 16

B

BaseAgent (class in indra.mechlinker), 61
BaseAgentSet (class in indra.mechlinker), 61
basename (indra.tools.gene_network.GeneNetwork attribute), 81
BeliefEngine (class in indra.belief), 60
BelProcessor (class in indra.sources.bel.processor), 26
BiopaxProcessor (class in indra.sources.biopax.processor), 31
bound_conditions (indra.mechlinker.AgentState attribute), 61
BoundCondition (class in indra.statements), 17
build_transitive_closures() (indra.preassembler.hierarchy_manager.HierarchyManager method), 59

C

check_model() (indra.explanation.model_checker.ModelChecker method), 72
check_statement() (indra.explanation.model_checker.ModelChecker method), 73
citation (indra.sources.reach.processor.ReachProcessor attribute), 36
combine_duplicate_stmts() (indra.preassembler.Preassembler static method), 52
combine_duplicates() (indra.preassembler.Preassembler method), 52
combine_related() (indra.preassembler.Preassembler method), 52
Complex (class in indra.statements), 17
complex_monomers_default() (in module indra.assemblers.pysb_assembler), 65
complex_monomers_one_step() (in module indra.assemblers.pysb_assembler), 66
Conversion (class in indra.statements), 17

converted_direct_stmts (indra.sources.bel.processor.BelProcessor attribute), 26
converted_indirect_stmts (indra.sources.bel.processor.BelProcessor attribute), 26
cx (indra.assemblers.cx_assembler.CxAssembler attribute), 67
CxAssembler (class in indra.assemblers.cx_assembler), 67

D

Deacetylation (class in indra.statements), 18
DecreaseAmount (class in indra.statements), 18
Defarnesylation (class in indra.statements), 18
default_mapper (in module indra.preassembler.sitemapper), 58
degenerate_stmts (indra.sources.bel.processor.BelProcessor attribute), 26
Degeranylgeranylation (class in indra.statements), 18
Deglycosylation (class in indra.statements), 18
Dehydroxylation (class in indra.statements), 18
Demethylation (class in indra.statements), 18
Demyristoylation (class in indra.statements), 18
Depalmitoylation (class in indra.statements), 18
Dephosphorylation (class in indra.statements), 19
Deribosylation (class in indra.statements), 19
Desumoylation (class in indra.statements), 19
Deubiquitination (class in indra.statements), 19
doc_id (indra.sources.trips.processor.TripsProcessor attribute), 37
doi_query() (in module indra.literature.crossref_client), 51
download_article() (in module indra.literature.elsevier_client), 51
dump_statements() (in module indra.tools.assemble_corpus), 74
dump_stmt_strings() (in module indra.tools.assemble_corpus), 74

E

edge_properties (indra.assemblers.graph_assembler.GraphAssembler attribute), 69
EnglishAssembler (class in indra.assemblers.english_assembler), 68
Evidence (class in indra.statements), 19
existing_edges (indra.assemblers.graph_assembler.GraphAssembler attribute), 69
existing_nodes (indra.assemblers.graph_assembler.GraphAssembler attribute), 69
expand_families() (in module indra.tools.assemble_corpus), 74
expand_pagination() (in module indra.literature.pubmed_client), 50
export_model() (indra.assemblers.pysb_assembler.PysbAssembler method), 64
extracted_events (indra.sources.trips.processor.TripsProcessor attribute), 37

F

Farnesylation (class in indra.statements), 19
filter_belief() (in module indra.tools.assemble_corpus), 74
filter_by_type() (in module indra.tools.assemble_corpus), 74
filter_direct() (in module indra.tools.assemble_corpus), 75
filter_enzyme_kinase() (in module indra.tools.assemble_corpus), 75
filter_evidence_source() (in module indra.tools.assemble_corpus), 75
filter_gene_list() (in module indra.tools.assemble_corpus), 75
filter_genes_only() (in module indra.tools.assemble_corpus), 76
filter_grounded_only() (in module indra.tools.assemble_corpus), 76
filter_human_only() (in module indra.tools.assemble_corpus), 76
filter_inconsequential_acts() (in module indra.tools.assemble_corpus), 77
filter_inconsequential_mods() (in module indra.tools.assemble_corpus), 77
filter_mod_nokinase() (in module indra.tools.assemble_corpus), 77
filter_mutation_status() (in module indra.tools.assemble_corpus), 77
filter_no_hypothesis() (in module indra.tools.assemble_corpus), 78
filter_pmids() (in module indra.literature.pmc_client), 50
filter_top_level() (in module indra.tools.assemble_corpus), 78
filter_transcription_factor() (in module indra.tools.assemble_corpus), 78
filter_uuid_list() (in module indra.tools.assemble_corpus), 79
flatten_evidence() (in module indra.preassembler), 54
flatten_stmts() (in module indra.preassembler), 55

G

g (indra.sources.bel.processor.BelProcessor attribute), 26
Gap (class in indra.statements), 20
gather_explicit_activities() (indra.mechlinker.MechLinker method), 62
gather_implicit_activities() (indra.mechlinker.MechLinker method), 62
Gef (class in indra.statements), 20

gene_list (indra.tools.gene_network.GeneNetwork attribute), 81
GeneNetwork (class in indra.tools.gene_network), 81
Geranylgeranylation (class in indra.statements), 20
get_abstract() (in module indra.literature.elsevier_client), 51
get_abstract() (in module indra.literature.pubmed_client), 50
get_activating_mods() (indra.sources.bel.processor.BelProcessor method), 26
get_activating_subs() (indra.sources.bel.processor.BelProcessor method), 26
get_activation() (indra.sources.bel.processor.BelProcessor method), 27
get_activation() (indra.sources.reach.processor.ReachProcessor method), 36
get_activations() (indra.sources.trips.processor.TripsProcessor method), 37
get_activations_causal() (indra.sources.trips.processor.TripsProcessor method), 37
get_activations_stimulate() (indra.sources.trips.processor.TripsProcessor method), 38
get_active_forms() (indra.sources.trips.processor.TripsProcessor method), 38
get_active_forms_state() (indra.sources.trips.processor.TripsProcessor method), 38
get_activity_modification() (indra.sources.biopax.processor.BiopaxProcessor method), 31
get_agent_rule_str() (in module indra.assemblers.pysb_assembler), 66
get_agents() (indra.sources.ndex_cx.processor.NdexCxProcessor method), 39
get_all_direct_statements() (indra.sources.bel.processor.BelProcessor method), 27
get_all_events() (indra.sources.reach.processor.ReachProcessor method), 36
get_all_events() (indra.sources.trips.processor.TripsProcessor method), 38
get_all_indirect_statements() (indra.sources.bel.processor.BelProcessor method), 27
get_annotation() (in module indra.assemblers.pysb_assembler), 66
get_api_ruler() (indra.sources.reach.reach_reader.ReachReader method), 36
get_article() (in module indra.literature.elsevier_client), 51
get_bel_stmts() (indra.tools.gene_network.GeneNetwork method), 82
get_binding_site_name() (in module indra.assemblers.pysb_assembler), 66
get_biopax_stmts() (indra.tools.gene_network.GeneNetwork method), 82
get_cancer_studies() (in module indra.databases.cbio_client), 46
get_cancer_types() (in module indra.databases.cbio_client), 46
get_case_lists() (in module indra.databases.cbio_client), 47
get_ccle_cna() (in module indra.databases.cbio_client), 47
get_ccle_lines_for_mutation() (in module indra.databases.cbio_client), 47
get_ccle_mrna() (in module indra.databases.cbio_client), 47
get_chebi_id_from_pubchem() (in module indra.databases.chebi_client), 44
get_children() (indra.preassembler.hierarchy_manager.HierarchyManager method), 59
get_complexes() (indra.sources.bel.processor.BelProcessor method), 27
get_complexes() (indra.sources.biopax.processor.BiopaxProcessor method), 31
get_complexes() (indra.sources.reach.processor.ReachProcessor method), 36
get_complexes() (indra.sources.trips.processor.TripsProcessor method), 38
get_composite_activating_mods() (indra.sources.bel.processor.BelProcessor method), 28
get_conversions() (indra.sources.bel.processor.BelProcessor method), 28
get_conversions() (indra.sources.biopax.processor.BiopaxProcessor method), 32
get_create_base_agent() (indra.mechlinker.BaseAgentSet method), 61
get_create_parameter() (in module indra.assemblers.pysb_assembler), 66
get_degenerate_statements() (indra.sources.bel.processor.BelProcessor method), 28
get_degradations() (indra.sources.trips.processor.TripsProcessor method), 38
get_entrez_id() (in module indra.databases.hgnc_client), 40
get_family_members() (in module indra.databases.uniprot_client), 41
get_full_text() (in module indra.literature), 49
get_fulltext_links() (in module indra.literature.crossref_client), 51

get_gap() (indra.sources.biopax.processor.BiopaxProcessor method), 32
get_gef() (indra.sources.biopax.processor.BiopaxProcessor method), 32
get_gene_name() (in module indra.databases.uniprot_client), 41
get_genetic_profiles() (in module indra.databases.cbio_client), 48
get_heat_kernel() (in module indra.databases.relevance_client), 45
get_hgnc_from_entrez() (in module indra.databases.hgnc_client), 40
get_hgnc_from_mouse() (in module indra.databases.hgnc_client), 40
get_hgnc_from_rat() (in module indra.databases.hgnc_client), 40
get_hgnc_id() (in module indra.databases.hgnc_client), 40
get_hgnc_name() (in module indra.databases.hgnc_client), 41
get_id_from_mgi() (in module indra.databases.uniprot_client), 42
get_id_from_mnemonic() (in module indra.databases.uniprot_client), 42
get_id_from_rgd() (in module indra.databases.uniprot_client), 42
get_im() (indra.explanation.model_checker.ModelChecker method), 73
get_metadata_for_ids() (in module indra.literature.pubmed_client), 50
get_mgi_id() (in module indra.databases.uniprot_client), 42
get_mnemonic() (in module indra.databases.uniprot_client), 42
get_mod_site_name() (in module indra.assemblers.pysb_assembler), 66
get_model_agents() (indra.tools.incremental_model.IncrementalModel method), 84
get_modifications() (indra.sources.bel.processor.BelProcessor method), 28
get_modifications() (indra.sources.biopax.processor.BiopaxProcessor method), 32
get_modifications() (indra.sources.reach.processor.ReachProcessor method), 36
get_modifications() (indra.sources.trips.processor.TripsProcessor method), 38
get_monomer_pattern() (in module indra.assemblers.pysb_assembler), 66
get_mouse_id() (in module indra.databases.hgnc_client), 41
get_mouse_id() (in module indra.databases.uniprot_client), 42
get_mutations() (in module indra.databases.cbio_client), 48
get_mutations() (in module indra.databases.context_client), 45
get_mutations_ccle() (in module indra.databases.cbio_client), 48
get_node_names() (indra.sources.ndex_cx.processor.NdexCxProcessor method), 40
get_num_sequenced() (in module indra.databases.cbio_client), 49
get_parents() (indra.preassembler.hierarchy_manager.HierarchyManager method), 59
get_pmids() (indra.sources.ndex_cx.processor.NdexCxProcessor method), 40
get_primary_id() (in module indra.databases.uniprot_client), 42
get_profile_data() (in module indra.databases.cbio_client), 49
get_protein_expression() (in module indra.databases.context_client), 45
get_pubchem_id() (in module indra.databases.chebi_client), 44
get_publications() (in module indra.databases.biogrid_client), 45
get_rat_id() (in module indra.databases.hgnc_client), 41
get_rat_id() (in module indra.databases.uniprot_client), 43
get_regulate_activities() (indra.sources.biopax.processor.BiopaxProcessor method), 32
get_regulate_amounts() (indra.sources.biopax.processor.BiopaxProcessor method), 32
get_regulate_amounts() (indra.sources.reach.processor.ReachProcessor method), 36
get_regulate_amounts() (indra.sources.trips.processor.TripsProcessor method), 38
get_relevant_nodes() (in module indra.databases.relevance_client), 46
get_rgd_id() (in module indra.databases.uniprot_client), 43
get_site_pattern() (in module indra.assemblers.pysb_assembler), 66
get_statements() (indra.sources.ndex_cx.processor.NdexCxProcessor method), 40
get_statements() (indra.tools.gene_network.GeneNetwork method), 82

get_statements() (indra.tools.incremental_model.IncrementalModel method), 84
get_statements_noprior() (indra.tools.incremental_model.IncrementalModel method), 84
get_statements_prior() (indra.tools.incremental_model.IncrementalModel method), 84
get_string() (indra.assemblers.graph_assembler.GraphAssembler method), 69
get_subnetwork() (in module indra.tools.executable_subnetwork), 83
get_syntheses() (indra.sources.trips.processor.TripsProcessor method), 38
get_title() (in module indra.literature.pubmed_client), 50
get_transcription() (indra.sources.bel.processor.BelProcessor method), 29
get_translocation() (indra.sources.reach.processor.ReachProcessor method), 36
get_uncond_agent() (in module indra.assemblers.pysb_assembler), 66
get_uniprot_id() (in module indra.databases.hgnc_client), 41
get_valid_location() (in module indra.statements), 25
get_valid_residue() (in module indra.statements), 25
get_xml() (in module indra.sources.trips.trips_client), 38
Glycosylation (class in indra.statements), 20
graph (indra.assemblers.graph_assembler.GraphAssembler attribute), 69
graph (indra.assemblers.sif_assembler.SifAssembler attribute), 70
graph (indra.preassembler.hierarchy_manager.HierarchyManager attribute), 59
graph_properties (indra.assemblers.graph_assembler.GraphAssembler attribute), 69
graph_query() (in module indra.sources.biopax.pathway_commons_client), 33
GraphAssembler (class in indra.assemblers.graph_assembler), 68
grounded_monomer_patterns() (in module indra.assemblers.pysb_assembler), 66

H

HasActivity (class in indra.statements), 20
hierarchies (indra.preassembler.Preassembler attribute), 52
HierarchyManager (class in indra.preassembler.hierarchy_manager), 58
Hydroxylation (class in indra.statements), 21

I

id_lookup() (in module indra.literature), 49
id_lookup() (in module indra.literature.pmc_client), 50
IncreaseAmount (class in indra.statements), 21
IncrementalModel (class in indra.tools.incremental_model), 83
IndexCardAssembler (class in indra.assemblers.index_card_assembler), 71
indirect_stmts (indra.sources.bel.processor.BelProcessor attribute), 26
indra.assemblers.cx_assembler (module), 67
indra.assemblers.english_assembler (module), 68
indra.assemblers.graph_assembler (module), 68
indra.assemblers.index_card_assembler (module), 71
indra.assemblers.pysb_assembler (module), 64
indra.assemblers.sbgn_assembler (module), 71
indra.assemblers.sif_assembler (module), 70
indra.belief (module), 60
indra.databases.biogrid_client (module), 45
indra.databases.cbio_client (module), 46
indra.databases.chebi_client (module), 44
indra.databases.context_client (module), 45
indra.databases.hgnc_client (module), 40
indra.databases.ndex_client (module), 46
indra.databases.relevance_client (module), 45
indra.databases.uniprot_client (module), 41
indra.explanation.model_checker (module), 72
indra.literature (module), 49
indra.literature.crossref_client (module), 51
indra.literature.elsevier_client (module), 51
indra.literature.pmc_client (module), 50
indra.literature.pubmed_client (module), 50
indra.mechlinker (module), 61
indra.preassembler (module), 51
indra.preassembler.grounding_mapper (module), 56
indra.preassembler.hierarchy_manager (module), 58
indra.preassembler.sitemapper (module), 56
indra.sources.bel.bel_api (module), 25
indra.sources.bel.processor (module), 26
indra.sources.biopax.biopax_api (module), 29
indra.sources.biopax.pathway_commons_client (module), 33
indra.sources.biopax.processor (module), 31
indra.sources.ndex_cx.ndex_cx_api (module), 39
indra.sources.ndex_cx.processor (module), 39
indra.sources.reach.processor (module), 35
indra.sources.reach.reach_api (module), 33
indra.sources.reach.reach_reader (module), 36
indra.sources.trips.processor (module), 37
indra.sources.trips.trips_api (module), 36
indra.sources.trips.trips_client (module), 38
indra.statements (module), 13
indra.tools.assemble_corpus (module), 74
indra.tools.executable_subnetwork (module), 83
indra.tools.gene_network (module), 81
indra.tools.incremental_model (module), 83

infer_activations() (indra.mechlinker.MechLinker static method), 62
infer_active_forms() (indra.mechlinker.MechLinker static method), 62
infer_complexes() (indra.mechlinker.MechLinker static method), 62
infer_modifications() (indra.mechlinker.MechLinker static method), 63
Inhibition (class in indra.statements), 21
InvalidLocationError, 21
InvalidResidueError, 21
is_human() (in module indra.databases.uniprot_client), 43
is_mouse() (in module indra.databases.uniprot_client), 43
is_rat() (in module indra.databases.uniprot_client), 43
is_secondary() (in module indra.databases.uniprot_client), 43
isa() (indra.preassembler.hierarchy_manager.HierarchyManager method), 59

L

LinkedStatement (class in indra.mechlinker), 61
load_prior() (indra.tools.incremental_model.IncrementalModel method), 84
load_site_map() (in module indra.preassembler.sitemapper), 58
load_statements() (in module indra.tools.assemble_corpus), 79
location (indra.mechlinker.AgentState attribute), 61

M

make_model() (indra.assemblers.cx_assembler.CxAssembler method), 67
make_model() (indra.assemblers.english_assembler.EnglishAssembler method), 68
make_model() (indra.assemblers.graph_assembler.GraphAssembler method), 69
make_model() (indra.assemblers.index_card_assembler.IndexCardAssembler method), 71
make_model() (indra.assemblers.pysb_assembler.PysbAssembler method), 65
make_model() (indra.assemblers.sbgn_assembler.SBGNAssembler method), 72
make_model() (indra.assemblers.sif_assembler.SifAssembler method), 70
map_grounding() (in module indra.tools.assemble_corpus), 79
map_sequence() (in module indra.tools.assemble_corpus), 79
map_sites() (indra.preassembler.sitemapper.SiteMapper method), 57
MappedStatement (class in indra.preassembler.sitemapper), 56
max_path_length (indra.explanation.model_checker.PathResult attribute), 74
max_paths (indra.explanation.model_checker.PathResult attribute), 74
MechLinker (class in indra.mechlinker), 61
Methylation (class in indra.statements), 21
ModCondition (class in indra.statements), 22
model (indra.assemblers.english_assembler.EnglishAssembler attribute), 68
model (indra.assemblers.pysb_assembler.PysbAssembler attribute), 64
model (indra.sources.biopax.processor.BiopaxProcessor attribute), 31
model_to_owl() (in module indra.sources.biopax.pathway_commons_client), 33
ModelChecker (class in indra.explanation.model_checker), 72
Modification (class in indra.statements), 22
mods (indra.mechlinker.AgentState attribute), 61
mt (in module indra.statements), 25
mutations (indra.mechlinker.AgentState attribute), 61
MutCondition (class in indra.statements), 22
Myristoylation (class in indra.statements), 23

N

namespace_from_uri() (in module indra.sources.bel.processor), 29
NdexCxProcessor (class in indra.sources.ndex_cx.processor), 39
network_name (indra.assemblers.cx_assembler.CxAssembler attribute), 67
node_properties (indra.assemblers.graph_assembler.GraphAssembler attribute), 69

O

owl_str_to_model() (in module indra.sources.biopax.pathway_commons_client), 33
owl_to_model() (in module indra.sources.biopax.pathway_commons_client), 33

P

Palmitoylation (class in indra.statements), 23
par_to_sec (indra.sources.trips.processor.TripsProcessor attribute), 37
paragraphs (indra.sources.trips.processor.TripsProcessor attribute), 37
parse_identifiers_url() (in module indra.assemblers.pysb_assembler), 66
partof() (indra.preassembler.hierarchy_manager.HierarchyManager method), 59

path_found (indra.explanation.model_checker.PathResult attribute), 73
path_metrics (indra.explanation.model_checker.PathResult attribute), 73
PathMetric (class in indra.explanation.model_checker), 73
PathResult (class in indra.explanation.model_checker), 73
paths (indra.explanation.model_checker.PathResult attribute), 74
Phosphorylation (class in indra.statements), 23
policies (indra.assemblers.pysb_assembler.PysbAssembler attribute), 64
preassemble() (indra.tools.incremental_model.IncrementalModel method), 84
Preassembler (class in indra.preassembler), 51
print_boolean_net() (indra.assemblers.sif_assembler.SifAssembler method), 70
print_cx() (indra.assemblers.cx_assembler.CxAssembler method), 67
print_event_statistics() (indra.sources.reach.processor.ReachProcessor method), 36
print_loopy() (indra.assemblers.sif_assembler.SifAssembler method), 70
print_model() (indra.assemblers.index_card_assembler.IndexCardAssembler method), 71
print_model() (indra.assemblers.pysb_assembler.PysbAssembler method), 65
print_model() (indra.assemblers.sbgn_assembler.SBGNAssembler method), 72
print_model() (indra.assemblers.sif_assembler.SifAssembler method), 70
print_statement_coverage() (indra.sources.bel.processor.BelProcessor method), 29
print_statements() (indra.sources.bel.processor.BelProcessor method), 29
print_statements() (indra.sources.biopax.processor.BiopaxProcessor method), 32
prior_probs (indra.belief.BeliefEngine attribute), 60
process_belrdf() (in module indra.sources.bel.bel_api), 25
process_cx() (in module indra.sources.ndex_cx.ndex_cx_api), 39
process_cx_file() (in module indra.sources.ndex_cx.ndex_cx_api), 39
process_json_file() (in module indra.sources.reach.reach_api), 33
process_json_str() (in module indra.sources.reach.reach_api), 34
process_model() (in module indra.sources.biopax.biopax_api), 29
process_ndex_neighborhood() (in module indra.sources.bel.bel_api), 25
process_ndex_network() (in module indra.sources.ndex_cx.ndex_cx_api), 39
process_nxml_file() (in module indra.sources.reach.reach_api), 34
process_nxml_str() (in module indra.sources.reach.reach_api), 34
process_owl() (in module indra.sources.biopax.biopax_api), 29
process_pc_neighborhood() (in module indra.sources.biopax.biopax_api), 30
process_pc_pathsbetween() (in module indra.sources.biopax.biopax_api), 30
process_pc_pathsfromto() (in module indra.sources.biopax.biopax_api), 30
process_pmc() (in module indra.sources.reach.reach_api), 34
process_pubmed_abstract() (in module indra.sources.reach.reach_api), 35
process_text() (in module indra.sources.reach.reach_api), 35
process_text() (in module indra.sources.trips.trips_api), 36
process_xml() (in module indra.sources.trips.trips_api), 37
protein_map_from_twg() (in module indra.preassembler.grounding_mapper), 56
PysbAssembler (class in indra.assemblers.pysb_assembler), 64

R

ReachProcessor (class in indra.sources.reach.processor), 35
ReachReader (class in indra.sources.reach.reach_reader), 36
reduce_activities() (in module indra.tools.assemble_corpus), 80
reduce_activities() (indra.mechlinker.MechLinker method), 63
RegulateActivity (class in indra.statements), 23
RegulateAmount (class in indra.statements), 23
related_stmts (indra.preassembler.Preassembler attribute), 52
render_stmt_graph() (in module indra.preassembler), 55
replace_activations() (indra.mechlinker.MechLinker method), 63
replace_complexes() (indra.mechlinker.MechLinker method), 63
require_active_forms() (indra.mechlinker.MechLinker method), 63
result_code (indra.explanation.model_checker.PathResult attribute), 73

results (indra.tools.gene_network.GeneNetwork attribute), 82
Ribosylation (class in indra.statements), 23
run_preassembly() (in module indra.tools.assemble_corpus), 80
run_preassembly() (indra.tools.gene_network.GeneNetwork method), 82
run_preassembly_duplicate() (in module indra.tools.assemble_corpus), 80
run_preassembly_related() (in module indra.tools.assemble_corpus), 81

S

save() (indra.tools.incremental_model.IncrementalModel method), 85
save_dot() (indra.assemblers.graph_assembler.GraphAssembler method), 69
save_model() (indra.assemblers.cx_assembler.CxAssembler method), 67
save_model() (indra.assemblers.index_card_assembler.IndexCardAssembler method), 71
save_model() (indra.assemblers.pysb_assembler.PysbAssembler method), 65
save_model() (indra.assemblers.sbgn_assembler.SBGNAssembler method), 72
save_model() (indra.assemblers.sif_assembler.SifAssembler method), 70
save_model() (indra.sources.biopax.processor.BiopaxProcessor method), 32
save_pdf() (indra.assemblers.graph_assembler.GraphAssembler method), 69
save_rst() (indra.assemblers.pysb_assembler.PysbAssembler method), 65
save_xml() (in module indra.sources.trips.trips_client), 38
sbgn (indra.assemblers.sbgn_assembler.SBGNAssembler attribute), 71
SBGNAssembler (class in indra.assemblers.sbgn_assembler), 71
SelfModification (class in indra.statements), 24
send_query() (in module indra.sources.trips.trips_client), 38
send_request() (in module indra.databases.ndex_client), 46
sentences (indra.sources.trips.processor.TripsProcessor attribute), 37
set_base_initial_condition() (in module indra.assemblers.pysb_assembler), 66
set_context() (indra.assemblers.cx_assembler.CxAssembler method), 67
set_context() (indra.assemblers.pysb_assembler.PysbAssembler method), 65
set_extended_initial_condition() (in module indra.assemblers.pysb_assembler), 66
set_hierarchy_probs() (indra.belief.BeliefEngine method), 60
set_linked_probs() (indra.belief.BeliefEngine method), 60
set_prior_probs() (indra.belief.BeliefEngine method), 60
SifAssembler (class in indra.assemblers.sif_assembler), 70
SiteMapper (class in indra.preassembler.sitemapper), 56
Statement (class in indra.statements), 24
statements (indra.assemblers.cx_assembler.CxAssembler attribute), 67
statements (indra.assemblers.english_assembler.EnglishAssembler attribute), 68
statements (indra.assemblers.graph_assembler.GraphAssembler attribute), 69
statements (indra.assemblers.index_card_assembler.IndexCardAssembler attribute), 71
statements (indra.assemblers.pysb_assembler.PysbAssembler attribute), 64
statements (indra.assemblers.sbgn_assembler.SBGNAssembler attribute), 71
statements (indra.sources.bel.processor.BelProcessor attribute), 26
statements (indra.sources.biopax.processor.BiopaxProcessor attribute), 31
statements (indra.sources.ndex_cx.processor.NdexCxProcessor attribute), 39
statements (indra.sources.reach.processor.ReachProcessor attribute), 36
statements (indra.sources.trips.processor.TripsProcessor attribute), 37
stmts (indra.preassembler.Preassembler attribute), 51
stmts (indra.tools.incremental_model.IncrementalModel attribute), 84
strip_agent_context() (in module indra.tools.assemble_corpus), 81
Sumoylation (class in indra.statements), 24

T

term_from_uri() (in module indra.sources.bel.processor), 29
to_graph() (indra.statements.Statement method), 24
to_json() (indra.statements.Statement method), 24
Translocation (class in indra.statements), 24
Transphosphorylation (class in indra.statements), 24
tree (indra.sources.reach.processor.ReachProcessor attribute), 36
tree (indra.sources.trips.processor.TripsProcessor attribute), 37
TripsProcessor (class in indra.sources.trips.processor), 37

U

Ubiquitination (class in indra.statements), 25

unique_stmts (indra.preassembler.Preassembler attribute), 51
upload_model() (indra.assemblers.cx_assembler.CxAssembler method), 68

V

verify_location() (in module indra.databases.uniprot_client), 43
verify_modification() (in module indra.databases.uniprot_client), 44
